# Four-dimensional Fano quiver flag zero loci

**DOI:** 10.1098/rspa.2018.0791

**Published:** 2019-05-15

**Authors:** Elana Kalashnikov

**Affiliations:** Department of Mathematics, Imperial College London, 180 Queen's Gate, London SW7 2AZ, UK

**Keywords:** Fano manifolds, mirror symmetry, quantum differential equations, Picard–Fuchsequations, quiver flag varieties

## Abstract

Quiver flag zero loci are subvarieties of quiver flag varieties cut out by sections of representation theoretic vector bundles. We prove the Abelian/non-Abelian correspondence in this context: this allows us to compute genus zero Gromov–Witten invariants of quiver flag zero loci. We determine the ample cone of a quiver flag variety, and disprove a conjecture of Craw. In the appendices (which can be found in the electronic supplementary material), which are joint work with Tom Coates and Alexander Kasprzyk, we use these results to find four-dimensional Fano manifolds that occur as quiver flag zero loci in ambient spaces of dimension up to 8, and compute their quantum periods. In this way, we find at least 141 new four-dimensional Fano manifolds.

## 1. Introduction

Quiver flag varieties are a generalization of type A flag varieties that were introduced by Craw [1] based on work of King [2]. They are fine moduli spaces for stable representations of the associated quiver (see §§2c). Like flag varieties and toric complete intersections, quiver flag varieties can be constructed as GIT quotients of a vector space (see §2a). Unlike toric varieties, the quotienting group for a quiver flag variety is in general non-Abelian; this increases the complexity of their structure considerably: specifically, it places them largely outside of the range of known mirror symmetry constructions.

These two perspectives on quiver flag varieties—as fine moduli spaces and as GIT quotients—give two different ways to consider them as ambient spaces. From the moduli space perspective, smooth projective varieties with collections of vector bundles together with appropriate maps between them come with natural maps into the quiver flag variety. From the GIT perspective, one is led to consider subvarieties which occur as zero loci of sections of representation theoretic vector bundles. If the ambient GIT quotient is a toric variety, these subvarieties are toric complete intersections; if the ambient space is a quiver flag variety, we call these subvarieties *quiver flag zero loci*. While in this paper, we emphasize the GIT quotient perspective, the moduli space perspective should be kept in mind as further evidence of the fact that quiver flag varieties are natural ambient spaces. All smooth Fano varieties of dimension less than or equal to three can be constructed as either toric complete intersections or quiver flag zero loci. These constructions of the Fano threefolds were given in [3]: see theorem A.1 in [3] as well as the explicit constructions in each case. While there is an example in dimension 66 of a Fano variety which is neither a toric complete intersection nor a quiver flag zero locus, one might nevertheless hope that most four-dimensional smooth Fano varieties are either toric complete intersections or quiver flag zero loci. The classification of four-dimensional Fano varieties is open.

This paper studies quiver flag varieties with a view towards understanding them as ambient spaces of Fano fourfolds. Specifically [4] classified smooth four-dimensional Fano toric complete intersections with codimension at most four in the ambient space. This heavily computational search relied on understanding the geometry and quantum cohomology of toric varieties from their combinatorial structure. The guiding motivation of the body of the paper is to establish comparable results for quiver flag varieties to enable the same search to be carried out in this context. For example, we determine the ample cone of a quiver flag variety from the path space of the associated quiver: in this way, we are able to efficiently determine a sufficient condition for whether a quiver flag zero locus is Fano.

The main result of this paper is the proof of the Abelian/non-Abelian correspondence of Ciocan–Fontanine–Kim–Sabbah for Fano quiver flag zero loci. This allows us to compute their genus zero Gromov–Witten invariants.^1^ From the perspective of the search for four-dimensional Fano quiver flag zero loci, the importance of this result is that it allows us to compute the quantum period. The quantum period (a generating function built out of certain genus 0 Gromov–Witten invariants) is the invariant that we use to distinguish deformation families of Fano fourfolds: if two quiver flag zero loci have different period sequences, they are not deformation equivalent. The appendices in the electronic supplementary, joint work with Coates and Kasprzyk, describe the search and its results.

Our primary motivation for these results is as follows. There has been much recent interest in the possibility of classifying Fano manifolds using mirror symmetry. It is conjectured that, under mirror symmetry, *n*-dimensional Fano manifolds should correspond to certain very special Laurent polynomials in *n* variables [6]. This conjecture has been established in dimensions up to three [3], where the classification of Fano manifolds is known [7,8]. Little is known about the classification of four-dimensional Fano manifolds, but there is strong evidence that the conjecture holds for four-dimensional toric complete intersections [4]. The results of the appendices will provide a first step towards establishing the conjectures for these four-dimensional Fano quiver flag zero loci.

**Figure 1.**
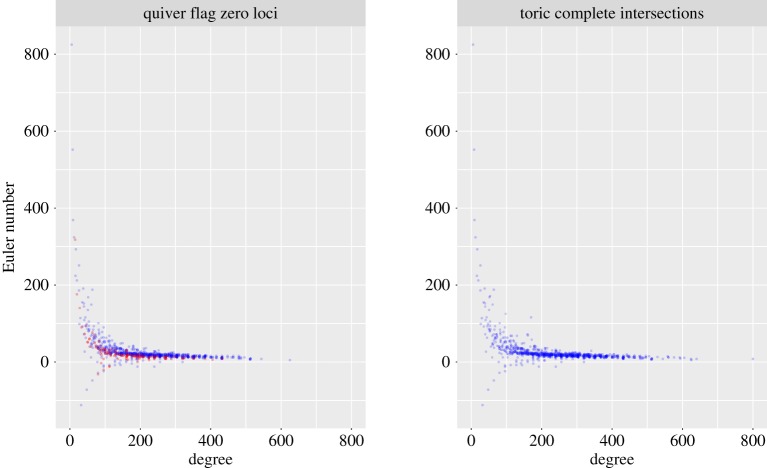
Degrees and Euler numbers for four-dimensional Fano quiver flag zero loci and toric complete intersections; cf. [4, fig. 5]. Quiver flag zero loci that are not toric complete intersections are highlighted in red. (Online version in colour.)

In the appendices in the electronic supplementary material, which are joint work with Tom Coates and Alexander Kasprzyk, we use the structure theory developed here to find four-dimensional Fano manifolds that occur as quiver flag zero loci in ambient spaces of dimension up to 8, and compute their quantum periods. One hundred and forty-one of these quantum periods were previously unknown. Thus we find at least 141 new four-dimensional Fano manifolds. This computation is described in the appendices. The quantum periods, and quiver flag zero loci that give rise to them, are also recorded there. Figure 1 shows the distribution of degree and Euler number for the four-dimensional quiver flag zero loci that we found, and for four-dimensional Fano toric complete intersections.

## 2. Quiver flag varieties

Quiver flag varieties are generalizations of Grassmannians and type A flag varieties [1]. Like flag varieties, they are GIT quotients and fine moduli spaces. We begin by recalling Craw's construction. A quiver flag variety *M*(*Q*, **r**) is determined by a quiver *Q* and a dimension vector **r**. The quiver *Q* is assumed to be finite and acyclic, with a unique source. Let *Q*_0_ = {0, 1, …, *ρ*} denote the set of vertices of *Q* and let *Q*_1_ denote the set of arrows. Without loss of generality, after reordering the vertices if necessary, we may assume that 0∈*Q*_0_ is the unique source and that the number *n*_*ij*_ of arrows from vertex *i* to vertex *j* is zero unless *i* < *j*. Write *s*, *t*:*Q*_1_ → *Q*_0_ for the source and target maps, so that an arrow *a*∈*Q*_1_ goes from *s*(*a*) to *t*(*a*). The dimension vector **r** = (*r*_0_, …, *r*_*ρ*_) lies in $N^{\rho +1}$, and we insist that *r*_0_ = 1. *M*(*Q*, **r**) is defined to be the moduli space of *θ*-stable representations of the quiver *Q* with dimension vector **r**. Here *θ* is a fixed stability condition defined below, determined by the dimension vector.

### (a) Quiver flag varieties as GIT quotients

Consider the vector space

$$\mathrm{Rep}(Q,r)=\underset{a\in Q_{1}}{⨁}\mathrm{Hom}(C^{r_{s(a)}},C^{r_{t(a)}}),$$

and the action of $\mathrm{GL}(r):=\prod_{i=0}^{\rho }\mathrm{GL}(r_{i})$ on Rep(*Q*, **r**) by change of basis. The diagonal copy of GL(1) in GL(**r**) acts trivially, but the quotient *G*: = GL(**r**)/GL(1) acts effectively; since *r*_0_ = 1, we may identify *G* with $\prod_{i=1}^{\rho }\mathrm{GL}(r_{i}).$ The quiver flag variety *M*(*Q*, **r**) is the GIT quotient Rep(*Q*, **r**)//_*θ*_ *G*, where the stability condition *θ* is the character of *G* given by

$$\theta (g)=\prod_{i=1}^{\rho }\det(g_{i})\;\mathrm{and}\;g=(g_{1},\ldots ,g_{\rho })\in \prod_{i=1}^{\rho }\mathrm{GL}(r_{i}).$$

For the stability condition *θ*, all semistable points are stable. To identify the *θ*-stable points in Rep(*Q*, **r**), set $s_{i}=\sum_{a\in Q_{1},t(a)=i}r_{s(a)}$ and write

$$\mathrm{Rep}(Q,r)=\overset{\rho }{\underset{i=1}{⨁}}\mathrm{Hom}(C^{s_{i}},C^{r_{i}}).$$

Then *w* = (*w*_*i*_)^*ρ*^_*i*=1_ is *θ*-stable if and only if *w*_*i*_ is surjective for all *i*.

**Example 2.1**

Consider the quiver *Q* given by

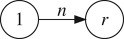

so that *ρ* = 1, *n*_01_ = *n*, and the dimension vector **r** = (1, *r*). Then $\mathrm{Rep}(Q,r)=\mathrm{Hom}(C^{n},C^{r})$, and the *θ*-stable points are surjections $C^{n}\to C^{r}$. The group *G* acts by change of basis, and therefore *M*(*Q*, **r**) = Gr(*n*, *r*), the Grassmannian of *r*-dimensional quotients of $C^{n}$. More generally, the quiver

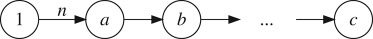

gives the flag of quotients Fl(*n*, *a*, *b*, …, *c*).

Quiver flag varieties are non-Abelian GIT quotients unless the dimension vector **r** = (1, 1, …, 1). In this case, $G≅\prod_{i=1}^{\rho }{\mathrm{GL}}_{1}(C)$ is Abelian, and *M*(*Q*;**r**) is a toric variety. We call such *M*(*Q*, **r**) toric quiver flag varieties. Not all toric varieties are toric quiver flag varieties.

### (b) Quiver flag varieties as ambient spaces: Quiver flag zero loci

As mentioned in the introduction, GIT quotients have a special class of subvarieties, sometimes called representation theoretic subvarieties. In this subsection, we discuss these subvarieties in the specific case of quiver flag varieties.

We have expressed the quiver flag variety *M*(*Q*, **r**) as the quotient by *G* of the semistable locus Rep(*Q*, **r**)^*ss*^⊂Rep(*Q*, **r**). A representation *E* of *G*, therefore, defines a vector bundle *E*_*G*_ → *M*(*Q*, **r**) with fibre *E*; here *E*_*G*_ = *E* × _*G*_Rep(*Q*, **r**)^*ss*^. In the appendix in the electronic supplementary material, we will study subvarieties of quiver flag varieties cut out by regular sections of such bundles. If *E*_*G*_ is globally generated, a generic section cuts out a smooth subvariety. We refer to such subvarieties as *quiver flag zero loci*, and such bundles as representation theoretic bundles. As mentioned above, quiver flag varieties can also be considered natural ambient spaces via their moduli space construction [1,9].

The representation theory of $G=\prod_{i=1}^{\rho }\mathrm{GL}(r_{i})$ is well-understood, and we can use this to write down the bundles *E*_*G*_ explicitly. Irreducible polynomial representations of GL(*r*) are indexed by partitions (or Young diagrams) of length at most *r*. The irreducible representation corresponding to a partition *α* is the Schur power $S^{\alpha }C^{r}$ of the standard representation of GL(*r*) [10, ch. 8]. For example, if *α* is the partition (*k*) then $S^{\alpha }C^{r}={\mathrm{Sym}}^{k}C^{r}$, the *k*th symmetric power, and if *α* is the partition (1, 1, …, 1) of length *k* then $S^{\alpha }C^{r}=\overset{k}{⋀}C^{r}$, the *k*th exterior power. Irreducible polynomial representations of *G* are therefore indexed by tuples (*α*_1_, …, *α*_*ρ*_) of partitions, where *α*_*i*_ has length at most *r*_*i*_. The tautological bundles on a quiver flag variety are representation theoretic: if $E=C^{r_{i}}$ is the standard representation of the *i*th factor of *G*, then *W*_*i*_ = *E*_*G*_. More generally, the representation indexed by (*α*_1_, …, *α*_*ρ*_) is $\overset{\rho }{\underset{i=1}{⨂}}S^{\alpha_{i}}C^{r_{i}}$, and the corresponding vector bundle on *M*(*Q*, **r**) is $\overset{\rho }{\underset{i=1}{⨂}}S^{\alpha_{i}}W_{i}$.

**Example 2.2**

Consider the vector bundle Sym^2^*W*_1_ on Gr(8, 3). By duality—which sends a quotient $C^{8}\to V\to 0$ to a subspace $0\to V^{*}\to {\left(C^{8}\right)}^{*}$—this is equivalent to considering the vector bundle Sym^2^S*_1_ on the Grassmannian of three-dimensional subspaces of ${\left(C^{8}\right)}^{*}$, where *S*_1_ is the tautological subbundle. A generic symmetric 2-form *ω* on ${\left(C^{8}\right)}^{*}$ determines a regular section of Sym^2^S*_1_, which vanishes at a point V* if and only if the restriction of *ω* to V* is identically zero. So the associated quiver flag zero locus is the orthogonal Grassmannian OGr(3, 8).

### (c) Quiver flag varieties as moduli spaces

To give a morphism to *M*(*Q*, **r**) from a scheme *B* is the same as to give

— globally generated vector bundles *W*_*i*_ → *B*, *i*∈*Q*_0_, of rank *r*_*i*_ such that $W_{0}=O_{B}$; and
— morphisms *W*_*s*(*a*)_ → *W*_*t*(*a*)_, *a*∈*Q*_1_ satisfying the *θ*-stability condition

up to isomorphism. Thus *M*(*Q*, **r**) carries universal bundles *W*_*i*_, *i*∈*Q*_0_. It is also a Mori dream space (see proposition 3.1 in [1]). The GIT description gives an isomorphism between the Picard group of *M*(*Q*, **r**) and the character group $\chi (G)≅Z^{\rho }$ of *G*. When tensored with Q, the fact that this is a Mori dream space (see lemma 4.2 in [11]) implies that this isomorphism induces an isomorphism of wall and chamber structures given by the Mori structure (on the effective cone) and the GIT structure (on χ(G)⊗Q); in particular, the GIT chamber containing *θ* is the ample cone of *M*(*Q*, **r**). The Picard group is generated by the determinant line bundles $\det(W_{i})$, *i*∈*Q*_0_.

### (d) Quiver flag varieties as towers of Grassmannian bundles

Generalizing example 2.1, all quiver flag varieties are towers of Grassmannian bundles [1, theorem 3.3]. For 0 ≤ *i* ≤ *ρ*, let *Q*(*i*) be the subquiver of *Q* obtained by removing the vertices *j*∈*Q*_0_, *j* > *i*, and all arrows attached to them. Let **r**(*i*) = (1, *r*_1_, …, *r*_*i*_), and write *Y*_*i*_ = *M*(*Q*(*i*), **r**(*i*)). Denote the universal bundle *W*_*j*_ → *Y*_*i*_ by *W*^(*i*)^_*j*_. Then there are maps

$$M(Q,r)=Y_{\rho }\to Y_{\rho -1}\to \cdots \to Y_{1}\to Y_{0}=\mathrm{Spec}C,$$

induced by isomorphisms $Y_{i}≅\mathrm{Gr}(F_{i},r_{i})$, where $F_{i}$ is the locally free sheaf

$$F_{i}=\underset{a\in Q_{1},t(a)=i}{⨁}W_{s(a)}^{\left(i-1\right)}$$

of rank *s*_*i*_ on *Y*_*i*−1_. This makes clear that *M*(*Q*, **r**) is a smooth projective variety of dimension $\sum_{i=1}^{\rho }r_{i}(s_{i}-r_{i})$, and that *W*_*i*_ is the pullback to *Y*_*ρ*_ of the tautological quotient bundle over $\mathrm{Gr}(F_{i},r_{i})$. Thus *W*_*i*_ is globally generated, and $\det(W_{i})$ is nef. Furthermore, the anti-canonical line bundle of *M*(*Q*, **r**) is

2.1 $$\underset{a\in Q_{1}}{⨂}\det{\left(W_{t(a)}\right)}^{r_{s(a)}}⊗\det{\left(W_{s(a)}\right)}^{-r_{t(a)}}.$$

In particular, *M*(*Q*, **r**) is Fano if $s_{i}>s_{i}^{\prime }:=\sum_{a\in Q_{1},s(a)=i}r_{t(a)}.$ This condition is not if and only if.

### (e) The Euler sequence

Quiver flag varieties, like both Grassmannians and toric varieties, have an Euler sequence.

**Proposition 2.3**

*Let* *X* = *M*(*Q*, **r**) *be a quiver flag variety, and for* *a*∈*Q*_1_, *denote* *W*_*a*_: = W*_s(*a*)_⊗*W*_*t*(*a*)_. *There is a short exact sequence*

$$0\to \overset{\rho }{\underset{i=1}{⨁}}W_{i}^{*}⊗W_{i}\to \underset{a\in Q_{1}}{⨁}W_{a}\to T_{X}\to 0.$$

**Proof.**

We proceed by induction on the Picard rank *ρ* of *X*. If *ρ* = 1 then this is the usual Euler sequence for the Grassmannian. Suppose that the proposition holds for quiver flag varieties of Picard rank *ρ* − 1, for *ρ* > 1. Then the fibration $\pi :\mathrm{Gr}(\pi^{*}F_{\rho },r_{\rho })\to Y_{\rho -1}$ from §2d gives a short exact sequence

$$0\to W_{\rho }^{*}⊗W_{\rho }\to \pi^{*}F_{\rho }^{*}⊗W_{\rho }\to S^{*}⊗W_{\rho }\to 0,$$

where *S* is the kernel of the projection $\pi^{*}F_{\rho }\to W_{\rho }$. Note that

$$\pi^{*}F_{\rho }^{*}⊗W_{\rho }=\underset{a\in Q_{1},t(a)=\rho }{⨁}W_{a}\;\mathrm{andthat}\;T_{X}=T_{Y_{\rho -1}}⊕S^{*}⊗W_{\rho }.$$

As S*⊗*W*_*ρ*_ is the relative tangent bundle to *π*, the proposition follows by induction. ▪

If *X* is a quiver flag zero locus cut out of the quiver flag variety *M*(*Q*, **r**) by a regular section of the representation theoretic vector bundle *E* then there is a short exact sequence

2.2 $$0\to T_{X}\to T_{M(Q,r)}{|}_{X}\to E\to 0.$$

Thus *T*_*X*_ is the K-theoretic difference of representation theoretic vector bundles.

## 3. Quiver flag varieties as subvarieties

There are three well-known constructions of flag varieties: as GIT quotients, as homogeneous spaces and as subvarieties of products of Grassmannians. Craw's construction gives quiver flag varieties as GIT quotients. General quiver flag varieties are not homogeneous spaces, so the second construction does not generalize. In this section, we generalize the third construction of flag varieties, exhibiting quiver flag varieties as subvarieties of products of Grassmannians. It is this description that will allow us to prove the Abelian/non-Abelian correspondence for quiver flag varieties.

**Proposition 3.1**

*Let* *M*_*Q*_: = *M*(*Q*, **r**) *be a quiver flag variety with* *ρ* > 1. *Then* *M*_*Q*_ *is cut out of* $Y=\prod_{i=1}^{\rho }\mathrm{Gr}(H^{0}(M_{Q},W_{i}),r_{i})$ *by a tautological section of*

$$E=\underset{a\in Q_{1},s(a)\neq 0}{⨁}S_{s(a)}^{*}⊗Q_{t(a)},$$

*where* *S*_*i*_ *and* *Q*_*i*_ *are the pullbacks to* *Y* *of the tautological sub-bundle and quotient bundle on the* *i*^*th*^ *factor of* *Y* .

**Proof.**

As vector spaces, there is an isomorphism $H^{0}(M_{Q},W_{i})≅e_{0}CQe_{i}$, where CQ is the path algebra over C of *Q* (corollary 3.5, [1]). This isomorphism identifies a basis of global sections of *W*_*i*_ from the set of paths from vertex 0 to *i* in the quiver. Let $e_{a}\in CQ$ be the element associated with the arrow *a*∈*Q*_1_. Thus

$$H^{0}(M_{Q},W_{i})=\underset{a\in Q_{1},t(a)=i,s(a)\neq 0}{⨁}H^{0}(M_{Q},W_{s(a)})⊕\underset{a\in Q_{1},s(a)=0,t(a)=i}{⨁}Ce_{a}.$$

Let $F_{i}=\underset{t(a)=i}{⨁}Q_{s(a)}$. Combining the tautological surjective morphisms

$$H^{0}(M_{Q},W_{s(a)})⊗O_{Y}=H^{0}(Y,Q_{s(a)})⊗O_{Y}\to Q_{s(a)},$$

gives the exact sequence

$$0\to \underset{t(a)=i,s(a)\neq 0}{⨁}S_{s(a)}\to H^{0}(M_{Q},W_{i})⊗O_{Y}\to F_{i}\to 0.$$

Thus

$$\frac{\left(H^{0}{\left(M_{Q},W_{i}\right)}^{*}⊗O_{Y}\right)}{F_{i}^{*}}≅\underset{t(a)=i,s(a)\neq 0}{⨁}S_{s(a)}^{*},$$

and it follows that $E=\overset{\rho }{\underset{i=2}{⨁}}\mathrm{Hom}(Q_{i}^{*},(H^{0}{\left(M_{Q},W_{i}\right)}^{*}⊗O_{Y})/F_{i}^{*})$.

Consider the section *s* of *E* given by the compositions

$$Q_{i}^{*}\to H^{0}{\left(M_{Q},W_{i}\right)}^{*}⊗O_{Y}\to \frac{\left(H^{0}{\left(M_{Q},W_{i}\right)}^{*}⊗O_{Y}\right)}{F_{i}^{*}}.$$

The section *s* vanishes at quotients (*V*_1_, …, *V*_*ρ*_) if and only if $V_{i}^{*}\subset \underset{t(a)=i}{⨁}V_{s(a)}^{*}$; dually, the zero locus is where there is a surjection *F*_*i*_ → *Q*_*i*_ for each *i*. We now identify *Z*(*s*) with *M*(*Q*, **r**). Since the *W*_*i*_ are globally generated, there is a unique map

$$f:M_{Q}\to Y=\prod_{i=1}^{\rho }\mathrm{Gr}(H^{0}(M_{Q},W_{i}),r_{i}),$$

such that *Q*_*i*_ → *Y* pulls back to *W*_*i*_ → *M*(*Q*, **r**). By construction of *M*_*Q*_ there are surjections

$${⊕}_{a\in Q_{1},t(a)=i}Q_{s(a)}\to W_{i}\to 0,$$

so *f*(*M*_*Q*_)⊂*Z*(*s*).

Any variety *X* with vector bundles *V*_*i*_ of rank *r*_*i*_ for *i* = 1, …, *ρ* and maps *H*^0^(*M*_*Q*_, *W*_*i*_) → *V*_*i*_ → 0 that factor as

$$H^{0}(M_{Q},W_{i})\to \underset{t(a)=i}{⨁}V_{s(a)}\to V_{i}$$

has a unique map to *M*(*Q*, **r**) as the *V*_*i*_ form a flat family of *θ*-stable representations of *Q* of dimension **r**. The (*Q*_*i*_|_*Z*(*s*)_)^*ρ*^_*i*=1_ on *Z*(*s*) give precisely such a set of vector bundles. The surjections *H*^0^(*M*_*Q*_, *W*_*i*_) → *Q*_*i*_|_*Z*(*s*)_ → 0 follow from the fact that these are restrictions of the tautological bundles on a product of Grassmannians. That these maps factor as required is precisely the condition that *s* vanishes.

Let *g*:*Z*(*s*) → *M*_*Q*_ be the induced map. By the universal property of *M*(*Q*, **r**), the composition *g*°*f*:*M*_*Q*_ → *Z*(*s*) → *M*_*Q*_ must be the identity. The composition *f*°*g*:*Z*(*s*) → *M*(*Q*, **r**) → *Y* must be the inclusion *Z*(*s*) → *Y* by the universal property of *Y* . Therefore, *Z*(*s*) and *M*(*Q*, **r**) are canonically isomorphic. ▪

Suppose that *X* is a quiver flag zero locus cut out of *M*(*Q*, **r**) by a regular section of a representation theoretic vector bundle *E*_*G*_ determined by a representation *E*. The product of Grassmannians $Y=\prod_{i=1}^{\rho }\mathrm{Gr}(H^{0}(W_{i}),r_{i})$ is a GIT quotient *V*^*ss*^/*G* for the same group *G* (one can see this by constructing *Y* as a quiver flag variety). Therefore, *E* also determines a vector bundle *E*′_*G*_ on *Y* :

$$E_{G}^{\prime }:=E\times \frac{V^{ss}}{G}\to Y.$$

We see that *X* is deformation equivalent to the zero locus of a generic section of the vector bundle

3.1 $$F:=E_{G}^{\prime }⊕\underset{a\in Q_{1},s(a)\neq 0}{⨁}S_{s(a)}^{*}⊗Q_{t(a)},$$

Although *Y* is a quiver flag variety, this is not generally an additional model of *X* as a quiver flag zero locus, as the summand S*_s(*a*)_⊗*Q*_*t*(*a*)_ in *F* does not in general come from a representation of *G*. We refer to the summands of *F* of this form as *arrow bundles*.

**Remark 3.2**

Suppose *α* is a non-negative Schur partition. Then [12] shows that *S*^*α*^(*Q*_*i*_) is globally generated on *Y* (using the notation as above). This implies that *S*^*α*^(*W*_*i*_) is globally generated on *M*(*Q*, **r**).

## 4. Equivalences of quiver flag zero loci

The representation of a given variety *X* as a quiver flag zero locus, if it exists, is far from unique. In this section, we describe various methods of passing between different representations of the same quiver flag zero locus. This is important in practice, because our systematic search for four-dimensional quiver flag zero loci described in the appendices in the electronic supplementary material finds a given variety in many different representations. Furthermore, geometric invariants of a quiver flag zero locus *X* can be much easier to compute in some representations than in others. The observations in this section allow us to compute invariants of four-dimensional Fano quiver flag zero loci using only a few representations, where the computation is relatively cheap, rather than doing the same computation many times and using representations where the computation is expensive (see the appendices in the electronic supplementary material for more details). The results of this section are only used in the appendices in the electronic supplementary material: the rest of the paper is independent.

### (a) Dualizing

As we saw in the previous section, a quiver flag zero locus *X* given by (*M*(*Q*, **r**), *E*) can be thought of as a zero locus in a product of Grassmannians *Y* . Unlike general quiver flag varieties, Grassmannians come in canonically isomorphic dual pairs:


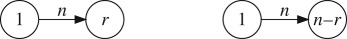


The isomorphism interchanges the tautological quotient bundle *Q* with S*, where *S* is the tautological sub-bundle. One can then dualize some or none of the Grassmannian factors in *Y* , to get different models of *X*. Depending on the representations in *E*, after dualizing, *E* may still be a representation theoretic vector bundle, or the direct sum of a representation theoretic vector bundle with bundles of the form S*_i_⊗*W*_*j*_. If this is the case, one can then undo the product representation process to obtain another model (*M*(*Q*′, **r**′), *E*′_*G*_) of *X*.

**Example 4.1**

Consider *X* given by the quiver

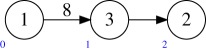

and bundle ∧^2^*W*_2_; here and below the vertex numbering is indicated in blue. Then writing it as a product:

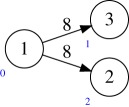

with bundle ∧^2^*W*_2_⊕S*_1_⊗*W*_2_ (as in equation (3.1)) and dualizing the first factor, we get

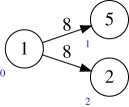

with bundle ∧^2^*W*_2_⊕*W*_1_⊗*W*_2_, which is a quiver flag zero locus.

### (b) Removing arrows

**Example 4.2**

Note that Gr(*n*, *r*) is the quiver flag zero locus given by (Gr(*n* + 1, *r*), *W*_1_). This is because the space of sections of *W*_1_ is $C^{n+1}$, where the image of the section corresponding to $v\in C^{n+1}$ at the point $\phi :C^{n+1}\to C^{r}$ in Gr(*n* + 1, *r*) is *ϕ*(*v*). This section vanishes precisely when v∈kerϕ, so we can consider its zero locus to be $\mathrm{Gr}(C^{n+1}/\langle v\rangle ,r)≅\mathrm{Gr}(n,r)$. The restriction of *W*_1_ to this zero locus Gr(*n*, *r*) is *W*_1_, and the restriction of the tautological sub-bundle *S* is $S⊕O_{\mathrm{Gr}(n,r)}$.

This example generalizes. Let *M*(*Q*, **r**) be a quiver flag variety. A choice of arrow *i* → *j* in *Q* determines a canonical section of W*_i_⊗*W*_*j*_, and the zero locus of this section is *M*(*Q*′, **r**), where *Q*′ is the quiver obtained from *Q* by removing one arrow from *i* → *j*.

**Example 4.3**

Similarly, Gr(*n*, *r*) is the zero locus of a section of S*, the dual of the tautological sub-bundle, on Gr(*n* + 1, *r* + 1). The exact sequence $0\to W_{1}^{*}\to {\left(C^{n+1}\right)}^{*}\to S^{*}\to 0$ shows that a global section of S* is given by a linear map $\psi :C^{n+1}\to C$. The image of the section corresponding to *ψ* at the point *s*∈*S* is *ψ*(*s*), where we evaluate *ψ* on *s* via the tautological inclusion $S\to C^{n+1}$. Splitting $C^{n+1}=C^{n}⊕C$ and choosing *ψ* to be projection to the second factor shows that *ψ* vanishes precisely when $S\subset C^{n}$, that is precisely along Gr(*n*, *r*). The restriction of *S* to this zero locus Gr(*n*, *r*) is *S*, and the restriction of *W*_1_ is $W_{1}⊕O_{\mathrm{Gr}(n,r)}$.

### (c) Grafting

Let *Q* be a quiver. We say that *Q* is *graftable* at *i*∈*Q*_0_ if

— *r*_*i*_ = 1 and 0 < *i* < *ρ*;
— if we remove all of the arrows out of *i* we get a disconnected quiver.

Call the quiver with all arrows out of *i* removed *Q*^*i*^. If *i* is graftable, we call the *grafting set* of *i*

$$\{j\in Q_{0}|0\;\mathrm{and}\;j\;\mathrm{are in different components of}\;Q^{i}\}.$$

**Example 4.4**

In the quiver below, vertex 1 is not graftable.

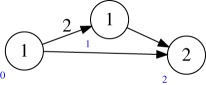

If we removed the arrow from vertex 0 to vertex 2, then vertex 1 would be graftable and the grafting set would be {2}.

**Proposition 4.5**

*Let* *M*(*Q*, **r**) *be a quiver flag variety and let* *i* *be a vertex of* *Q* *that is graftable. Let* *J* *be its grafting set. Let* *Q*′ *be the quiver obtained from* *Q* *by replacing each arrow* *i* → *j*, *where* *j*∈*J*, *by an arrow* 0 → *j*. *Then*

$$M(Q,r)=M(Q^{\prime },r).$$

**Proof.**

Define *V*_*j*_: = W*_i_⊗*W*_*j*_ for *j*∈*J*, and *V*_*j*_: = *W*_*j*_ otherwise.

Note that by construction of *J*, for *j*∈*J*, there is a surjective morphism

$$W_{i}^{⊕d_{ij}}\to W_{j}\to 0.$$

Here, *d*_*ij*_ is the number of paths *i* → *j*. Tensoring this sequence with W*_i_ shows that *V*_*j*_ is globally generated.

Now we show that the *V*_*j*_, *j*∈{0, …, *ρ*} are a *θ*-stable representation of *Q*′. It suffices to check that there are surjective morphisms

$$\underset{a\in Q_{1}^{\prime },t(a)=j}{⨁}V_{s(a)}\to V_{j}.$$

If *j*∉*J*, this is just the same surjection given by the fact that the *W*_*i*_ are a *θ*-stable representation of *Q*. If *j*∈*J*, one must, as above, tensor the sequence from *Q* with W*_i_. The *V*_*j*_ then give a map *M*(*Q*, **r**) → *M*(*Q*′, **r**). Reversing this procedure shows that this is a canonical isomorphism. ▪

**Example 4.6**

Consider the quiver flag zero locus *X* given by the quiver in (a) below, with bundle

$$W_{1}⊗W_{3}⊕W_{1}^{⊕2}⊕\det W_{1}.$$

Note we have chosen a different labelling of the vertices for convenience. Writing *X* inside a product of Grassmannians gives $W_{1}⊗W_{3}⊕W_{1}^{⊕2}⊕\det W_{1}$ on the quiver in (b), with arrow bundle S*_2_⊗*W*_1_. Removing the two copies of *W*_1_ using example 4.2 gives

$$W_{1}⊗W_{3}⊕\det W_{1},$$

on the quiver in (c), with arrow bundle S*_2_⊗*W*_1_. We now apply example 4.3 to remove $\det W_{1}=\det S_{1}^{*}=S_{1}^{*}$. As mentioned in example 4.3, *W*_1_ on (c) becomes $W_{1}⊕O$ after removing S*_1_. The arrow bundle therefore becomes

$$S_{2}^{*}⊗(W_{1}⊕O)=S_{2}^{*}⊕S_{2}^{*}⊗W_{1}.$$

Similarly, *W*_1_⊗*W*_3_ becomes *W*_3_⊕*W*_1_⊗*W*_3_. We can remove the new S*_2_ and *W*_3_ summands (reducing the Gr(8, 6) factor to Gr(7, 5) and the *Gr*(8, 2) factor to Gr(7, 2), respectively). Thus, we see that *X* is given by *W*_1_⊗*W*_3_ on the quiver in (d), with arrow bundle S*_2_⊗*W*_1_. Dualizing at vertices 1 and 2 now gives the quiver in (e), with arrow bundle S*_1_⊗*W*_2_⊕S*_1_⊗*W*_3_. Finally, undoing the product representation exhibits *X* as the quiver flag variety for the quiver in (f).

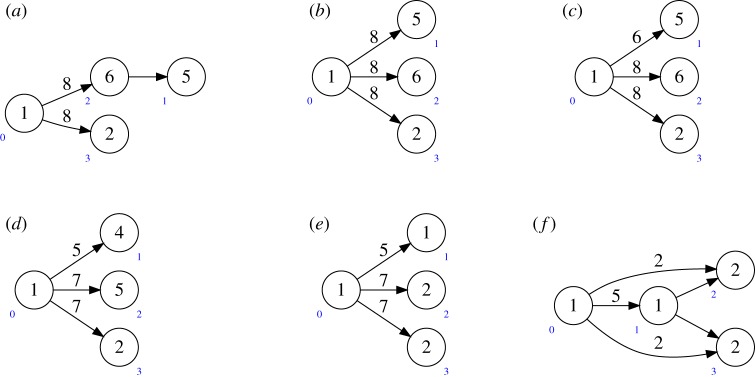

## 5. The ample cone

We now discuss how to compute the ample cone of a quiver flag variety. This is essential if one wants to search systematically for quiver flag zero loci that are Fano. In [1], Craw gives a conjecture that would, in particular, solve this problem, by relating a quiver flag variety *M*(*Q*, **r**) to a toric quiver flag variety. We give a counterexample to this conjecture, and determine the ample cone of *M*(*Q*, **r**) in terms of the combinatorics of the quiver: this is theorem 5.13 below. Our method also involves a toric quiver flag variety: the Abelianization of *M*(*Q*, **r**).

### (a) The multi-graded Plücker embedding

Given a quiver flag variety *M*(*Q*, **r**), Craw (§5 of [1], example 2.9 in [9]) defines a multi-graded analogue of the Plücker embedding:

$$p:M(Q,r)↪M(Q^{\prime },1)\;\mathrm{with}\;1=(1,\ldots ,1).$$

Here *Q*′ is the quiver with the same vertices as *Q* but with the number of arrows *i* → *j*, *i* < *j* given by

$$\dim\left(\frac{\mathrm{Hom}(\det(W_{i}),\det(W_{j}))}{S_{i,j}}\right),$$

where *S*_*i*,*j*_ is spanned by maps which factor through maps to $\det(W_{k})$ with *i* < *k* < *j*. This induces an isomorphism $p^{*}:\mathrm{Pic}(X)⊗R\to \mathrm{Pic}(X)⊗R$ that sends $\det(W_{i}^{\prime })\mapsto \det(W_{i}).$ In [1], it is conjectured that this induces a surjection of Cox rings Cox(*M*(*Q*′, **1**)) → Cox(*M*(*Q*, **r**)). This would give information about the Mori wall and chamber structure of *M*(*Q*, **r**). In particular, by the proof of theorem 2.8 of [13], a surjection of Cox rings together with an isomorphism of Picard groups (which we have here) implies an isomorphism of effective cones.

We provide a counterexample to the conjecture. To do this, we exploit the fact that quiver flag varieties are Mori dream spaces, and so the Mori wall and chamber structure on NE^1^(*M*(*Q*, **r**))⊂Pic(*M*(*Q*, **r**)) coincides with the GIT wall and chamber structure. This gives GIT characterizations for effective divisors, ample divisors, nef divisors, and the walls.

**Theorem 5.1 (Dolgachev & Hu [14])**

*Let* *X* *be a Mori dream space obtained as a GIT quotient of* *G* *acting on* $V=C^{N}$ *with stability condition* $\tau \in \chi (G)=\mathrm{Hom}(G,C^{*}).$ *Identifying Pic*(*X*)≅*χ*(*G*), *we have that*:

— *v*∈*χ*(*G*) *is ample if* *V*^*s*^(*v*) = *V*^*ss*^(*v*) = *V*^*s*^(*τ*).
— *v* *is on a wall if* *V*^*ss*^(*v*)≠*V*^*s*^(*v*).
— *v*∈NE^1^(*X*) *if* $V^{ss}\neq \emptyset .$

When combined with King's characterization [2] of the stable and semistable points for the GIT problem defining *M*(*Q*, **r**), this determines the ample cone of any given quiver flag variety. In theorem 5.13 below we make this effective, characterizing the ample cone in terms of the combinatorics of *Q*. We can also theorem 5.1 to see a counterexample to conjecture 6.4 in [1].

**Example 5.2**

Consider the quiver *Q* and dimension vector **r** as in (a). The target *M*(*Q*′, **1**) of the multi-graded Plücker embedding has the quiver *Q*′ shown in (b).

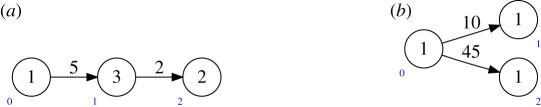

One can see this by noting that $\mathrm{Hom}(\det(W_{2}),\det(W_{1}))=0$, and that after taking ∧^3^ (respectively, ∧^2^) the surjection $O^{⊕5}\to W_{1}\to 0$ (respectively, $O^{⊕10}\to W_{2}\to 0$) becomes

$$O^{⊕10}\to W_{1}\to 0\;(\mathrm{respectively},\;O^{⊕45}\to W_{2}\to 0).$$

In this case, *M*(*Q*′, **1**) is a product of projective spaces and so the effective cone coincides with the nef cone, which is just the closure of the positive orthant. The ample cone of *M*(*Q*, **r**) is indeed the positive orthant, as we will see later. However, here we will find an effective character not in the ample cone. We will use King's characterization (definition 1.1 of [2]) of semi-stable points with respect to a character *χ* of $\prod_{i=0}^{\rho }Gl(r_{i})$: a representation *R* = (*R*_*i*_)_*i*∈*Q*_0__ is semi-stable with respect to *χ* = (*χ*_*i*_)^*ρ*^_*i*=0_ if and only if

— $\sum_{i=0}^{\rho }\chi_{i}\dim_{C}(R_{i})=0$; and
— for any subrepresentation *R*′ of *R*, $\sum_{i=0}^{\rho }\chi_{i}\dim_{C}(R_{i}^{\prime })\geq 0.$

Consider the character *χ* = ( − 1, 3) of *G*, which we lift to a character of $\prod_{i=0}^{\rho }Gl(r_{i})$ by taking *χ* = ( − 3, − 1, 3). We will show that there exists a representation *R* = (*R*_0_, *R*_1_, *R*_2_) which is semi-stable with respect to *χ*. The maps in the representation are given by a triple (*A*, *B*, *C*)∈ *Mat*(3 × 5) × *Mat*(2 × 3) × *Mat*(2 × 3). Suppose that

$$A\text{ has full rank},\;B=\left[\begin{array}{ccc} 1 & 0 & 0\\ 0 & 1 & 0 \end{array}\right]\;\mathrm{and}\;C=\left[\begin{array}{ccc} 0 & 0 & 0\\ 0 & 0 & 1 \end{array}\right],$$

and that *R*′ is a subrepresentation with dimensions *a*, *b*, *c*. We want to show that −3*a* − *b* + 3*c*≥0. If *a* = 1 then *b* = 3, as otherwise the image of *A* is not contained in *R*′_1_. Similarly, this implies that *c* = 2. So suppose that *a* = 0. The maps *B* and *C* have no common kernel, so *b* > 0 implies *c* > 0, and −*b* + 3*c*≥0 as *b* ≤ 3. Therefore, *R* is a semi-stable point for *χ*, and as quiver flag varieties are Mori Dream Spaces, *χ* is in the effective cone.

Therefore, there cannot exist a Mori embedding of *M*(*Q*, *r*) into *M*(*Q*_0_, 1) because it would induce an isomorphism of effective cones.

### (b) Abelianization

We consider now the toric quiver flag variety associated with a given quiver flag variety *M*(*Q*, **r**) which arises from the corresponding Abelian quotient. Let *T*⊂*G* be the diagonal maximal torus. Then the action of *G* on Rep(*Q*, **r**) induces an action of *T* on Rep(*Q*, **r**), and the inclusion *i*:*χ*(*G*)↪*χ*(*T*) allows us to interpret the special character *θ* as a stability condition for the action of *T* on Rep(*Q*, **r**). The Abelian quotient is then Rep(*Q*, **r**)//_*i*(*θ*)_*T*. Let us see that Rep(*Q*, **r**)//_*θ*_*T* is a toric quiver flag variety. Let *λ* = (*λ*_1_, …, *λ*_*ρ*_) denote an element of $T=\prod_{i=1}^{\rho }{\left(C^{*}\right)}^{r_{i}}$, where *λ*_*j*_ = (*λ*_*j*1_, …, *λ*_*jr*_*j*__). Let (*w*_*a*_)_*a*∈*Q*_1__∈Rep(*Q*, **r**). Here *w*_*a*_ is an *r*_*t*(*a*)_ × *r*_*s*(*a*)_ matrix. The action of *λ* on (*w*_*a*_)_*a*∈*Q*_1__ is defined by

$$w_{a}(i,j)\mapsto \lambda_{s(a)i}^{-1}w_{a}(i,j)\lambda_{t(a)j}.$$

Hence this is the same as the group action on the quiver *Q*^ab^ with vertices

$$Q_{0}^{\mathrm{ab}}=\{v_{ij}:0\leq i\leq \rho ,1\leq j\leq r_{i}\},$$

and the number of arrows between *v*_*ij*_ and *v*_*kl*_ is the number of arrows in the original quiver between vertices *i* and *k*. Clearly, *i*(*θ*)∈*χ*(*T*) is the character prescribed by §2a. Hence

$$\mathrm{Rep}(Q,r)/{/}_{\theta }T=M(Q^{\mathrm{ab}},1).$$

We call *Q*^ab^ the *Abelianized* quiver.

**Example 5.3**

Let *Q* be the quiver

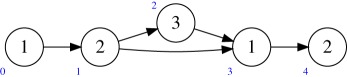

Then *Q*^ab^ is

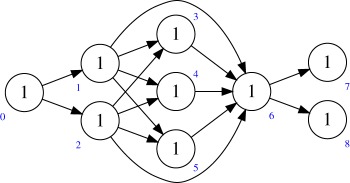

Martin [15] has studied the relationship between the cohomology of Abelian and non-Abelian quotients. We state his result specialized to quiver flag varieties, then extend this to a comparison of the ample cones. To simplify notation, denote *M*_*Q*_ = *M*(*Q*, **r**), *M*_*Q*^ab^_ = *M*(*Q*^ab^, (1, …, 1)) and *V* = Rep(*Q*, **r**) = Rep(*Q*^ab^, (1, …, 1)). For *v*∈*χ*(*G*), let *V*^*s*^_*v*_(*T*) denote the *T*-stable points of *V* and *V*^*s*^_*v*_(*G*) denote the *G*-stable points, dropping the subscript if it is clear from context. It is easy to see that *V*^*s*^(*G*)⊂*V*^*s*^(*T*). The Weyl group *W* of (*G*, *T*) is $\prod_{i=1}^{\rho }S_{r_{i}},$ where *S*_*r*_*i*__ is the symmetric group on *r*_*i*_ letters. Let *π*:*V*^*s*^(*G*)/*T* → *V*^*s*^(*G*)/*G* be the projection. The Weyl group acts on the cohomology of *M*(*Q*^ab^, **1**), and also on the Picard group, by permuting the *W*_*v*_*i*1__, …, *W*_*v*_*ir*_*i*___. It is well-known (e.g. Atiyah–Bott [16]) that

$$\pi^{*}:H^{*}{\left(\frac{V^{s}(G)}{T}\right)}^{W}≅H^{*}(M_{Q}).$$

**Theorem 5.4 (Martin [15])**

*There is a graded surjective ring homomorphism*

$$\phi :H^{*}{\left(M_{Q^{\mathrm{ab}}},C\right)}^{W}\to H^{*}\left(\frac{V^{s}(G)}{T},C\right)\overset{\pi^{*}}{\to }H^{*}(M_{Q},C),$$

*where the first map is given by the restriction* *V*^*s*^(*T*)/*T* → *V*^*s*^(*G*)/*T*. *The kernel is the annihilator of* $e=\prod_{i=1}^{\rho }\prod_{1\leq j,k\leq r_{i}}c_{1}(W_{v_{ij}}^{*}⊗W_{v_{ik}})$.

**Remark 5.5**

This means that any class *σ*∈H*(*M*_*Q*_) can be lifted (non-uniquely) to a class $\tilde{\sigma }\in H^{*}(M_{Q^{\mathrm{ab}}})$. Moreover, $e\cap \tilde{\sigma }$ is uniquely determined by *σ*.

**Corollary 5.6**

*Let* *E* *be a representation of* *G* *defining representation theoretic bundles* *E*_*G*_ → *M*_*Q*_ and *E*_*T*_ → *M*_*Q*^ab^_. *Then* *ϕ*(*c*_*i*_(*E*_*T*_)) = *c*_*i*_(*E*_*G*_).

**Proof.**

Recall that

$$\begin{array}{rl} E_{G} & =\frac{\left(V^{s}(G)\times E\right)}{G}\to M_{Q}\\ \mathrm{and}\;\;E_{T} & =\frac{\left(V^{s}(T)\times E\right)}{T}\to M_{Q^{\mathrm{ab}}}. \end{array}$$

Define

$$E_{G}^{\prime }=\frac{\left(V^{s}(G)\times E\right)}{T}\to \frac{V^{s}(G)}{T}.$$

Let *f* be the inclusion *V*^*s*^(*G*)/*T* → *V*^*s*^(*T*)/*T*. Clearly, f*(*E*_*T*_) = *E*_*G*_′ as *E*_*G*_′ is just the restriction of *E*_*T*_. Considering the square

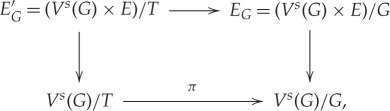

we see that π*(*E*_*G*_) = *E*_*G*_′. Then we have that f*(*E*_*T*_) = π*(*E*_*G*_), and so in particular f*(*c*_*i*_(*E*_*T*_)) = π*(*c*_*i*_(*E*_*G*_)). The result now follows from Martin's theorem (theorem 5.4). ▪

**Remark 5.7**

Note that *E*_*T*_ always splits as a direct sum of line bundles on *M*(*Q*^*ab*^, (1, …, 1)), as any representation of *T* splits into rank one representations. In particular, this means that if (*Q*, *E*_*G*_) defines a quiver flag zero locus, (*Q*^*ab*^, *E*_*T*_) defines a quiver flag zero locus which is also a toric complete intersection.

The corollary shows that in degree 2, the inverse of Martin's map is

$$i:c_{1}(W_{i})\mapsto \sum_{j=1}^{r_{i}}c_{1}(W_{v_{ij}}).$$

In particular, using (2.1), we have that *i*(*ω*_*M*_*Q*__) = *ω*_*M*_*Q*^ab^__, where *ω*_*X*_ is the canonical bundle of *X*.

**Proposition 5.8**

*Let* Amp(*Q*), Amp(*Q*^ab^) *denote the ample cones of* *M*_*Q*_ *and* *M*_*Q*^ab^_, *respectively. Then*

$$i(\mathrm{Amp}(Q))=\mathrm{Amp}{\left(Q^{\mathrm{ab}}\right)}^{W}.$$

**Proof.**

Let *v* be a character for *G*, denoting its image under *i*:*χ*(*G*)↪*χ*(*T*) as *v* as well. The image of *i* is *W*-invariant, and in fact *i*(*χ*(*G*)) = *χ*(*T*)^*W*^ (this reflects that *W*-invariant lifts of divisors are unique).

Note that *V*^*ss*^_*v*_(*G*)⊂*V*^*ss*^_*v*_(*T*). To see this, suppose *v*∈*V* is semi-stable for *G*. Let $\lambda :C^{*}\to T$ be a one-parameter subgroup of *T* such that lim_*t* → 0_*λ*(*t*) · *v* exists. By inclusion, *λ* is a one-parameter subgroup of *G*, and so 〈*v*, *λ*〉≥0 by semi-stability of *v*. Hence *v*∈*V*^*ss*^_*v*_(*T*). It follows that, if *v*∈NE^1^(*M*_*Q*_), then $V_{v}^{ss}(G)\neq \emptyset$, so $V_{v}^{ss}(T)\neq \emptyset$, and hence by theorem 5.1 *v*∈NE^1^(*M*_*Q*^ab^_)^*W*^.

Ciocan–Fontanine–Kim–Sabbah use duality to construct a projection [17]

$$p:{\mathrm{NE}}_{1}(M_{Q^{\mathrm{ab}}})\to {\mathrm{NE}}_{1}(M_{Q}).$$

Suppose that *α*∈Amp(*Q*). Then for any *C*∈NE_1_(*M*_*Q*^ab^_), *i*(*α*) · *C* = *α* · *p*(*C*) > 0. So *i*(*α*)∈Amp(*Q*^ab^)^*W*^.

Let Wall(*G*)⊂Pic(*M*_*Q*_) denote the union of all GIT walls given by the *G* action, and similarly for Wall(*T*). Recall that *v*∈Wall(*G*) if and only if it has a non-empty strictly semi-stable locus. Suppose *v*∈Wall(*G*), with *v* in the strictly semi-stable locus. That is, there exists a non-trivial $\lambda :C^{*}\to G$ such that lim_*t* → 0_*λ*(*t*) · *v* exists and 〈*v*, *λ*〉 = 0. Now we do not necessarily have Im(*λ*)⊂*T*, but the image is in some maximal torus, and hence there exists *g*∈*G* such that Im(*λ*)⊂*g*^−1^*Tg*. Consider *λ*′ = *gλg*^−1^. Then $\lambda^{\prime }(C^{*})\subset T.$ Since *g* · *v* is in the orbit of *v* under *G*, it is semi-stable with respect to *G*, and hence with respect to *T*. In fact, it is strictly semi-stable with respect to *T*, since lim_*t* → 0_*λ*′(*t*)*g* · *v* = lim_*t* → 0_*gλ*(*t*) · *v* exists, and 〈*v*, *λ*′〉 = 〈*v*, *λ*〉 = 0. So as a character of *T*, *v* has a non-empty strictly semi-stable locus, and we have shown that

$$i(\mathrm{Wall}(G))\subset \mathrm{Wall}{\left(T\right)}^{W}.$$

This means that the boundary of *i*(Amp(*Q*)) has empty intersection with Amp(*Q*^ab^)^*W*^. Since both are full dimensional cones in the *W* invariant subspace, the inclusion *i*(Amp(*Q*))⊂Amp(*Q*^ab^)^*W*^ is in fact an equality. ▪

**Example 5.9**

Consider again the example

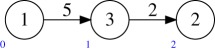

The Abelianization of this quiver is

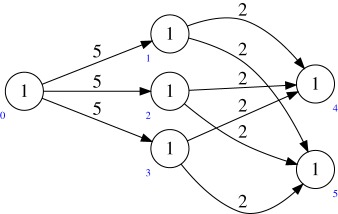

Walls are generated by collections of divisors that generate cones of codimension 1. We then intersect them with the Weyl invariant subspace, generated by (1, 1, 1, 0, 0) and (0, 0, 0, 1, 1). In this subspace, the walls are generated by

(1,1,1,0,0),(0,0,0,1,1),(−2,−2,−2,3,3).

Combined with example 5.2, this determines the wall-and-chamber structure of the effective cone of *M*(*Q*, **r**). That is, it has three walls, each generated by one of *v*_1_: = (1, 0), *v*_2_: = ( − 2, 3) and *v*_3_ = (0, 1). There are two cones generated by (*v*_1_, *v*_3_) and (*v*_2_, *v*_3_), respectively.

### (c) The toric case

As a prelude to determining the ample cone of a general quiver flag variety, we first consider the toric case. Recall that a smooth projective toric variety (or orbifold) can be obtained as a GIT quotient of $C^{N}$ by an *ρ*-dimensional torus.

**Definition 5.10**

The *GIT data* for a toric variety is an *ρ*-dimensional torus *K* with cocharacter lattice $L=\mathrm{Hom}(C^{*},K)$, and *N* characters *D*_1_, …, *D*_*N*_∈*L*^∨^, together with a stability condition $w\in L^{\vee }⊗R$.

These linear data give a toric variety (or Deligne–Mumford stack) as the quotient of an open subset $U_{w}\subset C^{N}$ by *K*, where *K* acts on $C^{N}$ via the map $K\to {\left(C^{*}\right)}^{N}$ defined by the *D*_*i*_. *U*_*w*_ is defined as

$$\left\{(z_{1},\ldots ,z_{N})\in C^{N}\;|\;w\in \mathrm{Cone}(D_{i}:z_{i}\neq 0)\right\},$$

that is, its elements can have zeroes at *z*_*i*_, *i*∈*I*, only if *w* is in the cone generated by *D*_*i*_, *i*∉*I*. Assume that all cones given by subsets of the divisors that contain *w* are full dimensional, as is the case for toric quiver flag varieties. Then the ample cone is the intersection of all of these.

In [18], the GIT data for a toric quiver flag variety is detailed; we present it slightly differently. The torus is $K={\left(C^{*}\right)}^{\rho }$. Let *e*_1_, …, *e*_*ρ*_ be standard basis of $L^{\vee }=Z^{\rho }$ and set *e*_0_: = 0. Then each *a*∈*Q*_1_ gives a weight *D*_*a*_ = − *e*_*s*(*a*)_ + *e*_*t*(*a*)_. The stability condition is **1** = (1, 1, …, 1). Identify *L*^∨^≅Pic*M*(*Q*, **1**). Then *D*_*a*_ = *W*_*a*_: = W*_s(*a*)_⊗*W*_*t*(*a*)_.

A minimal generating set for a full dimensional cone for a toric quiver flag variety is given by *ρ* linearly independent *D*_*a*_*i*__, *a*_*i*_∈*Q*_1_. Therefore, for each vertex *i* with 1 ≤ *i* ≤ *ρ*, we need an arrow *a*_*i*_ with either *s*(*a*) = *i* or *t*(*a*) = *i*, and these arrows should be distinct. For the positive span of these divisors to contain **1** requires that *D*_*a*_*i*__ has *t*(*a*_*i*_) = *i*. Fix such a set *S* = {*a*_1_, …, *a*_*ρ*_}, and denote the corresponding cone by *C*_*S*_. As mentioned, the ample cone is the intersection of such cones *C*_*S*_. The set *S* determines a path from 0 to *i* for each *i*, given by concatenating (backwards) *a*_*i*_ with *a*_*s*(*a*_*i*_)_ and so on; let us write *f*_*ij*_ = 1 if *a*_*j*_ is in the path from 0 to *i*, and 0 otherwise. Then

$$e_{i}=\sum_{j=1}^{\rho }f_{ij}D_{a_{j}}.$$

This gives us a straightforward way to compute the cone *C*_*S*_. Let *B*_*S*_ be the matrix with columns given by the *D*_*a*_*i*__, and let *A*_*S*_ = *B*^−1^_*S*_. The columns of *A*_*S*_ are given by the aforementioned paths: the *j*th column of *A*_*S*_ is $\sum_{i=1}^{\rho }f_{ij}e_{i}$. If *c*∈Amp(*Q*), then *A*_*S*_*c*∈*A*_*S*_(Amp(*Q*))⊂*A*_*S*_(*C*_*S*_). Since *A*_*S*_*D*_*a*_*i*__ = *e*_*i*_, this means that *A*_*S*_*c* is in the positive orthant.

**Proposition 5.11**

*Let* *M*(*Q*, **1**) *be a toric quiver flag variety. Let* *c*∈Amp(*Q*), *c* = (*c*_1_, …, *c*_*ρ*_), *be an ample class, and suppose that vertex* *i* *of the quiver* *Q* *satisfies the following condition: for all* *j*∈*Q*_0_ *such that* *j* > *i*, *there is a path from* 0 *to* *j* *not passing through* *i*. *Then* *c*_*i*_ > 0.

**Proof.**

Choose a collection *S* of arrows *a*_*j*_∈*Q*_1_ such that the span of the associated divisors *D*_*a*_*j*__ contains the stability condition **1**, and such that the associated path from 0 to *j* for any *j* > *i* does not pass through *i*. Then the (*i*, *i*) entry of *A*_*S*_ is 1 and all other entries of the *i*^*th*^ row are zero. As *A*_*S*_*c* is in the positive orthant, *c*_*i*_ > 0. ▪

**Corollary 5.12**

*Let* *M*(*Q*, **r**) *be a quiver flag variety, not necessarily toric. If* *c* = (*c*_1_, …, *c*_*ρ*_)∈Amp(*Q*) *and* *r*_*j*_ > 1, *then* *c*_*j*_ > 0.

**Proof.**

Consider the Abelianized quiver. For any vertex *v*∈*Q*^ab^_0_, we can always choose a path from the origin to *v* that does not pass through *v*_*j*1_: if there is an arrow between *v*_*j*1_ and *v*, then there is an arrow between *v*_*j*2_ and *v*, so any path through *v*_*j*1_ can be rerouted through *v*_*j*2_. Then we obtain that the *j*1 entry of *i*(*c*) is positive-but this is just *c*_*j*_. ▪

### (d) The ample cone of a quiver flag variety

Let *M*(*Q*, **r**) be a quiver flag variety and *Q*′ be the associated Abelianized quiver. Here paths are defined to be directed paths consisting of at least one arrow. A path passes through a vertex *i* if either the source or the target of one of the arrows in the path is *i*. For each *i*∈{1, …, *ρ*}, define

$$T_{i}:=\{j\in Q_{0}|\mathrm{all}\;\mathrm{paths}\;\mathrm{from}\;\mathrm{the}\;\mathrm{source}\;\mathrm{to}\;v_{j1}\;\mathrm{pass}\;\mathrm{through}\;v_{i1}\;\mathrm{in}\;\mathrm{the}\;\mathrm{abelianized}\;\mathrm{quiver}\}.$$

Note that *i*∈*T*_*i*_, as every path from 0 to *v*_*i*1_ passes through *v*_*i*1_ by definition. There are no paths from the source to the source, which is therefore not in *T*_*i*_ for any *i*. If *r*_*i*_ > 1 then *T*_*i*_ = {*i*}.

**Theorem 5.13**

*The nef cone of* *M*(*Q*, **r**) *is given by the following inequalities. Suppose that* *a* = (*a*_1_, …, *a*_*ρ*_)∈Pic(*M*_*Q*_). *Then* *a* *is nef if and only if*

5.1 $$\sum_{j\in T_{i}}r_{j}a_{j}\geq 0\;i=1,2,\ldots ,\rho .$$

**Proof.**

We have already shown that the Weyl invariant part of the nef cone of *M*_*Q*′_: = *M*(*Q*′, **1**) is the image of the nef cone of *M*_*Q*_: = *M*(*Q*, **r**) under the natural map *π*:Pic(*M*_*Q*_) → Pic(*M*_*Q*′_). Label the vertices of *Q*′ as *v*_*ij*_, *i*∈{0, …, *ρ*}, *j*∈{1, …, *r*_*i*_}, and index elements of Pic(*M*_*Q*′_) as (*b*_*ij*_). The inequalities defining the ample cone of *M*_*Q*′_ are given by a choice of arrow *A*_*ij*_∈*Q*′_1_, *t*(*A*_*ij*_) = *v*_*ij*_ for each *v*_*ij*_. This determines a path *P*_*ij*_ from 0 → *v*_*ij*_ for each vertex *v*_*ij*_. For each *v*_*ij*_ the associated inequality is

5.2 $$\sum_{v_{ij}\in P_{kl}}b_{kl}\geq 0.$$

Suppose that *a* is nef. We want to show that *a* satisfies the inequalities (5.1). We do this by finding a collection of arrows such that the inequality (5.2) applied to *π*(*a*) is just the inequality (5.1).

It suffices to do this for *i* such that *r*_*i*_ = 1 (as we have already shown that the inequalities are the same in the *r*_*i*_ > 1 case). Choose a set of arrows such that the associated paths avoid *v*_*i*1_ if possible: in other words, if *v*_*i*1_∈*P*_*kl*_, then assume *k*∈*T*_*i*_. Notice that if *v*_*i*1_∈*P*_*kl*_1__, then *v*_*i*1_∈*P*_*kl*_2__. By assumption *π*(*a*) satisfies the *i*^*th*^ inequality associated with this collection of arrows, that is

$$\sum_{k\in T_{i}}r_{k}a_{k}=\sum_{v_{i1}\in P_{kl}}a_{k}\geq 0.$$

Therefore, if *C* is the cone defined by (5.1), we have shown that Nef(*M*_*Q*_)⊂*C*.

Suppose now that *a*∈*C* and take a choice of arrows *A*_*kl*_. Write *π*(*a*) = (*a*_*ij*_). We prove that the inequalities 5.2 are satisfied starting at *v*_*ρr*_*ρ*__. For *ρ*, the inequality is *a*_*ρr*_*ρ*__≥0, which is certainly satisfied. Suppose the (*ij* + 1), (*ij* + 2), …, (*ρr*_*ρ*_) inequalities are satisfied. The inequality we want to establish for (*ij*) is

$$\sum_{v_{i1}\in P_{kl}}a_{kl}=a_{ij}+\sum_{k\in T_{i}-\{i\}}\sum_{l=1}^{r_{l}}a_{kl}+\Gamma =a_{i}+\sum_{k\in T_{i}-\{i\}}r_{k}a_{k}+\Gamma \geq 0,$$

where

$$\Gamma =\sum_{s(A_{kl})=v_{ij},k\notin T_{i}}\left(a_{kl}+\sum_{v_{kl}\in P_{st}}a_{st}\right).$$

This uses the fact that for *k*∈*T*_*i*_, *v*_*i*1_∈*P*_*kl*_ for all *l*, and that if *k*∉*T*_*i*_, and *v*_*kl*_∈*P*_*st*_, we also have that *s*∉*T*_*i*_.

As *a*∈*C* it suffices to show that *Γ*≥0. By the induction hypothesis $a_{kl}+\sum_{v_{kl}\in P_{st}}a_{st}\geq 0$, and therefore *Γ*≥0. This shows that *π*(*a*) satisfies (5.2). ▪

### (e) Nef line bundles are globally generated

We conclude this section by proving that nef line bundles on quiver flag varieties are globally generated. Craw [1] has shown that the nef line bundles $\det(W_{i})$ on *M*(*Q*, **r**) are globally generated; they span a top-dimensional cone contained in the nef cone (and thus all line bundles in this cone are globally generated). Nef line bundles on toric varieties are known to be globally generated. This result for quiver flag varieties will be important for us because in order to use the Abelian/non-Abelian correspondence to compute the quantum periods of quiver flag zero loci, we need to know that the bundles involved are convex. Convexity is a difficult condition to understand geometrically, but it is implied by global generation.

To prove the proposition, we will need the following lemma about the structure of the *T*_*i*_.

**Lemma 5.14**

*The set* {*T*_*i*_:*i*∈{1, …, *ρ*}} *has a partial order given by*

$$T_{i}\leq T_{j}\Leftrightarrow T_{j}\subset T_{i},$$

*such that for all* *j*, *the set* {*T*_*i*_ ≤ *T*_*j*_} *is a chain*.

**Proof.**

Observe that if *i*∈*T*_*j*_∩*T*_*k*_ for *j* < *k*, then *T*_*k*_⊂*T*_*j*_: if all paths from 0 to *i*1 pass through both *j*1 and *k*1, then all paths from 0 to *k*1 must pass through *j*1. So *k*∈*T*_*j*_ and hence *T*_*k*_⊂*T*_*j*_. Therefore, if *T*_*j*_ ≤ *T*_*i*_ and *T*_*k*_ ≤ *T*_*i*_ for *j* < *k*, then *i*∈*T*_*j*_∩*T*_*k*_, and so *T*_*j*_ ≤ *T*_*k*_. Hence {*T*_*k*_|*T*_*k*_ ≤ *T*_*j*_} is totally ordered for all *j*. ▪

**Proposition 5.15**

*Let* *L* *be a nef line bundle on* *M*(*Q*, *r*). *Then* *L* *is globally generated*.

**Proof.**

Let *M*: = {*T*_*i*_|*T*_*i*_is minimal}. By the lemma, $\{1,\ldots ,\rho \}=\underset{T_{i}\in M}{⨆}T_{i}.$ Suppose *L* is given by the character (*a*_1_, …, *a*_*ρ*_). Write *L* as *L* = ⊗_*T*_*i*_∈*M*_*L*_*T*_*i*__, where each *L*_*T*_*i*__ comes from a character (*b*_1_, …, *b*_*ρ*_)∈*χ*(*G*) satisfying *b*_*j*_ = 0 if *j*∉*T*_*i*_.

*L* is nef if and only if all the *L*_*T*_*i*__, *T*_*i*_∈*M* are nef. To see this, note that for each *j* the inequality

$$\sum_{k\in T_{j}}r_{k}a_{k}\geq 0,$$

involves terms from a minimal *T*_*i*_ if and only if *j*∈*T*_*i*_, in which case it involves only terms from *T*_*i*_. It therefore suffices to show the statement of the proposition for each *L*_*j*_. Therefore suppose that {*j*|*a*_*j*_≠0}⊂*T*_*i*_ for *T*_*i*_ minimal. If *r*_*i*_ > 1, then *T*_*i*_ = {*i*}, so $L=\det{\left(W_{i}\right)}^{⊗a_{i}}$ which is globally generated. So we further assume that *r*_*i*_ = 1. For *k*∈*T*_*i*_, *k* > *i*, define *h*′(*k*) such that *T*_*h*′(*k*)_ is the maximal element such that *T*_*i*_ ≤ *T*_*h*′(*k*)_ < *T*_*k*_. This is well defined because the set {*T*_*j*_|*T*_*j*_ < *T*_*k*_} is a chain.

A section of *L* is a *G*-equivariant section of the trivial line bundle on Rep(*Q*, **r**), where the action of *G* on the line bundle is given by the character $\prod \chi_{i}^{a_{i}}.$ A point of Rep(*Q*, **r**) is given by ${\left(\phi_{a}\right)}_{a\in Q_{1}},\phi_{a}:C^{r_{s(a)}}\to C^{r_{t(a)}},$ where *G* acts by change of basis. A choice of path *i* → *j* on the quiver gives an equivariant map $\mathrm{Rep}(Q,r)\to \mathrm{Hom}(C^{r_{i}},C^{r_{j}})$ where *G* acts on the image by *g* · *ϕ* = *g*_*j*_*ϕg*^−1^_*i*_. If *r*_*i*_ = *r*_*j*_ = 1, such maps can be composed.

For *j*∈*T*_*i*_, define *f*_*j*_ as follows:

— If *j* = *i*, let *f*_*i*_ be any homogeneous polynomial of degree $d_{i}=\sum_{k\in T_{i}}r_{k}a_{k}\geq 0$ in the maps given by paths 0 → *i*. Therefore, *f*_*i*_ is a section of the line bundle given by the character *χ*^*d*_*i*_^_*i*_.
— If *j* > *i*, *r*_*j*_ = 1, let *f*_*j*_ be any homogeneous polynomial of degree $d_{j}=\sum_{k\in T_{j}}r_{k}a_{k}\geq 0$ in the maps given by paths *h*′(*j*) → *j*. Note that *r*_*h*′(*j*)_ = 1 as by construction *j*, *h*′(*j*)∈*T*_*h*′(*j*)_. So *f*_*j*_ defines a section of the line bundle given by character *χ*^−*d*_*j*_^_*h*(*j*)_*χ*^*d*_*j*_^_*j*_.
— If *j* > *i*, *r*_*j*_ > 1, let *f*_*j*_ be a homogeneous polynomial of degree *a*_*k*_≥0 in the minors of the matrix whose columns are given by the paths *h*′(*j*) → *j*. That is, *f*_*j*_ is a section of the line bundle given by character *χ*^−*r*_*j*_*a*_*j*_^_*h*′(*j*)_*χ*^*a*_*j*_^_*j*_.

For any *x*∈Rep(*Q*, **r**) which is semi-stable, and for any *j*∈*T*_*i*_, there exists an *f*_*j*_ as above with *f*_*j*_(*x*)≠0, because *j*∈*T*_*h*′(*j*)_. Fixing *x*, choose such *f*_*j*_. Define $\sigma :=\prod_{j\in T_{i}}f_{j}:\mathrm{Rep}(Q,r)\to C$. Then *σ* defines a section of the line bundle given by character

$$\prod_{j\in T_{i}}\chi_{j}^{b_{j}}=\chi_{i}^{d_{i}}\cdot \prod_{j\in T_{i},j\neq i,r_{j}=1}\chi_{h^{\prime }(j)}^{-d_{j}}\chi_{j}^{d_{j}}\cdot \prod_{j\in T_{i},j\neq i,r_{j}>1}\chi_{h^{\prime }(j)}^{-r_{j}a_{j}}\chi_{j}^{a_{j}}.$$

We need to check that *b*_*l*_ = *a*_*l*_ for all *l*. This is obvious for *l*∈*T*_*i*_ with *r*_*l*_ > 1. For *r*_*l*_ = 1,

$$b_{l}=\sum_{j\in T_{l}}r_{j}a_{j}-\sum_{k\in T_{l}-\{l\},h^{\prime }(k)=l}\sum_{j\in T_{k}}r_{j}a_{j}.$$

This simplifies to *a*_*l*_ because *T*_*l*_ − {*l*} = ⊔_*j*∈*T*_*l*_,*h*′(*j*) = *l*_*T*_*j*_. Therefore, *σ* gives a well-defined non-vanishing section of *L*, so *L* is globally generated. ▪

## 6. The Abelian/non-Abelian correspondence

The main theoretical result of this paper, theorem 6.4 below, proves the Abelian/non-Abelian correspondence with bundles [17, conjecture 6.1.1] for quiver flag zero loci. This determines all genus-zero Gromov–Witten invariants, and hence the quantum cohomology, of quiver flag varieties, as well as all genus-zero Gromov–Witten invariants of quiver flag zero loci involving cohomology classes that come from the ambient space. In particular, it determines the *quantum period* (see definition 6.1) of a quiver flag varieties or quiver flag zero locus *X* with *c*_1_(*T*_*X*_)≥0.

### (a) A brief review of Gromov–Witten theory

We give a very brief review of Gromov–Witten theory, mainly to fix notation, see [3,17] for more details and references. Let *Y* be a smooth projective variety. Given $n\in Z_{\geq 0}$ and *β*∈*H*_2_(*Y* ), let *M*_0,*n*_(*Y*, *β*) be the moduli space of genus zero stable maps to *Y* of class *β*, and with *n* marked points [19]. While this space may be highly singular and have components of different dimensions, it has a *virtual fundamental class* [*M*_0,*n*_(*Y*, *β*)]^*virt*^ of the expected dimension [20,21]. There are natural evaluation maps *ev*_*i*_:*M*_0,*n*_(*Y*, *β*) → *Y* taking a class of a stable map *f*:*C* → *Y* to *f*(*x*_*i*_), where *x*_*i*_∈*C* is the *i*th marked point. There is also a line bundle *L*_*i*_ → *M*_0,*n*_(*Y*, *β*) whose fibre at *f*:*C* → *Y* is the cotangent space to *C* at *x*_*i*_. The first Chern class of this line bundle is denoted *ψ*_*i*_. Define

6.1 $${\left\langle \tau_{a_{1}}(\alpha_{1}),\ldots ,\tau_{a_{n}}(\alpha_{n})\right\rangle }_{n,\beta }=\int_{{\left[M_{0,n}(Y,\beta )\right]}^{virt}}\prod_{i=1}^{n}ev_{i}^{*}(\alpha_{i})\psi_{i}^{a_{i}},$$

where the integral on the right-hand side denotes cap product with the virtual fundamental class. If *a*_*i*_ = 0 for all *i*, this is called a (genus zero) Gromov–Witten invariant and the *τ* notation is omitted; otherwise it is called a descendant invariant. It is deformation invariant.

We consider a generating function for descendant invariants called the *J-function*. Write *q*^*β*^ for the element of $Q[H_{2}(Y)]$ representing *β*∈*H*_2_(*Y* ). Write *N*(*Y* ) for the Novikov ring of *Y* :

$$N(Y)=\left\{\sum_{\beta \in {\mathrm{NE}}_{1}(Y)}c_{\beta }q^{\beta }|\begin{array}{l} c_{\beta }\in C,\;\mathrm{for}\;\mathrm{each}\;d\geq 0\;\mathrm{there}\;\mathrm{are}\;\mathrm{only}\;\mathrm{finitely}\;\mathrm{many}\\ \beta \;\mathrm{such that}\;\omega \cdot \beta \leq d\;\mathrm{and}\;c_{\beta }\neq 0 \end{array}\right\}.$$

Here *ω* is the Kähler class on *Y* . The J-function assigns an element of H*(*Y* )⊗*N*(*Y* )[[*z*^−1^]] to every element of H*(*Y* ), as follows. Let *ϕ*_1_, …, *ϕ*_*N*_ be a homogeneous basis of H*(*Y* ), and let *ϕ*^1^, …, *ϕ*^*N*^ be the Poincaré dual basis. Then the J-function is given by

6.2 $$J_{X}(\tau ,z):=1+\tau z^{-1}+z^{-1}\sum_{i}\langle \langle \phi_{i}/(z-\psi )\rangle \rangle \phi^{i}.$$

Here, 1 is the unit class in *H*^0^(*Y* ), *τ*∈H*(*Y* ), and

6.3 $$\langle \langle \phi_{i}/(z-\psi )\rangle \rangle =\sum_{\beta \in {\mathrm{NE}}_{1}(Y)}q^{\beta }\sum_{n=0}^{\infty }\sum_{a=0}^{\infty }\frac{1}{n!z^{a+1}}{\left\langle \tau_{a}(\phi_{i}),\tau ,\ldots ,\tau \right\rangle }_{n+1,\beta }.$$

The *small* J-function is the restriction of the J-function to *H*^0^(*Y* )⊕*H*^2^(*Y* ); closed forms for the small J-function of toric complete intersections and toric varieties are known [22].

**Definition 6.1**

*The quantum period* *G*_*Y*_(*t*) *is the component of* *J*(0) *along* 1∈*H*^•^(*Y*) *after the substitutions* *z*↦1, *q*^*β*^↦*t*^〈−*K*_*Y*_,*β*〉^. *This is a power series in* *t*.

The quantum period satisfies an important differential equation called the quantum differential equation.

A vector bundle *E* → *Y* is defined to be convex if for every genus 0 stable map *f*:*C* → *Y* , *H*^1^(*C*, f*E) = 0. Globally generated vector bundles are convex. Let *X*⊂*Y* be the zero locus of a generic section of a convex vector bundle *E* → *Y* and let **e** denote the total Chern class, which evaluates on a vector bundle *F* of rank *r* as

6.4 $$e(F)=\lambda^{r}+\lambda^{r-1}c_{1}(F)+\cdots +\lambda c_{r-1}(F)+c_{r}(F).$$

The notation here indicates that one can consider **e**(*F*) as the $C^{*}$-equivariant Euler class of *F*, with respect to the canonical action of $C^{*}$ on *F* which is trivial on the base of *F* and scales all fibres. In this interpretation, $\lambda \in H_{C^{*}}^{∙}(pt)$ is the equivariant parameter. The twisted J-function *J*_**e**, *E*_ is defined exactly as the J-function (6.2), but replacing the virtual fundamental class which occurs there (via equations (6.3) and (6.1)) by [*M*_0,*n*_(*Y*, *β*)]^*virt*^∩**e**(*E*_0,*n*,*β*_), where *E*_0,*n*,*β*_ is π_*_(ev*_n+1_(*E*)), *π*:*M*_0,*n*+1_(*Y*, *β*) → *M*_0,*n*_(*Y*, *β*) is the universal curve, and *ev*_*n*+1_:*M*_0,*n*+1_(*Y*, *β*) → *Y* is the evaluation map. *E*_0,*n*,*β*_ is a vector bundle over *M*_0,*n*_(*Y*, *β*), because *E* is convex. Functoriality for the virtual fundamental class [23] implies that

$$j^{*}J_{e,E}(\tau ,z){|}_{\lambda =0}=J_{X}(j^{*}\tau ,z),$$

where *j*:*X* → *Y* is the embedding [24, Theorem 1.1]. Thus one can compute the quantum period of *X* from the twisted J-function. We will use this to compute the quantum period of Fano fourfolds which are quiver flag zero loci.

The Abelian/non-Abelian correspondence is a conjecture [17] relating the J-functions (and more broadly, the quantum cohomology Frobenius manifolds) of GIT quotients *V*//*G* and *V*//*T*, where *T*⊂*G* is the maximal torus. It also extends to considering zero loci of representation theoretic bundles, by relating the associated twisted J-functions. As the Abelianization *V*//*T* of a quiver flag variety *V*//*G* is a toric quiver flag variety, the Abelian/non-Abelian correspondence conjectures a closed form for the J-functions of Fano quiver flag zero loci. Ciocan-Fontanine–Kim–Sabbah proved the Abelian/non-Abelian correspondence (with bundles) when *V*//*G* is a flag manifold [17]. We will build on this to prove the conjectures when *V*//*G* is a quiver flag variety.

### (b) The I-function

We give the J-function in the way usual in the literature: first, by defining a cohomology-valued hypergeometric function called the I-function (which should be understood as a mirror object, but we omit this perspective here), then relating the J-function to the I-function. We follow the construction given by Ciocan-Fontanine *et al*. [17] in our special case. Let *X* be a quiver flag zero locus given by (*Q*, *E*_*G*_) (where we assume *E*_*G*_ is globally generated), and write *M*_*Q*_ = *M*(*Q*, **r**) for the ambient quiver flag variety. Let (*Q*^ab^, *E*_*T*_) be the associated Abelianized quiver and bundle, *M*_*Q*^ab^_ = *M*(*Q*^ab^, (1, …, 1)). Assume, moreover, that *E*_*T*_ splits into nef line bundles; this implies that *E*_*T*_ is convex. To define the I-function, we need to relate the Novikov rings of *M*_*Q*_ and *M*_*Q*^ab^_. Let Pic*Q* (respectively Pic*Q*^ab^) denote the Picard group of *M*_*Q*_ (respectively of *M*_*Q*^ab^_), and similarly for the cones of effective curves and effective divisors. The isomorphism Pic*Q* → (Pic*Q*^ab^)^*W*^ gives a projection *p*:NE_1_(*M*_*Q*^ab^_) → NE_1_(*M*_*Q*_). In the bases dual to $\det(W_{1}),\ldots ,\det(W_{\rho })$ of Pic*M*_*Q*_ and *W*_*ij*_, 1 ≤ *i* ≤ *ρ*, 1 ≤ *j* ≤ *r*_*i*_ of Pic*M*_*Q*^ab^_, this is

$$p:(d_{1,1},\ldots ,d_{1,r_{1}},d_{2,1},\ldots ,d_{\rho ,r_{\rho }})\mapsto \left(\sum_{i=1}^{r_{1}}d_{1i},\ldots ,\sum_{i=1}^{r_{\rho }}d_{\rho i}\right).$$

For *β* = (*d*_1_, …, *d*_*ρ*_), define

$$\epsilon (\beta )=\sum_{i=1}^{\rho }d_{i}(r_{i}-1).$$

Then, following [17, equation 3.2.1], the induced map of Novikov rings *N*(*M*_*Q*^ab^_) → *N*(*M*_*Q*_) sends

$$q^{\tilde{\beta }}\mapsto {\left(-1\right)}^{\epsilon (\beta )}q^{\beta },$$

where $\beta =p(\tilde{\beta })$. We write $\tilde{\beta }\to \beta$ if and only if $\tilde{\beta }\in {\mathrm{NE}}_{1}(M_{Q^{\mathrm{ab}}})$ and $p(\tilde{\beta })=\beta$.

For a representation theoretic bundle *E*_*G*_ of rank *r* on *M*_*Q*_, let *D*_1_, …, *D*_*r*_ be the divisors on *M*_*Q*^ab^_ giving the split bundle *E*_*T*_. Given $\tilde{d}\in {\mathrm{NE}}_{1}(M_{Q^{\mathrm{ab}}})$ define

$$I_{E_{G}}(\tilde{d})=\frac{\prod_{i=1}^{r}\prod_{m\leq 0}(D_{i}+mz)}{\prod_{i=1}^{r}\prod_{m\leq \langle \tilde{d},D_{i}\rangle }(D_{i}+mz)}.$$

Note that all but finitely many factors cancel here. If *E* is K-theoretically a representation theoretic bundle, in the sense that there exists *A*_*G*_, *B*_*G*_ such that

$$0\to A_{G}\to B_{G}\to E\to 0,$$

is an exact sequence, we define

6.5 $$I_{E}(\tilde{d})=\frac{I_{B_{G}(\tilde{d})}}{I_{A_{G}(\tilde{d})}}.$$

**Example 6.2**

The Euler sequence from proposition 2.3 shows that for the tangent bundle *T*_*M*_*Q*__

$$I_{T_{M_{Q}}}(\tilde{d})=\frac{\prod_{a\in Q_{1}^{ab}}\prod_{m\leq 0}(D_{a}+mz)}{\prod_{a\in Q_{1}^{ab}}\prod_{m\leq \langle \tilde{d},D_{a}\rangle }(D_{a}+mz)}\frac{\prod_{i=1}^{\rho }\prod_{j\neq k}\prod_{m\leq \langle \tilde{d},D_{ij}-D_{ik}\rangle }(D_{ij}-D_{ik}+mz)}{\prod_{i=1}^{\rho }\prod_{j\neq k}\prod_{m\leq 0}(D_{ij}-D_{ik}+mz)}.$$

Here, *D*_*ij*_ is the divisor corresponding to the tautological bundle *W*_*ij*_ for vertex *ij*, and *D*_*a*_: = − *D*_*s*(*a*)_ + *D*_*t*(*a*)_ is the divisor on *M*_*Q*^*ab*^_ corresponding to the arrow *a*∈*Q*^*ab*^_1_.

**Example 6.3**

If *X* is a quiver flag zero locus in *M*_*Q*_ defined by the bundle *E*_*G*_, then the adjunction formula (see equation (2.2)) implies that

$$I_{T_{X}}(\tilde{d})=I_{T_{M_{Q}}}(\tilde{d})/I_{E_{G}}(\tilde{d}).$$

Define the I-function of *X*⊂*M*_*Q*_ to be^2^

$$I_{X,M_{Q}}(z)=\sum_{d\in {\mathrm{NE}}_{1}(M_{Q})}\sum_{\tilde{d}\to d}{\left(-1\right)}^{\epsilon (d)}q^{d}I_{T_{X}}(\tilde{d}).$$

that $I_{T_{X}}(\tilde{d})$ is homogeneous of degree $\left(i(K_{X}),\tilde{d}\right)$, so defining the grading of *q*^*d*^ to be ( − *K*_*X*_, *d*), *I*_*X*,*M*_*Q*__(*z*) is homogeneous of degree 0. If *X* is Fano, we can write $I_{T_{X}}(\tilde{d})$ as

6.6 $$z^{\left(\omega_{X},\tilde{d}\right)}(b_{0}+b_{1}z^{-1}+b_{2}z^{-2}+\cdots ),b_{i}\in H^{2i}(X).$$

Since *I*_*X*,*M*_*Q*__ is invariant under the action of the Weyl group on the *D*_*ij*_, by viewing these as Chern roots of the tautological bundles *W*_*i*_ we can express it as a function in the Chern classes of the *W*_*i*_. We can, therefore, regard the I-function as an element of $H^{∙}(M_{Q},C)⊗N(M_{Q})⊗C[[z^{-1}]]$. If *X* is Fano,

6.7 $$I_{X,M_{Q}}(z)=1+z^{-1}C+O(z^{-2}),$$

where *O*(*z*^−2^) denotes terms of the form *αz*^*k*^ with *k* ≤  − 2 and $C\in H^{0}(M_{Q},C)⊗N(M_{Q})$; furthermore (by (6.6)) *C* vanishes if the Fano index of *X* is greater than 1.

**Theorem 6.4**

*Let* *X* *be a Fano quiver flag zero locus given by* (*Q*, *E*_*G*_), *and let* *j* : *X* → *M*_*Q*_ *be the embedding of* *X* *into the ambient quiver flag variety. Then*

$$J_{X}(0,z)=e^{-c/z}j^{*}I_{X,M_{Q}}(z)$$

*where* *c* = j*C.

**Remark 6.5**

Via the divisor equation and the string equation [25, §1.2], theorem 6.4 determines *J*_*X*_(*τ*, *z*) for *τ*∈*H*^0^(*X*)⊕*H*^2^(*X*).

### (c) Proof of theorem 6.4

Givental has defined [26,27] a Lagrangian cone $L_{X}$ in the symplectic vector space $H_{X}:=H^{*}(X,C)⊗N(X)⊗C((z^{-1}))$ that encodes all genus-zero Gromov–Witten invariants of *X*. Note that *J*_*X*_(*τ*, *z*)∈*H*_*X*_ for all *τ*. The J-function has the property that ( − *z*)*J*_*X*_(*τ*, − *z*) is the unique element of $L_{X}$ of the form

$$-z+\tau +O(z^{-1}),$$

(see [26, §9]) and this, together with the expression (6.7) for the I-function and the String Equation

$$J_{X}(\tau +c,z)=e^{c/z}J_{X}(\tau ,z),$$

shows that theorem 6.4 follows immediately from theorem 6.6 below. Theorem 6.6 is stronger: it does not require the hypothesis that the quiver flag zero locus *X* be Fano.

**Theorem 6.6**

*Let* *X* *be a quiver flag zero locus given by* (*Q*, *E*_*G*_), *and let* *j* : *X* → *M*_*Q*_ *be the embedding of* *X* *into the ambient quiver flag variety. Then* $(-z)j^{*}I_{X,M_{Q}}(-z)\in L_{X}.$

**Proof.**

Let $Y=\prod_{i=1}^{\rho }\mathrm{Gr}(H^{0}(W_{i}),r_{i})$. Denote by $Y^{ab}=\prod_{i=1}^{\rho }P{\left(H^{0}(W_{i})\right)}^{\times r_{i}}$ the Abelianization of *Y* . In §3, we constructed a vector bundle *V* on *Y* such that *M*_*Q*_ is cut out of *Y* by a regular section of *V* :

$$V=\overset{\rho }{\underset{i=2}{⨁}}Q_{i}⊗\frac{H^{0}{\left(W_{i}\right)}^{*}}{F_{i}^{*}},$$

where $F_{i}=\underset{t(a)=i}{⨁}Q_{s(a)}$. *V* is globally generated and hence convex. It is not representation theoretic, but it is K-theoretically: the sequence

$$0\to F_{i}^{*}⊗Q_{i}\to H^{0}{\left(W_{i}\right)}^{*}⊗Q_{i}\to H^{0}{\left(W_{i}\right)}^{*}⊗\frac{Q_{i}}{F_{i}^{*}}\to 0$$

is exact. Let *i*: *M*_*Q*_ → *Y* denote the inclusion.

Both *Y* and *M*_*Q*_ are GIT quotients by the same group; we can therefore canonically identify a representation theoretic vector bundle *E*′_*G*_ on *Y* such that *E*′_*G*_|_*M*_*Q*__ is *E*_*G*_. Our quiver flag zero locus *X* is cut out of *Y* by a regular section of *V* ′ = *V* ⊕*E*′_*G*_. Note that

$$\frac{I_{T_{M_{Q}}}(\tilde{d})}{I_{V}}(\tilde{d})=\frac{I_{T_{Y}}(\tilde{d})}{I_{V^{\prime }}}(\tilde{d}).$$

The I-function *I*_*X*,*M*_*Q*__ defined by considering *X* as a quiver flag zero locus in *M*_*Q*_ with the bundle *E*_*G*_ then coincides with the pullback i*I_*X*,*Y*_ of the I-function defined by considering *X* as a quiver flag zero locus in *Y* with the bundle *V* ′. It therefore suffices to prove that

$${\left(-z)(i\circ j\right)}^{*}I_{X,Y}(-z)\in L_{X}.$$

We consider a $C^{*}$-equivariant counterpart of the I-function, defined as follows. *λ* is the equivariant parameter given by the action on the bundle which is trivial on the base, as in (6.4). For a representation theoretic bundle *W*_*G*_ on *Y* , let *D*_1_, …, *D*_*r*_ be the divisors on *Y*^*ab*^ giving the split bundle *W*_*T*_, and for $\tilde{d}\in {\mathrm{NE}}_{1}(Y^{\mathrm{ab}})$ set

$$I_{W_{G}}^{C^{*}}(\tilde{d})=\frac{\prod_{i=1}^{r}\prod_{m\leq 0}(\lambda +D_{i}+mz)}{\prod_{i=1}^{r}\prod_{m\leq \langle \tilde{d},D_{i}\rangle }(\lambda +D_{i}+mz)}.$$

We extend this definition to bundles on *Y* – such as *V* ′ – that are only K-theoretically representation theoretic in the same way as (6.5). Let ${\tilde{s}}_{i}:=\dim H^{0}(W_{i}).$ Recalling that

$$I_{T_{Y}}(\tilde{d})=\frac{\prod_{i=1}^{\rho }\prod_{j\neq k}\prod_{m\leq \langle \tilde{d},D_{ij}-D_{ik}\rangle }(D_{ij}-D_{ik}+mz)}{\prod_{i=1}^{\rho }\prod_{j\neq k}\prod_{m\leq 0}(D_{ij}-D_{ik}+mz)}\frac{\prod_{i=1}^{\rho }\prod_{j=1}^{r_{i}}\prod_{m\leq 0}{\left(D_{ij}+mz\right)}^{{\tilde{s}}_{i}}}{\prod_{i=1}^{\rho }\prod_{j=1}^{r_{i}}\prod_{m\leq \langle \tilde{d},D_{ij}\rangle }{\left(D_{ij}+mz\right)}^{{\tilde{s}}_{i}}},$$

we define

$$I_{X,Y}^{C^{*}}(z)=\sum_{d\in {\mathrm{NE}}_{1}(Y)}\sum_{\tilde{d}\to d}{\left(-1\right)}^{\epsilon (d)}q^{d}I_{T_{Y}}(\tilde{d})\;/I_{V^{\prime }}^{C^{*}}(\tilde{d}).$$

The I-function *I*_*X*,*Y*_ can be obtained by setting *λ* = 0 in $I_{X,Y}^{C^{*}}$. In view of [24, Theorem 1.1], it therefore suffices to prove that

$$(-z)I_{X,Y}^{C^{*}}(-z)\in L_{e,V^{\prime }},$$

where $L_{e,V^{\prime }}$ is the Givental cone for the Gromov–Witten theory of *Y* twisted by the total Chern class **e** and the bundle *V* ′.

If *V* ′ were a representation theoretic bundle, this would follow immediately from the work of Ciocan–Fontanine–Kim–Sabbah: see the proof of theorem 6.1.2 in [17]. In fact *V* ′ is only K-theoretically representation theoretic, but their argument can be adjusted almost without change to this situation. Suppose that *A*_*G*_ and *B*_*G*_ are representation theoretic vector bundles, and that

$$0\to A_{G}\to B_{G}\to V\to 0,$$

is exact. Then we can also consider an exact sequence

$$0\to A_{T}\to B_{T}\to F\to 0,$$

on the Abelianization, and define *V*_*T*_: = *F*. Using the notation of the proof of [17, theorem 6.1.2], the point is that

$$△(V)△(A_{G})=△(B_{G}),$$

Here, △(V) is the twisting operator that appears in the quantum Lefschetz theorem [26]. We can then follow the same argument for

$$\frac{△(B_{G})}{△(A_{G})},$$

After Abelianizing, we obtain $△(B_{T})/△(A_{T})=△(F)$, and conclude that

$$(-z)I_{X,Y}^{C^{*}}(-z)\in L_{e,V^{\prime }},$$

as claimed. This completes the proof. ▪

## Supplementary Material

Data 1

## Supplementary Material

Data 2

## Supplementary Material

Tables and appendices

## Supplementary Material

Creative Commons Legal Code

## Supplementary Material

Read me file

## Note

1 Another proof of this, using different methods, has recently been given by Rachel Webb [5].

2 Note that usually the I-function is written as a function in (*τ*, *z*), just like the J-function. This is what you obtain if you set *τ* = 0 (the only case we need).

## Data accessibility

The electronic supplementary material contains the details of the computations finding all four-dimensional Fano quiver flag zero loci of codimension at most 4. The results of our computations are also contained there, in machine readable form. See the files called README.txt for details. The code to perform this and similar analyses, using the computational algebra system Magma [28], is available at the repository [29]. A database of Fano quiver flag varieties, which was produced as part of the calculation, is available at the repository [30]. The source code and data, but not the text of this paper, are released under a Creative Commons CC0 license: see the files called COPYING.txt for details. If you make use of the source code or data in an academic or commercial context, you should acknowledge this by including a reference or citation to this paper.
