# Supplementary material for: Four-dimensional Fano quiver flag zero loci
Source: Proc Math Phys Eng Sci. 2019 May 15;475(2225):20180791. doi: 10.1098/rspa.2018.0791 (PMC6545056; doi:10.1098/rspa.2018.0791)
Supplement: Tables and appendices [file rspa20180791supp3.pdf]

Appendix A. Computations

T. Coates (Imperial College London), E. Kalashnikov (Imperial College London), A. Kasprzyk (University of Nottingham)

In this appendix, we describe the computer search for four dimensional Fano quiver flag zero loci with codimension at most four. Code to perform this and similar analyses, using the computational algebra system Magma [3], is available at the repository [1]. A database of Fano quiver flag varieties, which was produced as part of the calculation, is available at the repository [2].

A.1. **Classifying quiver flag varieties.** The first step is to find all Fano quiver flag varieties of dimension at most 8. A non-negative integer matrix $A = [a_{i,j}]_{0 \le i,j \le \rho}$ and a dimension vector $\mathbf{r} \in \mathbb{Z}^{\rho+1}_{>0}$ determine a vertex-labelled directed multi-graph: the $\rho+1$ vertices are labelled by the r_i, and the adjacency matrix for the graph is A. Such a graph, if it is acyclic with a unique source, and the label of the source is 1, also determines a quiver flag variety. Two $(A, \mathbf{r})$ pairs can determine the same graph and hence the same quiver flag varieties.

Definition A.1. *A pair $(A, \mathbf{r})$ determining a quiver flag variety is in* normal form *if $\mathbf{r}$ is increasing and, under all permutations of the $\rho+1$ indices that preserve $\mathbf{r}$, the columns of A are lex minimal.*

Two pairs in normal form determine the same quiver flag variety (and hence the same graph) if and only if they are equal.

Recall that quiver flag varieties are towers of Grassmannians, and that the ith step in the tower is given by the relative Grassmannian $\mathrm{Gr}(\mathcal{F}_i, r_i)$, where $\mathcal{F}_i$ is a vector bundle of rank s_i. Using this construction it is easy to see that if $s_i = r_i$ then this quiver flag variety is equivalent to the quiver flag variety $\tilde{Q}$ with vertex i removed, and one arrow $k \to j$ for every path of the form $k \to i \to j$. Therefore we can assume that $s_i > r_i$, and hence that every vertex contributes strictly positively to the dimension of the quiver flag variety. With this constraint, there are only finitely many quiver flag varieties with dimension at most 8, and each such has at most 9 vertices.

The algorithm to build all quiver flag varieties with dimension at most 8 is as follows. Start with the set S of all Grassmannians of dimension at most 8. Given an element of S of dimension less than 8, add one extra labelled vertex and extra arrows into this vertex, in all possible ways such that the dimension of the resulting quiver flag variety is at most 8. Put these in normal form and include them in S. Repeat until there are no remaining elements of S of dimension less than 8.

In this way we obtain all quiver flag varieties of dimension at most 8. We then compute the ample cone and anti-canonical bundle for each, and discard any which are not Fano. We find 223044 Fano quiver flag varieties of dimension at most 8; 223017 of dimension $4 \le d \le 8$. Of these 50617 (respectively 50612) are non-toric quiver flag varieties.

	ρ							
d	1	2	3	4	5	6	7	8
1	1							
2	2	3						
3	2	8	11					
4	3	17	44	48				
5	2	27	118	262	231			
6	4	41	264	903	1647	1202		
7	2	54	498	2484	7005	10618	6541	
8	4	74	872	5852	23268	54478	69574	36880

Table 1. The number of Fano quiver flag varieties by dimension d and Picard rank ρ

Remark A.2. *In our codebase we define new Magma intrinsics* `QuiverFlagVariety(A,r)`*, which creates a quiver flag variety from an adjacency matrix A and dimension vector $\mathbf{r}$, and*

`QuiverFlagVarietyFano(id)`,

which creates a Fano quiver flag variety, in normal form, from its ID [1, 2]. We assign IDs to Fano quiver flag varieties of dimension at most 8, in the range $\{1 \ldots 223044\}$*, by placing them in normal form and then ordering them first by dimension, then by Picard rank, then lexicographically by dimension vector, then lexicographically by the columns of the adjacency matrix. We also define Magma intrinsics* `NefCone(Q)`, `MoriCone(Q)`, `PicardLattice(Q)`, *and* `CanonicalClass(Q)` *that compute the nef cone, Mori cone, Picard lattice and canonical class of a quiver flag variety* Q*, and an intrinsic* `PeriodSequence(Q,l)` *that computes the first* $l+1$ *terms of the Taylor expansion of the regularised quantum period of* Q*. See §A.5 for more details.*

A.2. **The class of vector bundles that we consider.** We consider all bundles E on a given quiver flag variety that:

- are direct sums of bundles of the form

$$L \otimes S^{\alpha_1}(W_1) \otimes \cdots \otimes S^{\alpha_\rho}(W_\rho) \tag{1}$$

  where each S^{α_i} is a non-negative Schur power and L is a nef line bundle; and
- have rank c, where c is four less than the dimension of the ambient quiver flag variety.

Remark 3.2 shows that non-negative Schur powers $S^{\alpha}(W_i)$ are globally generated, and Proposition 5.15 shows that nef line bundles are globally generated. Since the tensor product of globally generated vector bundles is globally generated, the first condition ensures that E is globally generated. In particular, therefore, the zero locus X of a generic section of E is smooth. The second condition ensures that the zero locus X, if non-empty, is a fourfold. Global generation also implies that the bundle E is convex, which allows us to compute the quantum period of X as described in §6.1.

Consider a summand as in (1). We can represent the partition α_i as a length r_i decreasing sequence of non-negative integers, and write $L = \bigotimes_{j=1}^{\rho} (\det W_j)^{a_j}$ where a_j may be negative. Therefore each such summand is determined by a length ρ sequence of *generalised partitions*: the partition (with possibly negative entries) corresponding to index i is $\alpha_i + (a_i, \ldots, a_i)$.

Remark A.3. *In our codebase we define a new Magma intrinsic*

`QuiverFlagBundle(Q,[A1,...,Ak])`

which creates a bundle of the above form, on the quiver flag variety Q*, from a sequence of generalised partitions* $(A1, \ldots, Ak)$*. We also define an intrinsic* `FirstChernClass(E)` *that computes the first Chern class of such a bundle* E*; intrinsics* `Degree(E)` *and* `EulerNumber(E)` *that compute the degree and Euler number[1] of the zero locus* X *of a generic section of* E*; and intrinsics* `HilbertCoefficients(E,l)` *and* `PeriodSequence(E,l)` *that compute the first* $l+1$ *terms of, respectively, the Hilbert series of* X *and the Taylor expansion of the regularised quantum period of* X*. See §A.5.*

A.3. **Classifying quiver flag bundles.** In this step, we describe the algorithm for determining all bundles on a given quiver flag variety that determine a smooth four-dimensional Fano quiver flag zero locus. A vector bundle as above is determined by a tuple $(A, \mathbf{r}, P)$, where A is an adjacency matrix, $\mathbf{r}$ is a dimension vector, and $P = (P_1, \ldots, P_k)$ is a sequence where each P_i is a length-ρ sequence of generalised partitions such that the jth partition in each P_i is of length r_j. Note that we regard the summands (1) in our vector bundles as unordered; also, as discussed above, different pairs $(A, \mathbf{r})$ can determine the same quiver flag variety. We therefore say that a tuple $(A, \mathbf{r}, P)$ is in *normal form* if the pair $(A, \mathbf{r})$ is in normal form, P is in lex order, and under all permutations of the vertices preserving these conditions, the sequence P is lex minimal; we work throughout with tuples in normal form.

Given a Fano quiver flag variety $M(Q, \mathbf{r})$ of dimension $4 + c$, $c \leq 4$, with anti-canonical class $-K_Q$ and nef cone $\mathrm{Nef}(Q)$, we search for all bundles E such that

- E is a direct sum of bundles of the form (1);
- $\mathrm{rank}(E) = c$;
- $-K_Q - c_1(E) \in \mathrm{Amp}(Q)$.

The last condition ensures that the associated quiver flag zero locus X, if non-empty, is Fano. We proceed as follows. We first find all possible summands that can occur; that is, all irreducible vector bundles E of the form (1) such that $\mathrm{rank}(E) \leq c$ and $-K_Q - c_1(E) \in \mathrm{Amp}(Q)$. Let $\mathrm{Irr}(Q)$ be the

[1] This is the Euler characteristic of X as a topological space.

set of all such bundles. Write $\mathrm{Irr}(Q) = \mathrm{Irr}(Q)_1 \sqcup \mathrm{Irr}(Q)_2$, where $\mathrm{Irr}(Q)_1$ contains vector bundles of rank strictly larger than 1, and $\mathrm{Irr}(Q)_2$ contains only line bundles. We then search for two vector bundles E_1, E_2 such that E_i is a direct sum of bundles from $\mathrm{Irr}(Q)_i$ and that $E = E_1 \oplus E_2$ satisfies the conditions above.

For each $x \in \mathrm{Nef}(Q)$ such that $-K_Q - x$ is ample, we find all possible ways to write x as

$$x = \sum_{i=1}^{l} a_i \tag{2}$$

where the a_i are (possibly repeated) elements of a Hilbert basis for $\mathrm{Nef}(Q)$. There are only finitely many decompositions (2); finding them efficiently is a knapsack-type problem that has already been solved [6]. For each $\tilde{c} \leq c$ and each partition of the a_i into at most $c/2$ groups $S_1, \ldots, S_s$, we find all possible choices of $F_1, \ldots, F_s \in \mathrm{Irr}_1$ such that

$$c_1(F_i) = \sum_{j \in S_i} a_j \qquad\qquad \mathrm{rank}(F_1) + \cdots + \mathrm{rank}(F_s) = \tilde{c}.$$

Set $E_1 = F_1 \oplus \cdots \oplus F_s$. Then for each $y \in \mathrm{Nef}(Q)$ such that $-K_Q - x - y$ is ample, we again find all ways of writing

$$y = \sum_{j=1}^{m} b_j$$

as a sum of Hilbert basis elements. Each partition of the b_j into $c - \tilde{c}$ groups gives a choice of nef line bundles $L_1, \ldots, L_{c-\tilde{c}} \in \mathrm{Irr}_2(Q)$, and we set $E_2 = \oplus L_j$.

Remark A.4. *Treating the higher rank summands* Irr_2 *and line bundles* Irr_1 *separately here is not logically necessary, but it makes a huge practical difference to the speed of the search.*

A.4. **Classifying quiver flag zero loci.** For each of the Fano quiver flag varieties Q of dimension between 4 and 8, found in §A.1, we use the algorithm described in §A.3 to find all bundles on Q of the form described in §A.2. This produces 10788446 bundles. Each such bundle E determines a quiver flag zero locus X that is either empty or a smooth Fano fourfold. We discard any varieties that are empty or disconnected, and for the remainder compute the first fifteen terms of the Taylor expansion of the regularised quantum period of X, using Theorem 6.4. For many of the quiver flag zero loci that we find, this computation is extremely expensive (the main factor is the Picard rank of the abelianised quiver, as this determines the size of the cohomology ring of the abelianised quiver flag variety where the computations are done; Gröbner basis calculations that allow the computation of products in this ring become more expensive as the size of the ring grows). In practice, therefore, it is essential to use the equivalences described in §4 to replace such quiver flag zero loci by equivalent and more tractable models. The number of equivalence classes is far smaller than the number of quiver flag zero loci that we found, and so this replaces roughly 10 million calculations, many of which are hard, by around half a million calculations, almost all of which are easy. In this way we find 749 period sequences. We record these period sequences, together with the construction, Euler number, and degree for a representative quiver flag zero locus, in Appendix B below. 141 of the period sequences that we find are new. Thus we find at least 141 new four-dimensional Fano manifolds[2].

Remark A.5. *A computationally cheap sufficient condition for a quiver flag zero locus to be empty arises as follows. If W is the tautological quotient bundle on $Gr(n,r)$, where $2r-1 > n$, then a generic global section of $\wedge^2 W$ or $\mathrm{Sym}^2 W$ has an empty zero locus. Thus if i is a vertex in a quiver Q such that all arrows into i are from the source, and $2r_i - 1 > n_{0i} = s_i$, then there are no global sections of $\wedge^2 W_i$ or $\mathrm{Sym}^2 W_i$ with non-empty zero locus: to see this, apply Proposition 3.1 to Q.*

[2] To be precise: we find at least 141 four-dimensional Fano manifolds for which the regularised quantum period was not previously known. The regularised quantum period of a Fano manifold X is expected to completely determine X. See [5,6] for known quantum periods.

A.5. **Cohomological computations for quiver flag zero loci.** In this section we describe how we compute the degree, Euler characteristic, Hilbert series, and Taylor expansion of the regularised quantum period for quiver flag varieties and quiver flag zero loci. This relies on Martin's integration formula [7] and Theorem 6.1.

Let V be a smooth projective variety with an action of G on V, let T be a maximal torus in G, and consider the GIT quotients $V/\!\!/G$ and $V/\!\!/T$ determined by a character of G. Let $\pi : V^{ss}(G)/T \to V/\!\!/G$ be the projection and $i : V^{ss}(G)/T \to V^{ss}(T)/T = V/\!\!/T$ be the inclusion. Let W be the Weyl group, and $e = \prod_{\lambda \in Roots(G)} c_1(L_\lambda)$, where L_λ is the line bundle on $V/\!\!/T$ associated to the character λ.

Theorem A.6 (Martin's Integration Formula, [7])**.** *For any $a \in H^*(V/\!\!/G, \mathbb{C})$ and any $\tilde{a} \in H^*(V/\!\!/T, \mathbb{C})$ satisfying $\pi^*(a) = i^*(\tilde{a})$*

$$\int_{V/\!\!/G} a = \frac{1}{|W|} \int_{V/\!\!/T} \tilde{a} \cup e.$$

If $a \in H^*(V/\!\!/G, \mathbb{C})$ and $\tilde{a} \in H^*(V/\!\!/T, \mathbb{C})$ satisfy $\pi^*(a) = i^*(\tilde{a})$ then we say that $\tilde{a}$ is a lift of a.

In our case the Abelianization $V/\!\!/T$ is a smooth toric variety, and the cohomology rings of such varieties, being Stanley–Reisner rings, are easy to work with computationally [3,13]. For example, we can use this to compute the number of components $h^0(X, \mathcal{O}_X)$ of a Fano quiver flag zero locus X. By Kodaira vanishing, $h^0(X, \mathcal{O}_X) = \chi(X)$, and applying the Hirzebruch–Riemann–Roch theorem gives

$$\chi(\mathcal{O}_X) = \int_X ch(\mathcal{O}_X) \cup Td(T_X) = \int_X Td(T_X). \tag{3}$$

We need to find a lift of the Todd class of T_X. Writing T_X as a K-theoretic quotient of representation theoretic bundles via the Euler sequence, as in the proof of Theorem 6.1, gives the lift that we seek; we then use Martin's formula to reduce the integral (3) to an integral in the cohomology ring of the Abelianization. The same approach allows us to compute the first two terms $\chi(X, -K_X)$, $\chi(X, -2K_X)$ of the Hilbert series of X – which determine the entire Hilbert series, since X is a Fano fourfold – as well as the degree and Euler characteristic of X. To compute the first few Taylor coefficients of the quantum period of X, we combine this approach with the explicit formula in Theorem 6.1.

Appendix B. Regularized quantum periods for quiver flag zero loci

T. Coates, E. Kalashnikov, A. Kasprzyk

B.1. **The table of representatives.** As described in Appendix A, we divided the 4-dimensional quiver flag zero loci X that we found into 749 buckets, according to the first 15 terms of the Taylor expansion of the regularised quantum period of X. We refer to these Taylor coefficients as the period sequence. Table 1 below gives, for each of the 749 period sequence buckets, a representative quiver flag zero locus X as well as the degree and Euler number of X. (In some cases we do not know that all the quiver flag zero loci in a bucket are isomorphic, but we checked that they all have the same degree, Euler number, and Hilbert series.) The quiver flag zero locus X is represented by the adjacency matrix and dimension vector of its ambient quiver flag variety $Y = M(Q, \mathbf{r})$, together with the sequence of generalised partitions that determine a vector bundle $E \to Y$ such that X is the zero locus of a generic section of E. The generalised partitions are written as Young diagrams, with:

- $\varnothing$ representing the empty Young diagram;
- a filled Young diagram, such as ■■, representing the dual to the vector bundle represented by the unfilled Young diagram □□.

Filled Young diagrams that occur always represent line bundles.

The entries in Table 1 give representatives of each period sequence bucket that are chosen so as to make the computation of geometric data (the period sequence etc.) straightforward[3]. Even though the Table is constructed by considering all four-dimensional Fano manifolds that occur as quiver flag zero loci in codimension up to four, in all four cases there is no tractable representative

[3]They are chosen to minimize the quantity $\sum_{i=0}^{\rho} r_i^2$, which is a rough proxy for the complexity of the Chow ring of the Abelianization.

as a quiver flag zero locus of low codimension. In these cases the Table contains a representative as a quiver flag zero locus in higher codimension; the reader who prefers models in lower-dimensional ambient spaces should consult Table 2.

Period ID	Adjacency matrix	Dimension vector	Generalized partitions
73	$\begin{smallmatrix} 0 & 1 & 1 & 3 & 3 \\ 0 & 0 & 1 & 0 & 0 \\ 0 & 0 & 0 & 0 & 0 \\ 0 & 0 & 0 & 0 & 0 \\ 0 & 1 & 0 & 0 & 0 \end{smallmatrix}$	1 2 2 2 2	$(\varnothing, \Box, \Box, \varnothing)$
144	$\begin{smallmatrix} 0 & 3 & 1 & 2 & 3 \\ 0 & 0 & 0 & 1 & 0 \\ 0 & 0 & 0 & 0 & 0 \\ 0 & 0 & 0 & 0 & 0 \\ 0 & 0 & 1 & 0 & 0 \end{smallmatrix}$	1 1 2 2 2	$(\varnothing, \Box, \Box, \varnothing)$
439	$\begin{smallmatrix} 0 & 1 & 5 \\ 0 & 0 & 0 \\ 0 & 1 & 0 \end{smallmatrix}$	1 4 4	$\left(\begin{smallmatrix}\Box\\\Box\\\Box\\\Box\end{smallmatrix}, \varnothing\right), \left(\begin{smallmatrix}\Box\\\Box\\\Box\\\Box\end{smallmatrix}, \varnothing\right), \left(\begin{smallmatrix}\Box\\\Box\\\Box\\\Box\end{smallmatrix}, \varnothing\right), \left(\begin{smallmatrix}\Box\\\Box\\\Box\\\Box\end{smallmatrix}, \varnothing\right)$
552	$\begin{smallmatrix} 0 & 0 & 5 \\ 0 & 0 & 0 \\ 0 & 1 & 0 \end{smallmatrix}$	1 2 4	$\left(\Box, \begin{smallmatrix}\Box\\\Box\\\Box\\\Box\end{smallmatrix}\right), \left(\begin{smallmatrix}\Box\\\Box\end{smallmatrix}, \varnothing\right), \left(\begin{smallmatrix}\Box\\\Box\end{smallmatrix}, \varnothing\right)$

TABLE 2. Representatives for certain Period IDs in codimension at most four

Remark B.1. *The data in Tables 1 and 2 can also be found, in machine readable form, in the ancillary files that accompany this paper.*

B.2. **The table of period sequences.** Table 2 records the first 8 terms of the period sequence, $\alpha_0, \alpha_1, \ldots, \alpha_7$, for each of the 749 period sequence buckets. It also records, where they exist, the names of known four-dimensional Fano manifolds which have the same first fifteen terms of the period sequence. Notation is as follows:

- $\mathbb{P}^n$ denotes n-dimensional complex projective space;
- Q^n denotes a quadric hypersurface in $\mathbb{P}^{n+1}$;
- FI^4_k is the kth four-dimensional Fano manifold of index 3, as in [5, §5];
- V^4_k is the kth four-dimensional Fano manifold of index 2 and Picard rank 1, as in [5, §6.1];
- MW^4_k is the kth four-dimensional Fano manifold of index 2 and Picard rank at least 2, as in [5, §6.2];
- $\mathrm{BØS}^4_k$ is the kth four-dimensional toric Fano manifold, as in [5, §7];
- Str_k are the Strangeway fourfolds described in [5, §8];
- CKP_k is the kth four-dimensional toric complete intersection, as in [6];
- S^2_k denotes the del Pezzo surface of degree k;
- V^3_k denotes the three-dimensional Fano manifold of Picard rank 1, Fano index 1, and degree k;
- B^3_k denotes the three-dimensional Fano manifold of Picard rank 1, Fano index 2, and degree $8k$;
- $\mathrm{MM}^3_{\rho-k}$ denotes the kth entry in the Mori–Mukai list of three-dimensional Fano manifolds of Picard rank ρ [8–12]. We use the ordering as in [4], which agrees with the original papers of Mori–Mukai except when $\rho = 4$.

Remark B.2. *It appears from Table 2 as if the period sequences with IDs* 72 *and* 73 *might coincide. This is not the case. The coefficients* α_8*,* α_9*, and* α_{10} *in these cases are:*

Period ID	α_8	α_9	α_{10}
72	32830	212520	1190952
73	32830	227640	1190952

Remark B.3. *590 of the period sequences that we find coincide with period sequences for toric complete intersections, at least for the first 15 terms. 579 of these are realised by quiver flag zero loci that are also toric complete intersections. For the remaining 11 cases – period sequences with IDs 17, 48, 73, 144, 145, 158, 191, 204, 256, 280, and 282 – there is no model as a toric complete intersection that is also a quiver flag zero locus in codimension at most four. In four of these cases – with IDs 17, 48, 144, and 256 – the toric complete intersection period sequence is realised by a smooth four-dimensional toric variety.*

Remark B.4. *An earlier version of this paper omitted two of the period sequences that we find below, due to erroneous hand calculations in special cases. In this version all computations are performed in software, in a uniform way; we believe that this makes them more likely to be correct.*

Funding statement. EK was supported by the Natural Sciences and Engineering Research Council of Canada, and by the EPSRC Centre for Doctoral Training in Geometry and Number Theory at the Interface, grant number EP/L015234/1. TC was supported by ERC Consolidator Grant number 682602 and EPSRC Programme Grant EP/N03189X/1. AK was supported by EPSRC Fellowship grant EP/N022513/1.

Acknowledgements. The computations that underpin this work were performed on the Imperial College HPC cluster. We thank Andy Thomas, Matt Harvey, and the Research Computing Service team at Imperial for invaluable technical assistance.

Ethics statement. This research did not involve human or animal subjects.

Competing interests statement. We have no competing interests.

Authors' contributions. EK is author of the main body of the paper. TC, EK and AK are joint authors of the appendices.

Data accessibility. The Electronic Supplementary Material contains the results of our computations, in machine readable form. See the files called README.txt for details. The code to perform this and similar analyses, using the computational algebra system Magma [3], is available at the repository [1]. A database of Fano quiver flag varieties, which was produced as part of the calculation, is available at the repository [2]. The source code and data, but not the text of this paper, are released under a Creative Commons CC0 license: see the files called COPYING.txt for details. If you make use of the source code or data in an academic or commercial context, you should acknowledge this by including a reference or citation to this paper.

Table 3: Certain 4-dimensional Fano manifolds with Fano index 1 that arise as quiver flag zero loci

Period ID	Adjacency matrix	Dimension vector	Generalized partitions	Degree	Euler Number
1	0 5<br>0 0	1 1		625	5
2	0 0 5<br>0 0 0<br>0 3 0	1 1 1	(□, ■),(□, ∅)	512	8
3	0 6<br>0 0	1 1	(□□)	512	6
4	0 1 5<br>0 0 0<br>0 1 0	1 1 1	(□, □)	431	9
5	0 2 3<br>0 0 0<br>0 1 0	1 1 1		513	9
6	0 1 3<br>0 0 0<br>0 2 0	1 1 1		513	9
7	0 1 2 2<br>0 0 1 0<br>0 0 0 0<br>0 1 0 0	1 1 1 1		459	12
8	0 0 2 3<br>0 0 0 0<br>0 1 0 0<br>0 1 1 0	1 1 1 1	(□, ∅, ∅)	417	13
9	0 2 4<br>0 0 0<br>0 1 0	1 1 1	(∅, □□)	486	12
10	0 3 3<br>0 0 1<br>0 0 0	1 1 1	(□, □)	432	9
11	0 1 2 2<br>0 0 0 0<br>0 1 0 0<br>0 1 0 0	1 1 1 1		405	12
12	0 1 3 2<br>0 0 0 0<br>0 0 0 1<br>0 1 0 0	1 1 1 1	(□, □, ∅)	384	13
13	0 0 5<br>0 0 0<br>0 3 0	1 1 1	(□, ∅),(□, ∅)	351	9

Continued on next page.

Continued from previous page.

Period ID	Adjacency matrix	Dimension vector	Generalized partitions	Degree	Euler Number
14	0 0 3<br>0 0 0<br>0 5 0	1 1 1	$(\square,\blacksquare),(\square,\blacksquare)$	486	9
15	0 5 2<br>0 0 1<br>0 0 0	1 1 2	$(\square,\square)$	433	9
16	0 0 3 3<br>0 0 0 0<br>0 1 0 0<br>0 1 0 0	1 1 1 1	$(\square,\varnothing,\varnothing)$	401	13
17	0 3 5 2<br>0 0 0 1<br>0 0 0 0<br>0 0 0 0	1 1 1 2	$(\varnothing,\square,\varnothing),(\varnothing,\square,\varnothing),(\varnothing,\square,\square)$	406	13
18	0 0 0 3 3<br>0 0 0 0 0<br>0 0 0 0 0<br>0 1 1 0 0<br>0 1 1 0 0	1 1 1 1 1	$(\varnothing,\square,\varnothing,\varnothing),(\square,\varnothing,\varnothing,\varnothing)$	322	18
19	0 0 6<br>0 0 0<br>0 2 0	1 1 1	$(\varnothing,\square\square),(\square,\varnothing)$	378	10
20	0 0 0 4<br>0 0 0 0<br>0 0 0 0<br>0 1 1 0	1 1 1 2	$(\varnothing,\square,\varnothing),(\square,\varnothing,\varnothing)$	358	13
21	0 0 5 2<br>0 0 0 0<br>0 0 0 1<br>0 1 0 0	1 1 1 2	$(\varnothing,\square,\square),(\square,\varnothing,\varnothing)$	347	13
22	0 0 1 5<br>0 0 0 0<br>0 0 0 0<br>0 2 1 0	1 1 1 1	$(\varnothing,\square,\square),(\square,\varnothing,\varnothing)$	330	14
23	0 0 5<br>0 0 0<br>0 3 0	1 1 1	$(\square,\blacksquare),(\square,\square)$	297	13
24	0 5<br>0 0	1 2	$\left(\begin{smallmatrix}\square\\\square\end{smallmatrix}\right),\left(\begin{smallmatrix}\square\\\square\end{smallmatrix}\right)$	405	6
25	0 1 4<br>0 0 0<br>0 1 0	1 2 2	$\left(\begin{smallmatrix}\square\\\square\end{smallmatrix},\varnothing\right),\left(\begin{smallmatrix}\square\\\square\end{smallmatrix},\varnothing\right)$	325	10

Continued on next page.

Continued from previous page.

Period ID	Adjacency matrix	Dimension vector	Generalized partitions	Degree	Euler Number
26	$\begin{matrix} 0 & 0 & 0 & 0 & 4 \\ 0 & 0 & 0 & 0 & 0 \\ 0 & 0 & 0 & 0 & 0 \\ 0 & 0 & 0 & 0 & 0 \\ 0 & 1 & 1 & 1 & 0 \end{matrix}$	1 1 1 1 2	$(\varnothing,\varnothing,\square,\varnothing),(\varnothing,\square,\varnothing,\varnothing),(\square,\varnothing,\varnothing,\varnothing)$	290	18
27	$\begin{matrix} 0 & 7 \\ 0 & 0 \end{matrix}$	1 1	$(\square\square),(\square\square)$	324	12
28	$\begin{matrix} 0 & 1 & 6 \\ 0 & 0 & 0 \\ 0 & 1 & 0 \end{matrix}$	1 1 1	$(\varnothing,\square\square),(\square,\square)$	292	14
29	$\begin{matrix} 0 & 5 & 4 \\ 0 & 0 & 0 \\ 0 & 0 & 0 \end{matrix}$	1 1 2	$(\square,\square),(\square,\square)$	273	9
30	$\begin{matrix} 0 & 1 & 1 & 5 \\ 0 & 0 & 0 & 0 \\ 0 & 0 & 0 & 0 \\ 0 & 1 & 1 & 0 \end{matrix}$	1 1 1 1	$(\varnothing,\square,\square),(\square,\varnothing,\square)$	261	17
31	$\begin{matrix} 0 & 2 & 5 \\ 0 & 0 & 0 \\ 0 & 1 & 0 \end{matrix}$	1 1 1	$(\square,\square),(\square,\square)$	244	16
32	$\begin{matrix} 0 & 5 & 5 \\ 0 & 0 & 0 \\ 0 & 0 & 0 \end{matrix}$	1 1 4	$\left(\square,\begin{array}{c}\square\\\square\\\square\end{array}\right)$	225	5
33	$\begin{matrix} 0 & 6 \\ 0 & 0 \end{matrix}$	1 1	$(\square\square\square)$	243	27
34	$\begin{matrix} 0 & 1 & 5 \\ 0 & 0 & 0 \\ 0 & 1 & 0 \end{matrix}$	1 1 1	$(\square,\square\square)$	211	29
35	$\begin{matrix} 0 & 1 & 4 \\ 0 & 0 & 0 \\ 0 & 1 & 0 \end{matrix}$	1 1 1		544	8
36	$\begin{matrix} 0 & 0 & 4 \\ 0 & 0 & 0 \\ 0 & 4 & 0 \end{matrix}$	1 1 1	$(\square,\blacksquare),(\square,\blacksquare)$	512	8
37	$\begin{matrix} 0 & 1 & 2 & 2 \\ 0 & 0 & 0 & 0 \\ 0 & 0 & 0 & 1 \\ 0 & 1 & 0 & 0 \end{matrix}$	1 1 1 1		464	12
38	$\begin{matrix} 0 & 0 & 1 & 4 \\ 0 & 0 & 0 & 0 \\ 0 & 0 & 0 & 0 \\ 0 & 3 & 1 & 0 \end{matrix}$	1 1 1 1	$(\square,\varnothing,\blacksquare),(\square,\square,\blacksquare)$	431	11

Continued on next page.

Continued from previous page.

Period ID	Adjacency matrix	Dimension vector	Generalized partitions	Degree	Euler Number
39	$\begin{matrix}0&1&5\\0&0&0\\0&1&0\end{matrix}$	1 1 1	$(\varnothing, \square\square)$	480	8
40	$\begin{matrix}0&2&4\\0&0&0\\0&1&0\end{matrix}$	1 1 1	$(\square, \square)$	416	10
41	$\begin{matrix}0&1&1&4\\0&0&0&0\\0&0&0&0\\0&1&1&0\end{matrix}$	1 1 1 1	$(\square, \varnothing, \square)$	400	12
42	$\begin{matrix}0&1&1&4\\0&0&1&0\\0&0&0&0\\0&1&0&0\end{matrix}$	1 1 1 1	$(\varnothing, \square, \square)$	383	13
43	$\begin{matrix}0&2&4\\0&0&0\\0&1&0\end{matrix}$	1 1 1	$(\square\square, \varnothing)$	350	12
44	$\begin{matrix}0&1&1&3\\0&0&1&0\\0&0&0&0\\0&1&0&0\end{matrix}$	1 1 1 1		480	12
45	$\begin{matrix}0&0&4\\0&0&0\\0&4&0\end{matrix}$	1 1 1	$(\square, \blacksquare), (\square, \varnothing)$	432	9
46	$\begin{matrix}0&0&1&3\\0&0&1&0\\0&0&0&0\\0&4&0&0\end{matrix}$	1 1 1 1	$(\square, \varnothing, \blacksquare), (\square, \varnothing, \blacksquare)$	496	12
47	$\begin{matrix}0&0&1&3\\0&0&0&0\\0&1&0&0\\0&1&1&0\end{matrix}$	1 1 1 1		432	12
48	$\begin{matrix}0&3&5&0\\0&0&0&1\\0&0&0&2\\0&0&0&0\end{matrix}$	1 1 1 2	$(\varnothing, \varnothing, \square), (\varnothing, \varnothing, \square)$	433	13
49	$\begin{matrix}0&2&2&2\\0&0&0&0\\0&0&0&1\\0&0&0&0\end{matrix}$	1 1 1 1		432	12

Continued on next page.

Continued from previous page.

Period ID	Adjacency matrix	Dimension vector	Generalized partitions	Degree	Euler Number
50	0 1 1 1 2<br>0 0 0 1 0<br>0 0 0 0 0<br>0 0 1 0 0<br>0 1 0 0 0	1 1 1 1 1		432	16
51	0 0 2 3<br>0 0 0 0<br>0 1 0 0<br>0 1 0 0	1 1 1 1		400	12
52	0 0 1 1 3<br>0 0 0 0 0<br>0 0 0 1 0<br>0 1 0 0 0<br>0 1 1 0 0	1 1 1 1 1	(□, ∅, ∅, ∅)	384	16
53	0 0 0 4<br>0 0 0 0<br>0 0 0 0<br>0 2 3 0	1 1 1 1	(∅, □, ∅), (□, □, ■■)	378	12
54	0 1 1 4<br>0 0 1 0<br>0 0 0 0<br>0 1 0 0	1 1 1 1	(∅, ∅, □□)	464	16
55	0 1 3 2<br>0 0 0 0<br>0 0 0 1<br>0 1 0 0	1 1 1 1	(∅, □, □)	416	12
56	0 1 2 3<br>0 0 1 0<br>0 0 0 0<br>0 1 0 0	1 1 1 1	(∅, □, □)	384	13
57	0 0 0 4 1<br>0 0 0 0 0<br>0 0 0 0 0<br>0 0 0 0 1<br>0 2 2 0 0	1 1 1 1 1	(∅, □, □, ■), (□, ∅, □, ■)	384	16
58	0 1 1 1 3<br>0 0 0 1 0<br>0 0 0 0 0<br>0 0 1 0 0<br>0 1 0 0 0	1 1 1 1 1	(∅, □, ∅, □)	357	17
59	0 1 2 3<br>0 0 0 0<br>0 0 0 0<br>0 1 1 0	1 1 1 1	(□, □, ∅)	336	14

Continued on next page.

Continued from previous page.

Period ID	Adjacency matrix	Dimension vector	Generalized partitions	Degree	Euler Number
60	0 0 0 4 1<br>0 0 0 0 0<br>0 0 0 0 0<br>0 0 0 0 1<br>0 2 2 0 0	1 1 1 1 1	(∅,□,∅,∅),(□,∅,□,■)	357	16
61	0 0 3 4<br>0 0 0 0<br>0 1 0 0<br>0 1 0 0	1 1 1 1	(∅,□,□),(□,∅,∅)	336	13
62	0 0 4<br>0 0 0<br>0 4 0	1 1 1	(∅,□□),(□,∅)	324	12
63	0 0 1 4<br>0 0 0 0<br>0 0 0 0<br>0 3 1 0	1 1 1 1	(□,∅,∅),(□,∅,∅)	336	12
64	0 0 1 4<br>0 0 0 0<br>0 0 0 0<br>0 3 1 0	1 1 1 1	(□,∅,∅),(□,□,■)	303	13
65	0 0 4<br>0 0 0<br>0 4 0	1 1 1	(□,■),(□□,■)	270	9
66	0 0 1 3<br>0 0 1 0<br>0 0 0 0<br>0 4 0 0	1 1 1 1	(□,∅,■),(□,∅,∅)	480	12
67	0 1 3 2<br>0 0 0 0<br>0 0 0 0<br>0 1 0 0	1 1 1 1		432	12
68	0 0 5<br>0 0 0<br>0 3 0	1 1 1	(∅,□□),(□,■)	432	8
69	0 0 0 3<br>0 0 1 0<br>0 0 0 0<br>0 3 2 0	1 1 1 1	(∅,□,■),(∅,□,∅)	368	13
70	0 1 4<br>0 0 0<br>0 2 0	1 1 1	(□,□)	352	12

Continued on next page.

Continued from previous page.

Period ID	Adjacency matrix	Dimension vector	Generalized partitions	Degree	Euler Number
71	0 0 0 1 2<br>0 0 1 0 0<br>0 0 0 1 0<br>0 0 0 0 0<br>0 2 1 0 0	1 1 1 1 1		448	16
72	0 0 0 1 3<br>0 0 0 1 0<br>0 0 0 0 0<br>0 0 1 0 0<br>0 3 1 0 0	1 1 1 1 1	(∅, □, ∅, ∅),(□, ∅, ∅, ■)	389	16
73	0 3 3 0 4<br>0 0 0 0 0<br>0 0 0 1 0<br>0 0 0 0 0<br>0 0 0 1 0	1 1 1 2 2	(∅, ∅, □, ∅),(∅, ∅, □, ∅),(□, ∅, ∅, □)	369	17
74	0 0 0 2<br>0 0 2 0<br>0 0 0 0<br>0 3 2 0	1 1 1 1	(∅, □, ■),(∅, □, ∅)	352	13
75	0 0 1 4<br>0 0 0 0<br>0 0 0 0<br>0 3 1 0	1 1 1 1	(∅, □, □),(□, ∅, ■)	368	12
76	0 1 2 4<br>0 0 1 0<br>0 0 0 0<br>0 1 0 0	1 1 2 2	(∅, □, □)	337	13
77	0 0 0 3 3<br>0 0 0 0 0<br>0 0 0 0 0<br>0 0 1 0 0<br>0 2 1 0 0	1 1 1 1 1	(∅, □, ∅, ∅),(□, ∅, □, ■)	347	16
78	0 0 0 3 2<br>0 0 4 0 0<br>0 0 0 0 0<br>0 0 0 0 1<br>0 1 0 0 0	1 1 1 1 2	(■, □, ∅, ∅),(■, □, ∅, □),(□, ∅, ∅, ∅)	331	17
79	0 0 1 4<br>0 0 1 0<br>0 0 0 0<br>0 3 0 0	1 1 1 1	(∅, □, □),(□, ∅, ■)	335	15

Continued on next page.

Continued from previous page.

Period ID	Adjacency matrix	Dimension vector	Generalized partitions	Degree	Euler Number
80	0 0 3 3<br>0 0 0 0<br>0 1 0 0<br>0 2 0 0	1 1 1 1	$(\square,\varnothing,\varnothing),(\square,\varnothing,\varnothing)$	305	13
81	0 0 1 5<br>0 0 0 0<br>0 0 0 0<br>0 2 1 0	1 1 1 1	$(\varnothing,\varnothing,\square\square),(\square,\varnothing,\varnothing)$	368	12
82	0 0 3 3<br>0 0 0 0<br>0 1 0 0<br>0 1 0 0	1 1 1 1	$(\varnothing,\square,\square)$	352	12
83	0 0 2 4<br>0 0 0 0<br>0 0 0 0<br>0 2 1 0	1 1 1 1	$(\varnothing,\square,\square),(\square,\varnothing,\varnothing)$	336	14
84	0 0 0 3 2<br>0 0 0 0 0<br>0 0 0 0 0<br>0 0 0 0 1<br>0 2 2 0 0	1 1 1 1 1	$(\varnothing,\square,\square,\blacksquare),(\square,\varnothing,\varnothing,\varnothing)$	352	16
85	0 0 1 5<br>0 0 0 0<br>0 0 0 0<br>0 2 1 0	1 1 1 1	$(\varnothing,\varnothing,\square\square),(\square,\square,\blacksquare)$	346	12
86	0 0 0 2 3<br>0 0 0 0 0<br>0 0 0 0 0<br>0 0 1 0 0<br>0 2 1 1 0	1 1 1 1 1	$(\varnothing,\square,\varnothing,\varnothing),(\square,\varnothing,\square,\blacksquare)$	310	17
87	0 0 2 4<br>0 0 0 0<br>0 1 0 0<br>0 1 1 0	1 1 1 1	$(\varnothing,\square,\square),(\square,\varnothing,\varnothing)$	299	15
88	0 0 1 3 3<br>0 0 0 0 0<br>0 0 0 0 0<br>0 1 0 0 0<br>0 1 1 0 0	1 1 1 1 1	$(\varnothing,\square,\square,\varnothing),(\square,\varnothing,\varnothing,\varnothing)$	289	18
89	0 0 0 3<br>0 0 1 0<br>0 0 0 0<br>0 3 2 0	1 1 1 1	$(\varnothing,\square,\varnothing),(\square,\varnothing,\varnothing)$	304	13

Continued on next page.

Continued from previous page.

Period ID	Adjacency matrix	Dimension vector	Generalized partitions	Degree	Euler Number
90	0 0 1 1 4<br>0 0 0 0 0<br>0 0 0 1 0<br>0 0 0 0 0<br>0 2 1 0 0	1 1 1 1 1	(∅, ∅, □, □),(□, ∅, ∅, ∅)	309	18
91	0 0 3 2<br>0 0 0 0<br>0 1 0 1<br>0 2 0 0	1 1 1 1	(□, ∅, ∅),(□, ∅, ∅)	273	15
92	0 0 1 1 4<br>0 0 0 0 0<br>0 0 0 0 0<br>0 0 0 0 0<br>0 2 1 1 0	1 1 1 1 1	(∅, ∅, □, □),(□, □, ∅, ■)	299	17
93	0 0 2 4<br>0 0 0 0<br>0 0 0 0<br>0 2 1 0	1 1 1 1	(∅, □, □),(□, □, ■)	282	14
94	0 0 1 4<br>0 0 0 0<br>0 0 0 0<br>0 3 1 0	1 1 1 1	(□, ∅, ■),(□, ∅, □)	288	8
95	0 0 2 4<br>0 0 0 0<br>0 0 0 0<br>0 2 1 0	1 1 1 1	(∅, □□, ∅),(□, ∅, ∅)	282	18
96	0 0 1 4<br>0 0 0 0<br>0 1 0 0<br>0 1 1 0	1 1 1 1	(□, ∅, □)	266	16
97	0 0 1 4<br>0 0 0 0<br>0 0 0 0<br>0 3 1 0	1 1 1 1	(□, ∅, ■),(□, □, ∅)	249	17
98	0 1 4<br>0 0 0<br>0 2 0	1 1 1	(□□, ∅)	216	16
99	0 0 1 4<br>0 0 1 0<br>0 0 0 0<br>0 3 0 0	1 1 1 1	(∅, ∅, □□),(□, ∅, ■)	480	16

Continued on next page.

Continued from previous page.

Period ID	Adjacency matrix	Dimension vector	Generalized partitions	Degree	Euler Number
100	$\begin{matrix} 0 & 0 & 2 & 3 \\ 0 & 0 & 1 & 0 \\ 0 & 0 & 0 & 0 \\ 0 & 3 & 0 & 0 \end{matrix}$	1 1 1 1	$(\varnothing, \square, \square), (\square, \varnothing, \blacksquare)$	384	13
101	$\begin{matrix} 0 & 1 & 4 \\ 0 & 0 & 0 \\ 0 & 1 & 0 \end{matrix}$	1 2 2	$\left(\varnothing, \begin{smallmatrix}\square\\\square\end{smallmatrix}\right), \left(\begin{smallmatrix}\square\\\square\end{smallmatrix}, \varnothing\right)$	352	9
102	$\begin{matrix} 0 & 0 & 5 \\ 0 & 0 & 0 \\ 0 & 1 & 0 \end{matrix}$	1 1 2	$\left(\varnothing, \begin{smallmatrix}\square\\\square\end{smallmatrix}\right), \left(\varnothing, \begin{smallmatrix}\square\\\square\end{smallmatrix}\right), (\square, \varnothing)$	320	10
103	$\begin{matrix} 0 & 0 & 1 & 1 & 3 \\ 0 & 0 & 0 & 1 & 0 \\ 0 & 0 & 0 & 0 & 0 \\ 0 & 0 & 1 & 0 & 0 \\ 0 & 3 & 0 & 0 & 0 \end{matrix}$	1 1 1 1 1	$(\varnothing, \square, \varnothing, \square), (\square, \varnothing, \varnothing, \blacksquare)$	362	17
104	$\begin{matrix} 0 & 0 & 0 & 3 & 2 \\ 0 & 0 & 0 & 0 & 0 \\ 0 & 0 & 0 & 0 & 0 \\ 0 & 3 & 3 & 0 & 1 \\ 0 & 0 & 0 & 0 & 0 \end{matrix}$	1 1 1 1 2	$(\varnothing, \square, \blacksquare, \square), (\square, \varnothing, \blacksquare, \square)$	305	18
105	$\begin{matrix} 0 & 0 & 2 & 4 \\ 0 & 0 & 0 & 0 \\ 0 & 0 & 0 & 0 \\ 0 & 2 & 1 & 0 \end{matrix}$	1 1 1 1	$(\varnothing, \varnothing, \square\square), (\square, \square, \blacksquare)$	352	16
106	$\begin{matrix} 0 & 0 & 3 & 3 \\ 0 & 0 & 0 & 0 \\ 0 & 0 & 0 & 1 \\ 0 & 2 & 0 & 0 \end{matrix}$	1 1 1 1	$(\varnothing, \square, \square), (\square, \varnothing, \varnothing)$	304	14
107	$\begin{matrix} 0 & 0 & 0 & 3 & 2 \\ 0 & 0 & 0 & 0 & 0 \\ 0 & 0 & 0 & 0 & 0 \\ 0 & 0 & 0 & 0 & 0 \\ 0 & 2 & 2 & 1 & 0 \end{matrix}$	1 1 1 1 1	$(\varnothing, \square, \square, \blacksquare), (\square, \varnothing, \square, \blacksquare)$	304	17
108	$\begin{matrix} 0 & 0 & 1 & 3 & 2 \\ 0 & 0 & 0 & 0 & 0 \\ 0 & 0 & 0 & 0 & 0 \\ 0 & 0 & 0 & 0 & 1 \\ 0 & 2 & 1 & 0 & 0 \end{matrix}$	1 1 1 1 1	$(\varnothing, \square, \square, \varnothing), (\square, \varnothing, \varnothing, \varnothing)$	283	18
109	$\begin{matrix} 0 & 0 & 5 \\ 0 & 0 & 0 \\ 0 & 1 & 0 \end{matrix}$	1 1 2	$\left(\varnothing, \begin{smallmatrix}\square\\\square\end{smallmatrix}\right), \left(\varnothing, \begin{smallmatrix}\square\\\square\end{smallmatrix}\right), \left(\varnothing, \begin{smallmatrix}\square\\\square\end{smallmatrix}\right)$	272	8

Continued on next page.

Continued from previous page.

Period ID	Adjacency matrix	Dimension vector	Generalized partitions	Degree	Euler Number
110	$\begin{matrix} 0 & 0 & 0 & 2 \\ 0 & 0 & 1 & 0 \\ 0 & 0 & 0 & 0 \\ 0 & 3 & 3 & 0 \end{matrix}$	1 1 1 1	$(\varnothing, \square, \blacksquare), (\square, \square, \blacksquare)$	256	17
111	$\begin{matrix} 0 & 1 & 6 \\ 0 & 0 & 0 \\ 0 & 1 & 0 \end{matrix}$	1 1 1	$(\varnothing, \square\square), (\varnothing, \square\square)$	320	0
112	$\begin{matrix} 0 & 2 & 5 \\ 0 & 0 & 0 \\ 0 & 1 & 0 \end{matrix}$	1 1 1	$(\varnothing, \square\square), (\square, \square)$	304	12
113	$\begin{matrix} 0 & 3 & 4 \\ 0 & 0 & 0 \\ 0 & 1 & 0 \end{matrix}$	1 1 1	$(\square, \square), (\square, \square)$	272	15
114	$\begin{matrix} 0 & 1 & 1 & 5 \\ 0 & 0 & 1 & 0 \\ 0 & 0 & 0 & 0 \\ 0 & 1 & 0 & 0 \end{matrix}$	1 1 1 1	$(\varnothing, \varnothing, \square\square), (\varnothing, \square, \square)$	282	16
115	$\begin{matrix} 0 & 1 & 4 \\ 0 & 0 & 0 \\ 0 & 1 & 0 \end{matrix}$	1 1 2	$(\square, \square)$	272	10
116	$\begin{matrix} 0 & 0 & 1 & 4 \\ 0 & 0 & 0 & 0 \\ 0 & 0 & 0 & 0 \\ 0 & 1 & 1 & 0 \end{matrix}$	1 1 2 2	$\left(\varnothing, \begin{smallmatrix}\square\\\square\end{smallmatrix}, \varnothing\right), \left(\varnothing, \begin{smallmatrix}\square\\\square\end{smallmatrix}, \varnothing\right), (\square, \varnothing, \varnothing)$	257	15
117	$\begin{matrix} 0 & 1 & 4 & 2 \\ 0 & 0 & 0 & 0 \\ 0 & 0 & 0 & 1 \\ 0 & 1 & 0 & 0 \end{matrix}$	1 1 1 1	$(\varnothing, \square, \square), (\square, \square, \varnothing)$	256	18
118	$\begin{matrix} 0 & 0 & 0 & 3 \\ 0 & 0 & 1 & 0 \\ 0 & 0 & 0 & 0 \\ 0 & 2 & 3 & 0 \end{matrix}$	1 1 1 1	$(\varnothing, \square, \varnothing), (\varnothing, \square, \varnothing)$	256	15
119	$\begin{matrix} 0 & 2 & 5 \\ 0 & 0 & 0 \\ 0 & 1 & 0 \end{matrix}$	1 1 1	$(\varnothing, \square\square), (\square\square, \varnothing)$	260	16
120	$\begin{matrix} 0 & 1 & 2 & 4 \\ 0 & 0 & 1 & 0 \\ 0 & 0 & 0 & 0 \\ 0 & 1 & 0 & 0 \end{matrix}$	1 1 1 1	$(\varnothing, \square, \square), (\varnothing, \square, \square)$	229	19
121	$\begin{matrix} 0 & 1 & 2 & 4 \\ 0 & 0 & 0 & 0 \\ 0 & 0 & 0 & 0 \\ 0 & 1 & 1 & 0 \end{matrix}$	1 1 1 1	$(\varnothing, \square\square, \varnothing), (\square, \varnothing, \square)$	230	20

Continued on next page.

Continued from previous page.

Period ID	Adjacency matrix	Dimension vector	Generalized partitions	Degree	Euler Number
122	0 1 2 4<br>0 0 0 0<br>0 0 0 0<br>0 1 1 0	1 1 1 1	$(\varnothing,\square,\square),(\square,\square,\varnothing)$	213	19
123	0 3 4<br>0 0 0<br>0 1 0	1 1 1	$(\square,\square),(\square\square,\varnothing)$	196	16
124	0 5 4<br>0 0 0<br>0 0 0	1 1 2	$(\square,\varnothing),(\square,\square),\left(\square,\begin{smallmatrix}\square\\\square\end{smallmatrix}\right)$	240	13
125	0 0 0 4<br>0 0 0 0<br>0 0 0 0<br>0 1 2 0	1 1 1 2	$(\varnothing,\square,\varnothing),(\varnothing,\square,\varnothing),(\varnothing,\square,\varnothing),(\square,\varnothing,\varnothing)$	211	15
126	0 1 4<br>0 0 0<br>0 1 0	1 1 2	$\left(\varnothing,\begin{smallmatrix}\square\\\square\end{smallmatrix}\right),\left(\square,\begin{smallmatrix}\square\\\square\end{smallmatrix}\right)$	224	13
127	0 1 5<br>0 0 0<br>0 1 0	1 1 1	$(\varnothing,\square\square\square)$	240	-12
128	0 2 4<br>0 0 0<br>0 1 0	1 1 1	$(\square,\square\square)$	224	20
129	0 1 1 4<br>0 0 1 0<br>0 0 0 0<br>0 1 0 0	1 1 1 1	$(\varnothing,\square,\square\square)$	202	24
130	0 1 1 4<br>0 0 0 0<br>0 0 0 0<br>0 1 1 0	1 1 1 1	$(\square,\square,\square)$	180	32
131	0 2 4<br>0 0 0<br>0 1 0	1 1 1	$(\square\square,\square)$	163	31
132	0 0 4<br>0 0 0<br>0 6 0	1 1 1	$(\square,\blacksquare),(\square,\varnothing),(\square,\varnothing),(\square,\varnothing)$	192	18
133	0 1 1 3<br>0 0 0 0<br>0 0 0 0<br>0 1 1 0	1 1 1 1		464	12

Continued on next page.

Continued from previous page.

Period ID	Adjacency matrix	Dimension vector	Generalized partitions	Degree	Euler Number
134	0 1 2 3<br>0 0 0 0<br>0 0 0 0<br>0 1 0 0	1 1 1 1		448	12
135	0 0 0 0 2<br>0 0 1 1 0<br>0 0 0 0 0<br>0 0 0 0 0<br>0 3 1 2 0	1 1 1 1 1	$(\varnothing, \varnothing, \square, \blacksquare), (\varnothing, \square, \varnothing, \varnothing)$	384	16
136	0 5 4<br>0 0 0<br>0 0 0	1 1 2	$\left(\varnothing, \begin{smallmatrix}\square\\\square\end{smallmatrix}\right), (\square, \varnothing), (\square, \square)$	384	8
137	0 3 3<br>0 0 1<br>0 0 0	1 1 1	$(\varnothing, \square\square)$	352	10
138	0 1 2 3<br>0 0 0 0<br>0 0 0 0<br>0 1 1 0	1 1 1 1	$(\varnothing, \square\square, \varnothing)$	320	16
139	0 0 4<br>0 0 0<br>0 4 0	1 1 1	$(\square, \varnothing), (\square, \varnothing)$	320	12
140	0 0 4<br>0 0 0<br>0 4 0	1 1 1	$(\varnothing, \square\square), (\square, \blacksquare)$	432	12
141	0 0 0 3<br>0 0 1 0<br>0 0 0 0<br>0 3 2 0	1 1 1 1	$(\varnothing, \square, \blacksquare), (\square, \varnothing, \varnothing)$	400	12
142	0 3 3<br>0 0 1<br>0 0 0	1 1 2	$\left(\varnothing, \begin{smallmatrix}\square\\\square\end{smallmatrix}\right), \left(\varnothing, \begin{smallmatrix}\square\\\square\end{smallmatrix}\right)$	352	10
143	0 0 0 0 3<br>0 0 0 0 0<br>0 0 0 1 0<br>0 0 0 0 0<br>0 2 3 1 0	1 1 1 1 1	$(\varnothing, \varnothing, \varnothing, \square\square), (\square, \square, \varnothing, \blacksquare\blacksquare)$	400	16
144	0 3 3 2 4<br>0 0 0 0 0<br>0 0 0 1 0<br>0 0 0 0 0<br>0 0 0 0 0	1 1 1 2 2	$(\varnothing, \varnothing, \square, \square), (\square, \varnothing, \varnothing, \square)$	369	17

Continued on next page.

Continued from previous page.

Period ID	Adjacency matrix	Dimension vector	Generalized partitions	Degree	Euler Number
145	$\begin{matrix} 0 & 1 & 0 & 4 \\ 0 & 0 & 1 & 0 \\ 0 & 0 & 0 & 0 \\ 0 & 1 & 1 & 0 \end{matrix}$	1 1 2 2	$(\varnothing,\square,\varnothing),(\varnothing,\square,\varnothing)$	353	14
146	$\begin{matrix} 0 & 0 & 0 & 0 & 3 \\ 0 & 0 & 0 & 0 & 0 \\ 0 & 0 & 0 & 1 & 0 \\ 0 & 0 & 0 & 0 & 0 \\ 0 & 2 & 3 & 1 & 0 \end{matrix}$	1 1 1 1 1	$(\varnothing,\varnothing,\varnothing,\square\square),(\square,\varnothing,\square,\blacksquare\blacksquare)$	368	16
147	$\begin{matrix} 0 & 0 & 0 & 0 & 2 \\ 0 & 0 & 0 & 1 & 0 \\ 0 & 0 & 0 & 0 & 0 \\ 0 & 0 & 1 & 0 & 0 \\ 0 & 3 & 2 & 1 & 0 \end{matrix}$	1 1 1 1 1	$(\varnothing,\square,\varnothing,\blacksquare),(\varnothing,\square,\varnothing,\varnothing)$	367	15
148	$\begin{matrix} 0 & 0 & 3 & 1 & 1 \\ 0 & 0 & 0 & 0 & 0 \\ 0 & 0 & 0 & 1 & 1 \\ 0 & 1 & 0 & 0 & 0 \\ 0 & 1 & 0 & 0 & 0 \end{matrix}$	1 1 1 1 1	$(\square,\varnothing,\varnothing,\varnothing)$	351	15
149	$\begin{matrix} 0 & 0 & 0 & 4 \\ 0 & 0 & 0 & 0 \\ 0 & 0 & 0 & 0 \\ 0 & 2 & 3 & 0 \end{matrix}$	1 1 1 1	$(\varnothing,\square,\varnothing),(\square,\varnothing,\varnothing)$	352	13
150	$\begin{matrix} 0 & 0 & 0 & 0 & 3 \\ 0 & 0 & 0 & 0 & 0 \\ 0 & 0 & 0 & 1 & 0 \\ 0 & 0 & 0 & 0 & 0 \\ 0 & 2 & 3 & 1 & 0 \end{matrix}$	1 1 1 1 1	$(\varnothing,\square,\varnothing,\varnothing),(\square,\varnothing,\varnothing,\varnothing)$	352	16
151	$\begin{matrix} 0 & 0 & 0 & 3 & 1 \\ 0 & 0 & 1 & 0 & 0 \\ 0 & 0 & 0 & 0 & 0 \\ 0 & 0 & 1 & 0 & 1 \\ 0 & 3 & 0 & 0 & 0 \end{matrix}$	1 1 1 1 1	$(\varnothing,\square,\varnothing,\varnothing),(\square,\varnothing,\varnothing,\blacksquare)$	331	17
152	$\begin{matrix} 0 & 0 & 3 & 4 \\ 0 & 0 & 0 & 0 \\ 0 & 1 & 0 & 0 \\ 0 & 1 & 0 & 0 \end{matrix}$	1 1 1 1	$(\varnothing,\varnothing,\square\square),(\square,\varnothing,\varnothing)$	326	16
153	$\begin{matrix} 0 & 1 & 3 & 2 \\ 0 & 0 & 0 & 0 \\ 0 & 0 & 0 & 1 \\ 0 & 1 & 0 & 0 \end{matrix}$	1 1 1 1	$(\square,\varnothing,\square)$	319	13
154	$\begin{matrix} 0 & 0 & 0 & 4 \\ 0 & 0 & 4 & 0 \\ 0 & 0 & 0 & 0 \\ 0 & 1 & 0 & 0 \end{matrix}$	1 1 1 2	$(\blacksquare,\square,\square),\left(\varnothing,\varnothing,\begin{smallmatrix}\square\\\square\end{smallmatrix}\right),(\square,\varnothing,\varnothing)$	320	12

Continued on next page.

Continued from previous page.

Period ID	Adjacency matrix	Dimension vector	Generalized partitions	Degree	Euler Number
155	0 0 0 0 0 2<br>0 0 0 0 0 0<br>0 0 0 1 1 0<br>0 0 0 0 0 0<br>0 0 0 0 0 0<br>0 2 3 1 2 0	1 1 1 1 1 1	(∅, ∅, ∅, □, ■),(∅, ∅, ∅, □, ∅),(□, ∅, □, ∅, ■■)	310	21
156	0 1 1 1 3<br>0 0 0 0 0<br>0 0 0 1 0<br>0 0 0 0 0<br>0 1 1 0 0	1 1 1 1 1	(□, ∅, □, ∅)	303	17
157	0 0 0 4<br>0 0 1 0<br>0 0 0 0<br>0 3 1 0	1 1 1 1	(∅, ∅, □□),(□, ∅, ∅)	304	16
158	0 1 5<br>0 0 0<br>0 1 0	1 2 2	(□, □)	274	16
159	0 0 0 4<br>0 0 1 0<br>0 0 0 0<br>0 3 1 0	1 1 1 1	(∅, □, ∅),(□, ∅, ∅)	288	15
160	0 0 0 3<br>0 0 1 0<br>0 0 0 0<br>0 3 2 0	1 1 1 1	(∅, □, ■),(□, ∅, □)	256	12
161	0 0 1 4<br>0 0 0 0<br>0 0 0 0<br>0 3 1 0	1 1 1 1	(∅, ∅, □□),(□□, ∅, ■■)	416	16
162	0 0 0 3 3<br>0 0 0 0 0<br>0 0 0 0 0<br>0 0 0 0 0<br>0 2 2 0 0	1 1 1 1 1	(∅, □, ∅, ∅),(□, ∅, □, ■)	384	16
163	0 0 0 3<br>0 0 1 0<br>0 0 0 0<br>0 3 2 0	1 1 1 1	(∅, □, ■),(□, □, ■■)	378	15
164	0 0 0 3<br>0 0 1 0<br>0 0 0 0<br>0 3 2 0	1 1 1 1	(∅, ∅, □□),(□, □, ■■)	368	12

Continued on next page.

Continued from previous page.

Period ID	Adjacency matrix	Dimension vector	Generalized partitions	Degree	Euler Number
165	0 0 0 3<br>0 0 0 0<br>0 0 0 0<br>0 3 3 0	1 1 1 1	(∅, □, ∅),(□, ∅, ∅)	336	12
166	0 0 1 3<br>0 0 1 0<br>0 0 0 0<br>0 4 0 0	1 1 1 1	(□, ∅, ■),(□, □, ■)	335	13
167	0 2 3<br>0 0 0<br>0 2 0	1 1 1	(□□, ∅)	272	16
168	0 0 0 4 0<br>0 0 0 0 0<br>0 0 0 0 0<br>0 0 0 0 2<br>0 2 2 0 0	1 1 1 1 1	(∅, ∅, ∅, □),(□, ∅, □, ■)	352	16
169	0 0 0 1 3<br>0 0 0 0 0<br>0 0 0 1 0<br>0 0 0 0 0<br>0 2 3 0 0	1 1 1 1 1	(∅, □, ∅, ■),(□, ∅, □, ■)	335	16
170	0 0 0 0 3<br>0 0 0 1 0<br>0 0 0 0 0<br>0 0 1 0 0<br>0 3 1 1 0	1 1 1 1 1	(∅, ∅, ∅, □□),(□, □, ∅, ■■)	336	16
171	0 0 0 0 3<br>0 0 0 0 0<br>0 0 0 1 0<br>0 0 0 0 0<br>0 2 3 1 0	1 1 1 1 1	(∅, ∅, □, ∅),(□, □, ∅, ■■)	320	17
172	0 0 0 0 3<br>0 0 0 0 0<br>0 0 0 1 0<br>0 0 0 0 0<br>0 2 3 1 0	1 1 1 1 1	(∅, ∅, □, ∅),(□, ∅, ∅, ∅)	314	16
173	0 3 1 2<br>0 0 0 1<br>0 0 0 0<br>0 0 1 0	1 1 2 2	(∅, $\begin{smallmatrix}\square\\\square\end{smallmatrix}$, ∅),(∅, $\begin{smallmatrix}\square\\\square\end{smallmatrix}$, ∅)	298	13
174	0 0 0 1 2<br>0 0 1 0 0<br>0 0 0 1 0<br>0 0 0 0 0<br>0 3 2 0 0	1 1 1 1 1	(∅, □, ∅, ■),(□, ∅, □, ■)	309	19

Continued on next page.

Continued from previous page.

Period ID	Adjacency matrix	Dimension vector	Generalized partitions	Degree	Euler Number
175	0 0 0 3<br>0 0 0 0<br>0 0 0 0<br>0 2 4 0	1 1 1 1	$(\varnothing,\square,\blacksquare),(\square,\square,\blacksquare)$	288	15
176	0 0 3 3<br>0 0 0 0<br>0 0 0 1<br>0 2 0 0	1 1 1 1	$(\varnothing,\varnothing,\square\square),(\square,\square,\blacksquare)$	304	14
177	0 0 1 1 1 2<br>0 0 0 0 0 0<br>0 0 0 0 1 0<br>0 0 0 0 0 0<br>0 0 0 1 0 0<br>0 2 1 0 0 0	1 1 1 1 1 1	$(\square,\varnothing,\square,\varnothing,\blacksquare)$	325	20
178	0 0 4 4<br>0 0 0 0<br>0 0 0 0<br>0 1 0 0	1 1 1 2	$\left(\varnothing,\varnothing,\begin{smallmatrix}\square\\ \square\end{smallmatrix}\right),(\varnothing,\square,\varnothing),(\varnothing,\square,\varnothing),(\square,\square,\varnothing)$	304	12
179	0 0 3 1 3<br>0 0 0 0 0<br>0 0 0 1 0<br>0 1 0 0 0<br>0 1 0 0 0	1 1 1 1 1	$(\varnothing,\square,\varnothing,\square),(\square,\varnothing,\varnothing,\varnothing)$	304	16
180	0 0 0 0 2<br>0 0 0 1 0<br>0 0 0 0 0<br>0 0 0 0 0<br>0 2 3 2 0	1 1 1 1 1	$(\varnothing,\square,\varnothing,\varnothing),(\varnothing,\square,\square,\blacksquare\blacksquare)$	304	17
181	0 0 0 3<br>0 0 1 0<br>0 0 0 0<br>0 3 2 0	1 1 1 1	$(\varnothing,\varnothing,\square\square),(\varnothing,\square\square,\blacksquare\blacksquare)$	304	16
182	0 0 5 2<br>0 0 0 0<br>0 3 0 1<br>0 0 0 0	1 1 1 2	$(\varnothing,\square,\square),(\square,\blacksquare,\square)$	273	15
183	0 0 0 3 2<br>0 0 0 0 0<br>0 0 0 0 0<br>0 0 0 0 1<br>0 2 2 0 0	1 1 1 1 1	$(\varnothing,\square,\varnothing,\varnothing),(\square,\varnothing,\varnothing,\varnothing)$	293	16
184	0 0 3 3<br>0 0 0 0<br>0 1 0 0<br>0 1 0 0	1 1 1 1	$(\square,\varnothing,\square)$	272	13

Continued on next page.

Continued from previous page.

Period ID	Adjacency matrix	Dimension vector	Generalized partitions	Degree	Euler Number
185	0 0 0 1 3<br>0 0 0 0 0<br>0 0 0 0 0<br>0 0 1 0 0<br>0 3 1 1 0	1 1 1 1 1	(□, ∅, ∅, ■),(□, □, ∅, ■)	277	17
186	0 0 3 3<br>0 0 0 0<br>0 1 0 1<br>0 1 0 0	1 1 1 1	(∅, ∅, □□),(□, ∅, ∅)	252	16
187	0 0 0 0 3<br>0 0 0 1 0<br>0 0 0 0 0<br>0 0 1 0 0<br>0 3 1 1 0	1 1 1 1 1	(∅, □, ∅, ∅),(□, ∅, ∅, ∅)	277	17
188	0 0 4 4<br>0 0 0 0<br>0 0 0 0<br>0 1 0 0	1 1 1 2	(∅, ∅, $\begin{smallmatrix}\square\\ \square\end{smallmatrix}$),(∅, □, ∅),(∅, □, ∅),(∅, □, $\begin{smallmatrix}\square\\ \square\end{smallmatrix}$)	272	12
189	0 0 0 1 3<br>0 0 1 0 0<br>0 0 0 0 0<br>0 0 0 0 0<br>0 3 1 1 0	1 1 1 1 1	(∅, □, ∅, ∅),(□, ∅, □, ■)	262	19
190	0 0 3 3<br>0 0 0 0<br>0 0 0 1<br>0 1 0 0	1 1 1 2	(∅, ∅, $\begin{smallmatrix}\square\\ \square\end{smallmatrix}$),(∅, ∅, $\begin{smallmatrix}\square\\ \square\end{smallmatrix}$),(□, ∅, ∅)	256	14
191	0 3 3 1<br>0 0 0 1<br>0 0 0 1<br>0 0 0 0	1 1 1 2	(∅, ∅, $\begin{smallmatrix}\square\\ \square\end{smallmatrix}$),(∅, ∅, $\begin{smallmatrix}\square\\ \square\end{smallmatrix}$)	241	17
192	0 0 0 0 3<br>0 0 1 1 0<br>0 0 0 0 0<br>0 0 0 0 0<br>0 3 1 1 0	1 1 1 1 1	(∅, ∅, □, ∅),(∅, □, ∅, ∅)	256	18
193	0 0 0 4<br>0 0 0 0<br>0 0 0 0<br>0 3 4 0	1 1 1 1	(∅, □, ■),(∅, □, ∅),(□, ∅, ∅),(□, □, ■■)	256	14
194	0 0 1 2 3<br>0 0 0 0 0<br>0 0 0 0 0<br>0 0 0 0 0<br>0 2 1 1 0	1 1 1 1 1	(∅, ∅, □□, ∅),(□, □, ∅, ■)	252	22

Continued on next page.

Continued from previous page.

Period ID	Adjacency matrix	Dimension vector	Generalized partitions	Degree	Euler Number
195	0 3 0<br>0 0 5<br>0 0 0	1 1 2	$(\blacksquare\blacksquare, \substack{\square\\\square}), (\blacksquare\blacksquare, \substack{\square\\\square}), (\varnothing, \square)$	288	10
196	0 0 0 4<br>0 0 0 0<br>0 0 0 0<br>0 2 3 0	1 1 1 1	$(\varnothing, \square, \varnothing), (\varnothing, \square, \varnothing)$	304	12
197	0 1 3 1<br>0 0 0 0<br>0 0 0 2<br>0 1 0 0	1 1 1 1	$(\square, \varnothing, \square)$	255	19
198	0 0 1 1 4<br>0 0 0 0 0<br>0 0 0 1 0<br>0 0 0 0 0<br>0 2 1 0 0	1 1 1 1 1	$(\varnothing, \varnothing, \varnothing, \square\square), (\square, \varnothing, \square, \blacksquare)$	330	20
199	0 0 1 3 2<br>0 0 0 0 0<br>0 0 0 0 0<br>0 0 0 0 1<br>0 2 1 0 0	1 1 1 1 1	$(\varnothing, \varnothing, \square, \square), (\square, \square, \varnothing, \blacksquare)$	288	17
200	0 0 2 2 1 1<br>0 0 0 0 0 0<br>0 0 0 0 0 1<br>0 0 0 0 1 0<br>0 1 0 0 0 0<br>0 1 0 0 0 0	1 1 1 1 1 1	$(\square, \varnothing, \varnothing, \varnothing, \varnothing)$	299	20
201	0 0 0 0 4<br>0 0 0 0 0<br>0 0 0 3 0<br>0 0 0 0 0<br>0 1 1 0 0	1 1 1 1 2	$(\varnothing, \blacksquare, \square, \square), (\varnothing, \square, \varnothing, \varnothing), (\square, \varnothing, \varnothing, \varnothing)$	278	16
202	0 0 4 1<br>0 0 0 0<br>0 0 0 1<br>0 1 0 0	1 1 2 2	$(\varnothing, \varnothing, \substack{\square\\\square}), (\varnothing, \substack{\square\\\square}, \varnothing), (\square, \varnothing, \varnothing)$	267	13
203	0 0 0 1 2<br>0 0 1 1 0<br>0 0 0 0 0<br>0 0 0 0 0<br>0 3 2 0 0	1 1 1 1 1	$(\varnothing, \square, \varnothing, \blacksquare), (\varnothing, \square, \square, \blacksquare)$	271	19

Continued on next page.

Continued from previous page.

Period ID	Adjacency matrix	Dimension vector	Generalized partitions	Degree	Euler Number
204	$\begin{smallmatrix} 0 & 0 & 2 & 3 & 2 \\ 0 & 0 & 0 & 0 & 0 \\ 0 & 0 & 0 & 0 & 0 \\ 0 & 3 & 0 & 0 & 1 \\ 0 & 0 & 0 & 0 & 0 \end{smallmatrix}$	1 1 1 1 2	$\left(\varnothing, \square, \varnothing, \substack{\square\\\square}\right), (\square, \varnothing, \blacksquare, \square)$	257	19
205	$\begin{smallmatrix} 0 & 0 & 1 & 2 & 2 & 0 \\ 0 & 0 & 0 & 0 & 0 & 0 \\ 0 & 0 & 0 & 0 & 0 & 0 \\ 0 & 0 & 0 & 0 & 1 & 3 \\ 0 & 0 & 1 & 0 & 0 & 0 \\ 0 & 2 & 0 & 0 & 0 & 0 \end{smallmatrix}$	1 1 1 1 1 1	$(\varnothing, \varnothing, \blacksquare, \varnothing, \square), (\varnothing, \varnothing, \blacksquare, \square, \square), (\square, \square, \varnothing, \varnothing, \blacksquare)$	283	21
206	$\begin{smallmatrix} 0 & 0 & 1 & 3 & 2 \\ 0 & 0 & 0 & 0 & 0 \\ 0 & 0 & 0 & 0 & 0 \\ 0 & 0 & 0 & 0 & 1 \\ 0 & 2 & 1 & 0 & 0 \end{smallmatrix}$	1 1 1 1 1	$(\varnothing, \square, \varnothing, \square), (\square, \varnothing, \square, \blacksquare)$	277	18
207	$\begin{smallmatrix} 0 & 0 & 2 & 2 & 3 \\ 0 & 0 & 0 & 0 & 0 \\ 0 & 0 & 0 & 1 & 0 \\ 0 & 1 & 0 & 0 & 0 \\ 0 & 1 & 0 & 0 & 0 \end{smallmatrix}$	1 1 1 1 1	$(\varnothing, \varnothing, \square, \square), (\square, \varnothing, \varnothing, \varnothing)$	262	18
208	$\begin{smallmatrix} 0 & 0 & 0 & 0 & 3 \\ 0 & 0 & 0 & 1 & 0 \\ 0 & 0 & 0 & 1 & 0 \\ 0 & 0 & 0 & 0 & 0 \\ 0 & 3 & 3 & 1 & 0 \end{smallmatrix}$	1 1 1 1 1	$(\varnothing, \varnothing, \square, \blacksquare), (\varnothing, \varnothing, \square, \blacksquare), (\varnothing, \square, \varnothing, \varnothing), (\square, \varnothing, \varnothing, \varnothing)$	257	17
209	$\begin{smallmatrix} 0 & 0 & 2 & 1 & 3 \\ 0 & 0 & 0 & 0 & 0 \\ 0 & 0 & 0 & 1 & 0 \\ 0 & 1 & 0 & 0 & 0 \\ 0 & 2 & 0 & 0 & 0 \end{smallmatrix}$	1 1 1 1 1	$(\square, \varnothing, \varnothing, \varnothing), (\square, \varnothing, \varnothing, \varnothing)$	257	17
210	$\begin{smallmatrix} 0 & 0 & 1 & 2 & 3 \\ 0 & 0 & 0 & 0 & 0 \\ 0 & 0 & 0 & 1 & 0 \\ 0 & 0 & 0 & 0 & 0 \\ 0 & 2 & 1 & 0 & 0 \end{smallmatrix}$	1 1 1 1 1	$(\varnothing, \varnothing, \square, \square), (\square, \varnothing, \square, \blacksquare)$	256	18
211	$\begin{smallmatrix} 0 & 0 & 0 & 5 \\ 0 & 0 & 0 & 0 \\ 0 & 0 & 0 & 0 \\ 0 & 1 & 1 & 0 \end{smallmatrix}$	1 1 1 2	$\left(\varnothing, \varnothing, \substack{\square\\\square}\right), \left(\varnothing, \varnothing, \substack{\square\\\square}\right), (\varnothing, \square, \varnothing), (\square, \varnothing, \varnothing)$	241	15
212	$\begin{smallmatrix} 0 & 1 & 3 & 2 \\ 0 & 0 & 0 & 0 \\ 0 & 0 & 0 & 1 \\ 0 & 1 & 0 & 0 \end{smallmatrix}$	1 1 1 2	$(\square, \varnothing, \square)$	235	15

Continued on next page.

Continued from previous page.

Period ID	Adjacency matrix	Dimension vector	Generalized partitions	Degree	Euler Number
213	$\begin{matrix}0&0&0&4\\0&0&1&0\\0&0&0&0\\0&2&2&0\end{matrix}$	1 1 1 1	$(\varnothing,\varnothing,\square\square),(\varnothing,\square,\varnothing)$	256	18
214	$\begin{matrix}0&0&0&4\\0&0&1&0\\0&0&0&0\\0&2&2&0\end{matrix}$	1 1 1 1	$(\varnothing,\square,\varnothing),(\varnothing,\square,\varnothing)$	240	16
215	$\begin{matrix}0&0&1&2&3\\0&0&0&0&0\\0&0&0&0&0\\0&0&0&0&0\\0&2&1&1&0\end{matrix}$	1 1 1 1 1	$(\varnothing,\square,\square,\varnothing),(\square,\varnothing,\square,\blacksquare)$	235	19
216	$\begin{matrix}0&0&2&3\\0&0&0&0\\0&1&0&0\\0&1&1&0\end{matrix}$	1 1 1 1	$(\square,\square,\varnothing)$	219	19
217	$\begin{matrix}0&0&3&4\\0&0&0&0\\0&1&0&0\\0&2&0&0\end{matrix}$	1 1 1 1	$(\varnothing,\square,\square),(\square,\varnothing,\varnothing),(\square,\varnothing,\varnothing)$	225	16
218	$\begin{matrix}0&0&0&0&2\\0&0&0&1&0\\0&0&0&0&0\\0&0&1&0&0\\0&3&2&1&0\end{matrix}$	1 1 1 1 1	$(\varnothing,\square,\varnothing,\blacksquare),(\square,\square,\varnothing,\blacksquare)$	229	21
219	$\begin{matrix}0&0&5\\0&0&0\\0&1&0\end{matrix}$	1 1 3	$\left(\varnothing,\begin{smallmatrix}\square\\\square\\\square\end{smallmatrix}\right),\left(\varnothing,\begin{smallmatrix}\square\\\square\\\square\end{smallmatrix}\right),\left(\varnothing,\begin{smallmatrix}\square\\\square\\\square\end{smallmatrix}\right),(\square,\varnothing)$	224	9
220	$\begin{matrix}0&0&3&3\\0&0&0&0\\0&2&0&0\\0&2&0&0\end{matrix}$	1 1 1 1	$(\square,\varnothing,\varnothing),(\square,\varnothing,\varnothing),(\square,\varnothing,\varnothing)$	195	18
221	$\begin{matrix}0&3&4\\0&0&0\\0&1&0\end{matrix}$	1 1 1	$(\varnothing,\square\square),(\square\square,\varnothing)$	288	16
222	$\begin{matrix}0&3&4\\0&0&1\\0&0&0\end{matrix}$	1 1 1	$(\varnothing,\square\square),(\square,\square)$	240	16
223	$\begin{matrix}0&0&0&2\\0&0&1&0\\0&0&0&0\\0&2&4&0\end{matrix}$	1 1 1 1	$(\varnothing,\square,\varnothing),(\varnothing,\square\square,\blacksquare\blacksquare)$	256	16

Continued on next page.

Continued from previous page.

Period ID	Adjacency matrix	Dimension vector	Generalized partitions	Degree	Euler Number
224	$\begin{matrix}0&3&1&4\\0&0&0&0\\0&0&0&0\\0&0&1&0\end{matrix}$	1 1 2 2	$\left(\varnothing,\begin{smallmatrix}\square\\\square\end{smallmatrix},\varnothing\right),\left(\varnothing,\begin{smallmatrix}\square\\\square\end{smallmatrix},\varnothing\right),(\square,\varnothing,\square)$	251	14
225	$\begin{matrix}0&1&3&3\\0&0&0&0\\0&0&0&1\\0&1&0&0\end{matrix}$	1 1 1 1	$(\varnothing,\varnothing,\square\square),(\square,\square,\varnothing)$	230	18
226	$\begin{matrix}0&3&5&2\\0&0&0&1\\0&0&0&0\\0&0&0&0\end{matrix}$	1 1 1 2	$(\varnothing,\square,\square),(\varnothing,\square,\square)$	225	18
227	$\begin{matrix}0&0&0&0&4\\0&0&0&0&0\\0&0&0&3&0\\0&0&0&0&0\\0&1&1&0&0\end{matrix}$	1 1 1 1 2	$(\varnothing,\blacksquare,\square,\varnothing),\left(\varnothing,\blacksquare,\square,\begin{smallmatrix}\square\\\square\end{smallmatrix}\right),(\varnothing,\square,\varnothing,\varnothing),(\square,\varnothing,\varnothing,\varnothing)$	236	19
228	$\begin{matrix}0&0&4&4\\0&0&0&0\\0&1&0&0\\0&1&0&0\end{matrix}$	1 1 1 1	$(\varnothing,\square,\square),(\varnothing,\square,\square),(\square,\varnothing,\varnothing)$	220	18
229	$\begin{matrix}0&0&0&0&3\\0&0&0&0&0\\0&0&0&1&0\\0&0&0&0&0\\0&2&4&2&0\end{matrix}$	1 1 1 1 1	$(\varnothing,\varnothing,\square,\blacksquare),(\varnothing,\square,\varnothing,\varnothing),(\varnothing,\square,\varnothing,\varnothing),(\square,\varnothing,\square,\blacksquare\blacksquare)$	241	18
230	$\begin{matrix}0&5&4\\0&0&0\\0&0&0\end{matrix}$	1 1 2	$\left(\varnothing,\begin{smallmatrix}\square\\\square\end{smallmatrix}\right),(\square,\square),(\square\square,\varnothing)$	240	8
231	$\begin{matrix}0&3&5&2\\0&0&0&0\\0&0&0&1\\0&0&0&0\end{matrix}$	1 1 1 2	$(\varnothing,\square,\square),(\square,\varnothing,\varnothing),\left(\square,\varnothing,\begin{smallmatrix}\square\\\square\end{smallmatrix}\right)$	225	17
232	$\begin{matrix}0&0&2&4\\0&0&1&0\\0&0&0&0\\0&1&0&0\end{matrix}$	1 1 1 2	$\left(\varnothing,\varnothing,\begin{smallmatrix}\square\\\square\end{smallmatrix}\right),(\varnothing,\square,\square)$	230	14
233	$\begin{matrix}0&0&0&0&3\\0&0&0&1&0\\0&0&0&0&0\\0&0&1&0&0\\0&2&1&2&0\end{matrix}$	1 1 1 1 1	$(\varnothing,\varnothing,\square,\varnothing),(\varnothing,\square,\varnothing,\varnothing)$	235	19

Continued on next page.

Continued from previous page.

Period ID	Adjacency matrix	Dimension vector	Generalized partitions	Degree	Euler Number
234	$\begin{smallmatrix} 0&0&0&3&3\\ 0&0&0&0&0\\ 0&0&0&0&0\\ 0&0&1&0&0\\ 0&2&1&0&0 \end{smallmatrix}$	1 1 1 1 1	$(\varnothing,\square,\varnothing,\varnothing),(\square,\varnothing,\square,\varnothing)$	215	21
235	$\begin{smallmatrix} 0&1&3&4\\ 0&0&0&0\\ 0&0&0&0\\ 0&1&0&0 \end{smallmatrix}$	1 1 2 2	$\left(\varnothing,\square,\begin{smallmatrix}\square\\\square\end{smallmatrix}\right),(\square,\square,\varnothing)$	199	16
236	$\begin{smallmatrix} 0&0&0&4\\ 0&0&0&0\\ 0&0&0&0\\ 0&2&3&0 \end{smallmatrix}$	1 1 1 1	$(\varnothing,\square,\varnothing),(\square,\square,\blacksquare)$	208	18
237	$\begin{smallmatrix} 0&2&2&3\\ 0&0&0&0\\ 0&0&0&0\\ 0&1&1&0 \end{smallmatrix}$	1 1 1 1	$(\varnothing,\square\!\square,\varnothing),(\square\!\square,\varnothing,\varnothing)$	200	24
238	$\begin{smallmatrix} 0&5&4\\ 0&0&0\\ 0&0&0 \end{smallmatrix}$	1 1 2	$\left(\varnothing,\begin{smallmatrix}\square&\square\\\square&\square\end{smallmatrix}\right),(\square,\varnothing),(\square,\square)$	192	0
239	$\begin{smallmatrix} 0&1&4&2\\ 0&0&0&0\\ 0&0&0&1\\ 0&1&0&0 \end{smallmatrix}$	1 1 1 1	$(\varnothing,\square\!\square,\varnothing),(\square,\varnothing,\square)$	266	20
240	$\begin{smallmatrix} 0&1&3&3\\ 0&0&0&0\\ 0&0&0&1\\ 0&1&0&0 \end{smallmatrix}$	1 1 1 1	$(\varnothing,\square,\square),(\square,\varnothing,\square)$	224	19
241	$\begin{smallmatrix} 0&0&0&0&2\\ 0&0&0&1&0\\ 0&0&0&0&0\\ 0&0&1&0&0\\ 0&2&1&3&0 \end{smallmatrix}$	1 1 1 1 1	$(\varnothing,\varnothing,\square,\varnothing),(\varnothing,\square,\square,\blacksquare\!\blacksquare)$	235	20
242	$\begin{smallmatrix} 0&1&5&4\\ 0&0&0&0\\ 0&1&0&0\\ 0&0&0&0 \end{smallmatrix}$	1 1 1 3	$\left(\varnothing,\square,\begin{smallmatrix}\square\\\square\end{smallmatrix}\right),\left(\square,\varnothing,\begin{smallmatrix}\square\\\square\\\square\end{smallmatrix}\right)$	224	16
243	$\begin{smallmatrix} 0&2&2&3\\ 0&0&1&0\\ 0&0&0&0\\ 0&1&0&0 \end{smallmatrix}$	1 1 1 1	$(\varnothing,\square,\square),(\square,\square,\varnothing)$	203	20

Continued on next page.

Continued from previous page.

Period ID	Adjacency matrix	Dimension vector	Generalized partitions	Degree	Euler Number
244	$\begin{smallmatrix}0&0&1&4\\0&0&1&0\\0&0&0&0\\0&1&0&0\end{smallmatrix}$	1 1 1 2	$\left(\varnothing,\varnothing,\begin{smallmatrix}\square\\\square\end{smallmatrix}\right),\left(\varnothing,\square,\begin{smallmatrix}\square\\\square\end{smallmatrix}\right)$	203	17
245	$\begin{smallmatrix}0&0&0&4\\0&0&0&0\\0&0&0&0\\0&2&3&0\end{smallmatrix}$	1 1 1 1	$(\varnothing,\square\square,\blacksquare),(\square,\varnothing,\varnothing)$	208	16
246	$\begin{smallmatrix}0&3&3\\0&0&1\\0&0&0\end{smallmatrix}$	1 1 1	$(\square,\square\square)$	192	30
247	$\begin{smallmatrix}0&0&3\\0&0&0\\0&7&0\end{smallmatrix}$	1 1 1	$(\square,\blacksquare),(\square,\varnothing),(\square,\varnothing),(\square\square,\blacksquare\blacksquare)$	208	16
248	$\begin{smallmatrix}0&0&0&5\\0&0&0&0\\0&0&0&0\\0&2&2&0\end{smallmatrix}$	1 1 1 1	$(\varnothing,\square,\square),(\square,\varnothing,\varnothing)$	208	20
249	$\begin{smallmatrix}0&0&5\\0&0&0\\0&5&0\end{smallmatrix}$	1 1 1	$(\varnothing,\square\square),(\square,\blacksquare),(\square,\varnothing),(\square,\varnothing)$	176	16
250	$\begin{smallmatrix}0&0&1&5\\0&0&0&0\\0&0&0&0\\0&4&1&0\end{smallmatrix}$	1 1 1 1	$(\varnothing,\square,\square),(\square,\varnothing,\blacksquare),(\square,\varnothing,\varnothing),(\square,\varnothing,\varnothing)$	193	19
251	$\begin{smallmatrix}0&1&3&2\\0&0&0&0\\0&0&0&1\\0&1&0&0\end{smallmatrix}$	1 1 1 1	$(\square,\square,\square)$	176	33
252	$\begin{smallmatrix}0&0&0&4\\0&0&1&0\\0&0&0&0\\0&4&2&0\end{smallmatrix}$	1 1 1 1	$(\varnothing,\square,\blacksquare),(\varnothing,\square,\varnothing),(\square,\varnothing,\varnothing),(\square,\varnothing,\varnothing)$	177	21
253	$\begin{smallmatrix}0&5&3\\0&0&1\\0&0&0\end{smallmatrix}$	1 1 2	$\left(\varnothing,\begin{smallmatrix}\square\\\square\end{smallmatrix}\right),\left(\varnothing,\begin{smallmatrix}\square\\\square\end{smallmatrix}\right),(\square,\square)$	177	17
254	$\begin{smallmatrix}0&0&5\\0&0&0\\0&3&0\end{smallmatrix}$	1 1 1	$(\square,\varnothing),(\square,\square)$	160	30
255	$\begin{smallmatrix}0&0&4\\0&0&0\\0&4&0\end{smallmatrix}$	1 1 1	$(\square,\varnothing),(\square,\square)$	144	23

Continued on next page.

Continued from previous page.

Period ID	Adjacency matrix	Dimension vector	Generalized partitions	Degree	Euler Number
256	0 3 3 3 0<br>0 0 0 0 1<br>0 0 0 0 1<br>0 0 0 0 1<br>0 0 0 0 0	1 1 1 1 2	$(\varnothing,\varnothing,\varnothing,\square),(\varnothing,\varnothing,\varnothing,\square)$	385	17
257	0 0 0 3<br>0 0 0 0<br>0 0 0 0<br>0 2 4 0	1 1 1 1	$(\varnothing,\square,\blacksquare),(\varnothing,\square,\varnothing)$	384	12
258	0 0 5<br>0 0 0<br>0 3 0	1 1 1	$(\varnothing,\square\square),(\square,\varnothing)$	320	10
259	0 0 4<br>0 0 0<br>0 4 0	1 1 1	$(\square,\blacksquare),(\square,\square)$	256	16
260	0 0 0 4<br>0 0 0 0<br>0 0 0 0<br>0 2 3 0	1 1 1 1	$(\varnothing,\varnothing,\square\square),(\square,\square,\blacksquare\blacksquare)$	384	16
261	0 0 0 3 3<br>0 0 0 0 0<br>0 0 0 0 0<br>0 0 0 0 0<br>0 2 2 0 0	1 1 1 1 1	$(\varnothing,\varnothing,\square,\square),(\square,\varnothing,\varnothing,\varnothing)$	336	16
262	0 0 0 0 3<br>0 0 0 0 0<br>0 0 0 1 0<br>0 0 0 0 0<br>0 2 3 1 0	1 1 1 1 1	$(\varnothing,\square,\varnothing,\varnothing),(\square,\varnothing,\square,\blacksquare\blacksquare)$	320	16
263	0 0 1 4<br>0 0 0 0<br>0 0 0 0<br>0 3 1 0	1 1 1 1	$(\varnothing,\varnothing,\square\square),(\square,\square,\blacksquare)$	304	18
264	0 0 1 4<br>0 0 0 0<br>0 0 0 0<br>0 3 1 0	1 1 1 1	$(\varnothing,\square,\square),(\square,\varnothing,\varnothing)$	288	13
265	0 3 0 4<br>0 0 1 0<br>0 0 0 0<br>0 0 1 0	1 1 2 2	$\left(\varnothing,\varnothing,\begin{smallmatrix}\square\\\square\end{smallmatrix}\right),(\varnothing,\square,\varnothing),\left(\varnothing,\begin{smallmatrix}\square\\\square\end{smallmatrix},\varnothing\right)$	273	14

Continued on next page.

Continued from previous page.

Period ID	Adjacency matrix	Dimension vector	Generalized partitions	Degree	Euler Number
266	$\begin{matrix} 0 & 0 & 1 & 4 \\ 0 & 0 & 1 & 0 \\ 0 & 0 & 0 & 0 \\ 0 & 3 & 0 & 0 \end{matrix}$	1 1 1 1	$(\varnothing,\square,\square),(\square,\varnothing,\varnothing)$	250	19
267	$\begin{matrix} 0 & 0 & 0 & 0 & 3 \\ 0 & 0 & 0 & 0 & 0 \\ 0 & 0 & 0 & 1 & 0 \\ 0 & 0 & 0 & 0 & 0 \\ 0 & 2 & 3 & 1 & 0 \end{matrix}$	1 1 1 1 1	$(\varnothing,\square,\square,\blacksquare\blacksquare),(\square,\varnothing,\varnothing,\varnothing)$	336	20
268	$\begin{matrix} 0 & 0 & 0 & 5 \\ 0 & 0 & 0 & 0 \\ 0 & 0 & 0 & 0 \\ 0 & 2 & 2 & 0 \end{matrix}$	1 1 1 1	$(\varnothing,\varnothing,\square\square),(\square,\varnothing,\varnothing)$	320	12
269	$\begin{matrix} 0 & 0 & 1 & 2 & 3 & 0 \\ 0 & 0 & 0 & 0 & 0 & 0 \\ 0 & 0 & 0 & 0 & 0 & 0 \\ 0 & 0 & 0 & 0 & 0 & 0 \\ 0 & 0 & 1 & 0 & 0 & 3 \\ 0 & 2 & 0 & 0 & 0 & 0 \end{matrix}$	1 1 1 1 1 1	$(\varnothing,\varnothing,\varnothing,\blacksquare\blacksquare,\square\square),(\varnothing,\varnothing,\square,\square,\varnothing),(\square,\square,\varnothing,\varnothing,\blacksquare)$	320	20
270	$\begin{matrix} 0 & 0 & 0 & 3 & 3 \\ 0 & 0 & 0 & 0 & 0 \\ 0 & 0 & 0 & 0 & 0 \\ 0 & 0 & 1 & 0 & 0 \\ 0 & 2 & 1 & 0 & 0 \end{matrix}$	1 1 1 1 1	$(\varnothing,\varnothing,\square,\square),(\varnothing,\square,\varnothing,\varnothing)$	304	16
271	$\begin{matrix} 0 & 0 & 0 & 1 & 3 \\ 0 & 0 & 0 & 0 & 0 \\ 0 & 0 & 0 & 1 & 0 \\ 0 & 0 & 0 & 0 & 0 \\ 0 & 2 & 3 & 0 & 0 \end{matrix}$	1 1 1 1 1	$(\varnothing,\square,\square,\blacksquare),(\square,\varnothing,\varnothing,\varnothing)$	293	18
272	$\begin{matrix} 0 & 0 & 0 & 0 & 3 \\ 0 & 0 & 0 & 1 & 0 \\ 0 & 0 & 0 & 0 & 0 \\ 0 & 0 & 0 & 0 & 0 \\ 0 & 2 & 3 & 1 & 0 \end{matrix}$	1 1 1 1 1	$(\varnothing,\square,\varnothing,\varnothing),(\varnothing,\square,\square,\blacksquare\blacksquare)$	288	16
273	$\begin{matrix} 0 & 0 & 0 & 1 & 3 \\ 0 & 0 & 0 & 0 & 0 \\ 0 & 0 & 0 & 1 & 0 \\ 0 & 0 & 0 & 0 & 0 \\ 0 & 2 & 3 & 0 & 0 \end{matrix}$	1 1 1 1 1	$(\varnothing,\square,\varnothing,\varnothing),(\square,\varnothing,\square,\blacksquare)$	282	18
274	$\begin{matrix} 0 & 0 & 0 & 4 \\ 0 & 0 & 0 & 0 \\ 0 & 0 & 0 & 0 \\ 0 & 2 & 3 & 0 \end{matrix}$	1 1 1 1	$(\varnothing,\varnothing,\square\square),(\varnothing,\square,\varnothing)$	288	16

Continued on next page.

Continued from previous page.

Period ID	Adjacency matrix	Dimension vector	Generalized partitions	Degree	Euler Number
275	0 0 0 5<br>0 0 1 0<br>0 0 0 0<br>0 2 1 0	1 1 1 1	$(\varnothing,\varnothing,\square\square),(\varnothing,\square,\varnothing)$	272	14
276	0 1 2 3<br>0 0 1 0<br>0 0 0 0<br>0 1 0 0	1 1 1 1	$(\varnothing,\square\square,\varnothing)$	286	16
277	0 0 1 2 3 0<br>0 0 0 0 0 0<br>0 0 0 0 0 0<br>0 0 0 0 0 0<br>0 0 1 0 0 3<br>0 2 0 0 0 0	1 1 1 1 1 1	$(\varnothing,\varnothing,\varnothing,\varnothing,\square),(\varnothing,\varnothing,\square,\blacksquare,\square),(\square,\square,\varnothing,\varnothing,\blacksquare)$	278	21
278	0 0 0 0 2<br>0 0 0 1 0<br>0 0 0 0 0<br>0 0 0 0 0<br>0 2 3 2 0	1 1 1 1 1	$(\varnothing,\varnothing,\square,\varnothing),(\varnothing,\square,\square,\blacksquare\blacksquare)$	272	17
279	0 0 0 3<br>0 0 1 0<br>0 0 0 0<br>0 3 2 0	1 1 1 1	$(\varnothing,\square,\varnothing),(\square,\square,\blacksquare\blacksquare)$	256	14
280	0 2 4<br>0 0 0<br>0 1 0	1 2 2	$\left(\varnothing,\begin{smallmatrix}\square\\\square\end{smallmatrix}\right),(\square\square,\varnothing)$	228	20
281	0 0 0 1 3<br>0 0 1 0 0<br>0 0 0 0 0<br>0 0 0 0 0<br>0 3 1 1 0	1 1 1 1 1	$(\varnothing,\square,\square,\blacksquare),(\square,\varnothing,\varnothing,\varnothing)$	256	18
282	0 0 4 2<br>0 0 0 0<br>0 4 0 1<br>0 0 0 0	1 1 1 2	$\left(\varnothing,\square,\begin{smallmatrix}\square\\\square\end{smallmatrix}\right),(\square,\blacksquare,\varnothing),(\square,\blacksquare,\square)$	225	19
283	0 0 0 3<br>0 0 1 0<br>0 0 0 0<br>0 3 2 0	1 1 1 1	$(\varnothing,\square,\blacksquare),(\square,\square,\blacksquare)$	224	19
284	0 0 0 4<br>0 0 0 0<br>0 0 0 0<br>0 2 3 0	1 1 1 1	$(\varnothing,\varnothing,\square\square),(\square,\varnothing,\varnothing)$	324	18

Continued on next page.

Continued from previous page.

Period ID	Adjacency matrix	Dimension vector	Generalized partitions	Degree	Euler Number
285	0 0 0 0 2 2<br>0 0 0 1 0 0<br>0 0 0 0 0 0<br>0 0 1 0 0 0<br>0 0 1 0 0 0<br>0 2 0 1 0 0	1 1 1 1 1 1	$(\varnothing,\square,\varnothing,\varnothing,\varnothing)$	293	21
286	0 0 0 4 1<br>0 0 0 0 0<br>0 0 0 0 0<br>0 0 0 0 1<br>0 2 2 0 0	1 1 1 1 1	$(\varnothing,\varnothing,\square,\square),(\varnothing,\square,\square,\blacksquare)$	288	16
287	0 0 0 3 3<br>0 0 1 0 0<br>0 0 0 0 0<br>0 0 1 0 0<br>0 2 0 0 0	1 1 1 1 1	$(\varnothing,\varnothing,\square,\square),(\varnothing,\square,\varnothing,\varnothing)$	267	17
288	0 3 5<br>0 0 0<br>0 0 0	1 1 2	$\left(\varnothing,\substack{\square\\\square}\right),\left(\varnothing,\substack{\square\\\square}\right),(\square,\varnothing),\left(\square,\substack{\square\\\square}\right)$	240	13
289	0 0 2 1 4<br>0 0 0 0 0<br>0 0 0 1 0<br>0 1 0 0 0<br>0 1 0 0 0	1 1 1 1 1	$(\varnothing,\varnothing,\varnothing,\square\square),(\square,\varnothing,\varnothing,\varnothing)$	278	20
290	0 0 0 0 3<br>0 0 0 0 0<br>0 0 0 1 0<br>0 0 0 0 0<br>0 2 3 1 0	1 1 1 1 1	$(\varnothing,\varnothing,\square,\varnothing),(\varnothing,\square,\varnothing,\varnothing)$	272	16
291	0 0 1 2 3 0<br>0 0 0 0 0 0<br>0 0 0 0 0 0<br>0 0 0 0 0 0<br>0 0 1 0 0 3<br>0 2 0 0 0 0	1 1 1 1 1 1	$(\varnothing,\varnothing,\square,\square,\varnothing),(\varnothing,\square,\varnothing,\blacksquare,\square),(\square,\varnothing,\varnothing,\blacksquare,\varnothing)$	272	21
292	0 0 0 1 2<br>0 0 0 0 0<br>0 0 0 1 0<br>0 0 0 0 0<br>0 2 4 0 0	1 1 1 1 1	$(\varnothing,\square,\square,\blacksquare),(\square,\square,\varnothing,\blacksquare\blacksquare)$	261	20
293	0 0 0 4<br>0 0 1 0<br>0 0 0 0<br>0 3 1 0	1 1 1 1	$(\varnothing,\varnothing,\square\square),(\varnothing,\square,\varnothing)$	260	16

Continued on next page.

Continued from previous page.

Period ID	Adjacency matrix	Dimension vector	Generalized partitions	Degree	Euler Number
294	$\begin{matrix}0&3&1&4\\0&0&0&0\\0&0&0&0\\0&0&1&0\end{matrix}$	1 1 2 2	$\left(\varnothing,\varnothing,\begin{smallmatrix}\square\\\square\end{smallmatrix}\right),\left(\varnothing,\begin{smallmatrix}\square\\\square\end{smallmatrix},\varnothing\right),(\square,\square,\varnothing)$	241	14
295	$\begin{matrix}0&0&3&2\\0&0&0&0\\0&0&0&1\\0&2&0&0\end{matrix}$	1 1 1 2	$(\square,\varnothing,\varnothing),(\square,\varnothing,\varnothing),(\square,\varnothing,\varnothing)$	244	13
296	$\begin{matrix}0&0&0&1&4\\0&0&1&0&0\\0&0&0&0&0\\0&0&0&0&0\\0&2&1&1&0\end{matrix}$	1 1 1 1 1	$(\varnothing,\varnothing,\square,\square),(\varnothing,\square,\varnothing,\varnothing)$	246	18
297	$\begin{matrix}0&0&0&0&4\\0&0&0&0&0\\0&0&0&2&0\\0&0&0&0&0\\0&1&1&0&0\end{matrix}$	1 1 1 1 2	$\left(\varnothing,\varnothing,\varnothing,\begin{smallmatrix}\square\\\square\end{smallmatrix}\right),(\varnothing,\square,\varnothing,\varnothing),(\square,\blacksquare,\square,\varnothing)$	246	17
298	$\begin{matrix}0&0&0&3&3\\0&0&0&0&0\\0&0&0&0&0\\0&0&0&0&0\\0&2&2&0&0\end{matrix}$	1 1 1 1 1	$(\varnothing,\square,\square,\varnothing),(\square,\varnothing,\square,\blacksquare)$	240	19
299	$\begin{matrix}0&0&0&3\\0&0&1&0\\0&0&0&0\\0&3&2&0\end{matrix}$	1 1 1 1	$(\varnothing,\square,\varnothing),(\varnothing,\square,\varnothing)$	239	13
300	$\begin{matrix}0&0&0&0&4\\0&0&0&0&0\\0&0&0&1&0\\0&0&0&0&0\\0&2&3&2&0\end{matrix}$	1 1 1 1 1	$(\varnothing,\varnothing,\varnothing,\square\square),(\varnothing,\varnothing,\square,\blacksquare),(\varnothing,\square,\varnothing,\varnothing),(\square,\varnothing,\square,\blacksquare\blacksquare)$	236	22
301	$\begin{matrix}0&0&4&3\\0&0&0&0\\0&1&0&0\\0&2&0&0\end{matrix}$	1 1 1 1	$(\varnothing,\square\square,\varnothing),(\square,\varnothing,\varnothing),(\square,\varnothing,\varnothing)$	226	18
302	$\begin{matrix}0&0&0&0&3\\0&0&0&0&0\\0&0&0&1&0\\0&0&0&0&0\\0&3&3&2&0\end{matrix}$	1 1 1 1 1	$(\varnothing,\varnothing,\square,\blacksquare),(\varnothing,\square,\varnothing,\varnothing),(\square,\varnothing,\varnothing,\varnothing),(\square,\varnothing,\square,\blacksquare\blacksquare)$	230	18
303	$\begin{matrix}0&0&3&0\\0&0&0&1\\0&3&0&3\\0&0&0&0\end{matrix}$	1 1 1 2	$\left(\varnothing,\blacksquare\blacksquare,\begin{smallmatrix}\square\\\square\end{smallmatrix}\right),\left(\varnothing,\blacksquare\blacksquare,\begin{smallmatrix}\square\\\square\end{smallmatrix}\right),(\varnothing,\varnothing,\square)$	209	16

Continued on next page.

Continued from previous page.

Period ID	Adjacency matrix	Dimension vector	Generalized partitions	Degree	Euler Number
304	0 0 0 4<br>0 0 0 0<br>0 0 0 0<br>0 2 3 0	1 1 1 1	(∅, □□, ■■),(□, ∅, □)	256	8
305	0 0 0 0 2<br>0 0 0 1 0<br>0 0 0 0 0<br>0 0 1 0 0<br>0 2 1 3 0	1 1 1 1 1	(∅, ∅, □□, ■■),(∅, □, ∅, ∅)	246	18
306	0 0 0 1 3<br>0 0 0 1 0<br>0 0 0 0 0<br>0 0 0 0 0<br>0 2 3 0 0	1 1 1 1 1	(∅, □, ∅, ∅),(∅, □, □, ■)	245	19
307	0 0 2 3<br>0 0 1 0<br>0 0 0 0<br>0 3 0 0	1 1 1 1	(∅, □□, ∅),(□, ∅, ■)	238	22
308	0 0 3 5<br>0 0 0 0<br>0 1 0 0<br>0 1 0 0	1 1 1 1	(∅, ∅, □□),(∅, □, □),(□, ∅, ∅)	220	16
309	0 0 0 1 2 2<br>0 0 0 0 0 0<br>0 0 0 0 0 0<br>0 0 1 0 0 0<br>0 0 1 0 0 0<br>0 2 0 1 0 0	1 1 1 1 1 1	(□, □, ∅, ∅, ■)	251	21
310	0 0 0 3 3<br>0 0 0 0 0<br>0 0 0 0 0<br>0 0 1 0 0<br>0 2 1 0 0	1 1 1 1 1	(∅, ∅, □, □),(□, □, ∅, ■)	230	18
311	0 0 1 2 3<br>0 0 0 0 0<br>0 0 0 1 0<br>0 0 0 0 0<br>0 2 1 0 0	1 1 1 1 1	(∅, ∅, □□, ∅),(□, ∅, ∅, ∅)	250	22
312	0 0 5<br>0 0 0<br>0 5 0	1 1 1	(∅, □□),(□, ■),(□, ∅),(□□, ■■)	224	16
313	0 0 0 3<br>0 0 0 0<br>0 0 0 0<br>0 2 4 0	1 1 1 1	(∅, □, ■),(∅, □, □)	240	12

Continued on next page.

Continued from previous page.

Period ID	Adjacency matrix	Dimension vector	Generalized partitions	Degree	Euler Number
314	0 0 0 3 3<br>0 0 0 0 0<br>0 0 0 0 0<br>0 0 1 0 0<br>0 2 1 0 0	1 1 1 1 1	$(\varnothing,\square,\varnothing,\square),(\square,\varnothing,\square,\blacksquare)$	230	18
315	0 0 3 3<br>0 0 0 0<br>0 0 0 1<br>0 2 0 0	1 1 1 1	$(\varnothing,\varnothing,\square\square),(\square,\varnothing,\varnothing)$	218	14
316	0 0 0 4<br>0 0 0 0<br>0 0 0 0<br>0 2 3 0	1 1 1 1	$(\varnothing,\square,\square),(\square,\square,\blacksquare\blacksquare)$	208	20
317	0 0 0 1 3<br>0 0 1 0 0<br>0 0 0 0 0<br>0 0 0 0 0<br>0 2 2 1 0	1 1 1 1 1	$(\varnothing,\square,\varnothing,\varnothing),(\varnothing,\square,\square,\blacksquare)$	214	20
318	0 0 0 3 2<br>0 0 2 0 0<br>0 0 0 0 0<br>0 0 0 0 1<br>0 1 0 0 0	1 1 1 2 2	$(\blacksquare,\square,\varnothing,\varnothing),\left(\varnothing,\varnothing,\varnothing,\begin{smallmatrix}\square\\\square\end{smallmatrix}\right),\left(\varnothing,\varnothing,\varnothing,\begin{smallmatrix}\square\\\square\end{smallmatrix}\right),(\square,\varnothing,\varnothing,\varnothing)$	209	15
319	0 0 0 0 2<br>0 0 0 0 0<br>0 0 0 2 0<br>0 0 0 0 0<br>0 2 3 1 0	1 1 1 1 1	$(\varnothing,\varnothing,\square,\varnothing),(\square,\varnothing,\square,\blacksquare\blacksquare)$	209	20
320	0 3 5 2<br>0 0 0 1<br>0 0 0 0<br>0 0 0 0	1 1 1 2	$(\varnothing,\square,\varnothing),(\varnothing,\square,\square),\left(\varnothing,\square,\begin{smallmatrix}\square\\\square\end{smallmatrix}\right)$	193	20
321	0 0 3 2<br>0 0 0 0<br>0 0 0 1<br>0 1 0 0	1 1 1 2	$\left(\square,\varnothing,\begin{smallmatrix}\square\\\square\end{smallmatrix}\right)$	202	18
322	0 4 4 3<br>0 0 0 0<br>0 0 0 0<br>0 0 0 0	1 1 1 2	$(\varnothing,\square,\varnothing),(\square,\varnothing,\varnothing),(\square,\square,\square)$	198	9
323	0 0 0 0 2<br>0 0 1 1 0<br>0 0 0 0 0<br>0 0 0 0 0<br>0 3 1 2 0	1 1 1 1 1	$(\varnothing,\varnothing,\square,\blacksquare),(\varnothing,\square,\square,\blacksquare)$	202	22

Continued on next page.

Continued from previous page.

Period ID	Adjacency matrix	Dimension vector	Generalized partitions	Degree	Euler Number
324	0 0 3 3<br>0 0 0 0<br>0 1 0 0<br>0 2 0 0	1 1 1 1	$(\square,\varnothing,\varnothing),(\square,\square,\varnothing)$	178	20
325	0 1 1 3<br>0 0 1 0<br>0 0 0 0<br>0 1 1 0	1 1 1 1	$(\varnothing,\square\square,\varnothing)$	186	20
326	0 2 0 0<br>0 0 1 4<br>0 0 0 0<br>0 0 1 0	1 1 2 2	$(\blacksquare\blacksquare,\begin{smallmatrix}\square\\\square\end{smallmatrix},\varnothing),(\blacksquare\blacksquare,\begin{smallmatrix}\square\\\square\end{smallmatrix},\varnothing),(\blacksquare,\varnothing,\begin{smallmatrix}\square\\\square\end{smallmatrix})$	209	17
327	0 0 1 4<br>0 0 0 0<br>0 3 0 0<br>0 0 1 0	1 1 1 2	$(\varnothing,\square,\square),(\square,\blacksquare,\square)$	204	15
328	0 0 0 0 2<br>0 0 0 1 0<br>0 0 0 1 0<br>0 0 0 0 0<br>0 2 4 0 0	1 1 1 1 1	$(\varnothing,\square,\varnothing,\varnothing),(\varnothing,\square,\square,\blacksquare\blacksquare)$	214	20
329	0 0 0 0 3<br>0 0 0 1 0<br>0 0 0 1 0<br>0 0 0 0 0<br>0 2 3 0 0	1 1 1 1 1	$(\varnothing,\varnothing,\square,\varnothing),(\varnothing,\square,\varnothing,\varnothing)$	209	19
330	0 0 0 4<br>0 0 1 0<br>0 0 0 0<br>0 4 2 0	1 1 1 1	$(\varnothing,\varnothing,\square\square),(\varnothing,\square,\blacksquare),(\square,\varnothing,\varnothing),(\square,\square,\blacksquare\blacksquare)$	204	20
331	0 0 3 4<br>0 0 0 0<br>0 1 0 0<br>0 1 0 0	1 1 1 1	$(\varnothing,\square,\square),(\square,\varnothing,\square)$	188	19
332	0 0 0 0 3<br>0 0 0 0 0<br>0 0 0 1 0<br>0 0 0 0 0<br>0 2 3 1 0	1 1 1 1 1	$(\varnothing,\square,\varnothing,\square),(\square,\varnothing,\square,\blacksquare\blacksquare)$	194	19
333	0 0 0 0 3<br>0 0 0 0 0<br>0 0 0 1 0<br>0 0 0 0 0<br>0 2 3 1 0	1 1 1 1 1	$(\varnothing,\varnothing,\square,\varnothing),(\square,\square,\varnothing,\blacksquare)$	188	22

Continued on next page.

Continued from previous page.

Period ID	Adjacency matrix	Dimension vector	Generalized partitions	Degree	Euler Number
334	0 1 2 4<br>0 0 1 0<br>0 0 0 0<br>0 1 0 0	1 1 1 1	$(\varnothing, \varnothing, \square\square), (\varnothing, \square\square, \varnothing)$	244	24
335	0 2 2 3<br>0 0 1 0<br>0 0 0 0<br>0 1 0 0	1 1 1 1	$(\varnothing, \square\square, \varnothing), (\square, \varnothing, \square)$	208	22
336	0 0 1 5<br>0 0 0 0<br>0 0 0 0<br>0 4 1 0	1 1 1 1	$(\varnothing, \varnothing, \square\square), (\square, \varnothing, \blacksquare), (\square, \varnothing, \varnothing), (\square, \square, \blacksquare)$	198	18
337	0 0 3 4<br>0 0 0 0<br>0 1 0 0<br>0 1 0 0	1 1 1 1	$(\varnothing, \square, \square\square), (\square, \varnothing, \varnothing)$	173	23
338	0 0 0 4 1<br>0 0 0 0 0<br>0 0 0 0 0<br>0 0 0 0 1<br>0 2 2 0 0	1 1 1 1 1	$(\varnothing, \square, \varnothing, \varnothing), (\square, \varnothing, \square\square, \blacksquare)$	199	15
339	0 0 0 3 1<br>0 0 2 0 0<br>0 0 0 0 0<br>0 0 0 0 1<br>0 2 0 0 0	1 1 1 2 2	$(\blacksquare, \square, \varnothing, \varnothing), (\square, \varnothing, \varnothing, \varnothing), (\square, \varnothing, \varnothing, \varnothing), (\square, \varnothing, \varnothing, \varnothing)$	199	13
340	0 0 0 0 2<br>0 0 0 1 0<br>0 0 0 0 0<br>0 0 1 0 0<br>0 2 2 2 0	1 1 1 1 1	$(\varnothing, \varnothing, \square, \varnothing), (\varnothing, \square\square, \varnothing, \blacksquare\blacksquare)$	214	24
341	0 0 0 4<br>0 0 0 0<br>0 0 0 0<br>0 1 1 0	1 1 1 2	$(\varnothing, \square, \varnothing), \left(\square, \varnothing, \begin{smallmatrix}\square\\\square\end{smallmatrix}\right)$	178	19
342	0 0 0 4<br>0 0 0 0<br>0 0 0 0<br>0 1 1 0	1 1 1 2	$\left(\varnothing, \varnothing, \begin{smallmatrix}\square\\\square\end{smallmatrix}\right), (\square, \square, \varnothing)$	188	16
343	0 0 3 4<br>0 0 0 0<br>0 1 0 0<br>0 1 0 0	1 1 1 2	$\left(\varnothing, \varnothing, \begin{smallmatrix}\square\\\square\end{smallmatrix}\right), \left(\varnothing, \square, \begin{smallmatrix}\square\\\square\end{smallmatrix}\right), (\square, \varnothing, \varnothing), (\square, \varnothing, \varnothing)$	183	17

Continued on next page.

Continued from previous page.

Period ID	Adjacency matrix	Dimension vector	Generalized partitions	Degree	Euler Number
344	$\begin{matrix}0&1&0&3\\0&0&4&0\\0&0&0&0\\0&1&0&0\end{matrix}$	1 1 2 2	$\left(\blacksquare\blacksquare,\begin{smallmatrix}\square\\\square\end{smallmatrix},\square\right),(\varnothing,\square,\varnothing)$	178	17
345	$\begin{matrix}0&0&1&4\\0&0&0&0\\0&0&0&0\\0&2&2&0\end{matrix}$	1 1 1 1	$(\varnothing,\square,\square),(\square,\square,\blacksquare)$	177	21
346	$\begin{matrix}0&5&0&3\\0&0&3&0\\0&0&0&0\\0&0&0&0\end{matrix}$	1 1 2 2	$(\varnothing,\square,\square)$	150	18
347	$\begin{matrix}0&1&3&3\\0&0&1&0\\0&0&0&0\\0&1&0&0\end{matrix}$	1 1 1 1	$(\varnothing,\square,\square),(\varnothing,\square\square,\varnothing)$	176	20
348	$\begin{matrix}0&2&2&3\\0&0&0&0\\0&0&0&0\\0&1&1&0\end{matrix}$	1 1 1 1	$(\square,\square,\varnothing),(\square,\square,\varnothing)$	166	22
349	$\begin{matrix}0&1&3&3\\0&0&0&0\\0&0&0&0\\0&1&1&0\end{matrix}$	1 1 1 1	$(\varnothing,\square\square,\varnothing),(\square,\square,\varnothing)$	166	20
350	$\begin{matrix}0&4&2\\0&0&1\\0&0&0\end{matrix}$	1 2 3	$\left(\varnothing,\begin{smallmatrix}\square\\\square\\\square\end{smallmatrix}\right),\left(\varnothing,\begin{smallmatrix}\square\\\square\\\square\end{smallmatrix}\right),\left(\varnothing,\begin{smallmatrix}\square\\\square\\\square\end{smallmatrix}\right)$	177	18
351	$\begin{matrix}0&0&0&4&1\\0&0&0&0&0\\0&0&0&0&0\\0&0&0&0&1\\0&2&2&0&0\end{matrix}$	1 1 1 1 1	$(\varnothing,\square,\square,\blacksquare),(\square,\varnothing,\square,\varnothing)$	187	24
352	$\begin{matrix}0&0&0&3\\0&0&1&0\\0&0&0&0\\0&4&3&0\end{matrix}$	1 1 1 1	$(\varnothing,\square,\blacksquare),(\varnothing,\square,\varnothing),(\square,\varnothing,\varnothing),(\square,\square,\blacksquare\blacksquare)$	172	20
353	$\begin{matrix}0&0&0&3\\0&0&1&0\\0&0&0&0\\0&4&3&0\end{matrix}$	1 1 1 1	$(\varnothing,\square,\blacksquare),(\varnothing,\square\square,\blacksquare\blacksquare),(\square,\varnothing,\varnothing),(\square,\varnothing,\varnothing)$	178	22
354	$\begin{matrix}0&0&5\\0&0&0\\0&3&0\end{matrix}$	1 1 1	$(\varnothing,\square\square),(\square\square,\blacksquare)$	176	16

Continued on next page.

Continued from previous page.

Period ID	Adjacency matrix	Dimension vector	Generalized partitions	Degree	Euler Number
355	0 0 4<br>0 0 0<br>0 4 0	1 1 1	(□, □), (□□, ■■)	160	32
356	0 1 2 3<br>0 0 1 0<br>0 0 0 0<br>0 1 0 0	1 1 1 1	(∅, □□, □)	149	34
357	0 0 1 4<br>0 0 0 0<br>0 0 0 0<br>0 3 1 0	1 1 1 1	(∅, □, □), (□□, ∅, ■)	162	19
358	0 0 1 4<br>0 0 0 0<br>0 0 0 0<br>0 3 1 0	1 1 1 1	(□, ∅, □), (□, □, ■)	151	25
359	0 0 1 4<br>0 0 0 0<br>0 0 0 0<br>0 2 2 0	1 1 1 1	(∅, □□, ∅), (□, ∅, ∅)	160	24
360	0 0 1 4<br>0 0 0 0<br>0 0 0 0<br>0 3 1 0	1 1 1 1	(□, ∅, ∅), (□, □, ∅)	145	33
361	0 4 3<br>0 0 1<br>0 0 0	1 1 2	$(\varnothing, \ydiagram{1,1}), (\varnothing, \ydiagram{1,1}), (\square, \ydiagram{1,1})$	129	31
362	0 0 4<br>0 0 0<br>0 4 0	1 1 1	(□, ■), (□□, ∅)	112	52
363	0 0 4<br>0 0 0<br>0 4 0	1 1 1	(∅, □□), (□□, ■■)	384	16
364	0 3 0<br>0 0 5<br>0 0 0	1 1 2	$(■■, \ydiagram{1,1}), (■■, \ydiagram{1,1}), (■■, \ydiagram{1,1})$, (□□, ∅)	320	8
365	0 6<br>0 0	1 2	$(\ydiagram{1,1}), (\ydiagram{1,1}), (\ydiagram{1,1}), (\ydiagram{1,1})$	224	12
366	0 0 0 4<br>0 0 1 0<br>0 0 0 0<br>0 3 1 0	1 1 1 1	(∅, ∅, □□), (□, □, ■■)	336	20

Continued on next page.

Continued from previous page.

Period ID	Adjacency matrix	Dimension vector	Generalized partitions	Degree	Euler Number
367	0 0 0 1 3<br>0 0 0 0 0<br>0 0 0 1 0<br>0 0 0 0 0<br>0 2 3 0 0	1 1 1 1 1	(∅, ∅, □, □),(∅, □, ∅, ■)	304	16
368	0 0 1 4<br>0 0 1 0<br>0 0 0 0<br>0 3 0 0	1 1 1 1	(∅, ∅, □□),(□, □, ■)	282	20
369	0 0 0 4 1<br>0 0 0 0 0<br>0 0 0 0 0<br>0 0 0 0 1<br>0 2 2 0 0	1 1 1 1 1	(∅, ∅, □□, ∅),(∅, □, ∅, ∅)	304	20
370	0 0 0 1 3<br>0 0 1 0 0<br>0 0 0 0 0<br>0 0 0 0 0<br>0 3 1 1 0	1 1 1 1 1	(∅, ∅, □, □),(□, □, ∅, ■■)	294	25
371	0 0 1 2 2 0<br>0 0 0 0 0 0<br>0 0 0 0 0 0<br>0 0 0 0 0 0<br>0 0 1 0 0 4<br>0 2 0 0 0 0	1 1 1 1 1 1	(∅, ∅, ∅, ■, □),(∅, ∅, □, ■, □),(∅, □, ∅, ■, □)	288	20
372	0 3 1 4<br>0 0 0 0<br>0 0 0 0<br>0 0 1 0	1 1 2 2	(∅, ∅, $\substack{\square\\\square}$),(∅, $\substack{\square\\\square}$, ∅),(∅, $\substack{\square\\\square}$, ∅),(□, ∅, ∅)	272	12
373	0 0 0 3<br>0 0 1 0<br>0 0 0 0<br>0 3 2 0	1 1 1 1	(∅, □□, ■■),(□, ∅, ∅)	256	16
374	0 7 2<br>0 0 1<br>0 0 0	1 1 2	(□, □),(□, □)	193	21
375	0 0 2 3<br>0 0 1 0<br>0 0 0 0<br>0 3 0 0	1 1 1 1	(∅, □, □),(□, □, ■)	208	20
376	0 0 1 3<br>0 0 1 0<br>0 0 0 0<br>0 4 0 0	1 1 1 1	(□, ∅, ■),(□, □, ∅)	186	33

Continued on next page.

Continued from previous page.

Period ID	Adjacency matrix	Dimension vector	Generalized partitions	Degree	Euler Number
377	0 0 0 0 3<br>0 0 0 1 0<br>0 0 0 0 0<br>0 0 0 0 0<br>0 2 3 1 0	1 1 1 1 1	$(\varnothing,\square,\varnothing,\varnothing),(\varnothing,\square,\varnothing,\varnothing)$	288	24
378	0 0 0 1 2<br>0 0 0 0 0<br>0 0 0 1 0<br>0 0 0 0 0<br>0 2 4 0 0	1 1 1 1 1	$(\varnothing,\square\square,\varnothing,\blacksquare\blacksquare),(\square,\varnothing,\square,\blacksquare)$	282	22
379	0 0 0 2 2 0<br>0 0 0 0 0 0<br>0 0 0 0 0 2<br>0 0 1 0 0 0<br>0 0 1 0 0 0<br>0 2 0 0 0 0	1 1 1 1 1 1	$(\varnothing,\varnothing,\varnothing,\varnothing,\square)$	272	20
380	0 0 0 1 4<br>0 0 1 0 0<br>0 0 0 0 0<br>0 0 0 0 0<br>0 2 1 1 0	1 1 1 1 1	$(\varnothing,\varnothing,\varnothing,\square\square),(\varnothing,\square,\square,\blacksquare)$	256	22
381	0 0 0 3 2<br>0 0 0 0 0<br>0 0 0 0 0<br>0 0 0 0 1<br>0 2 2 0 0	1 1 1 1 1	$(\varnothing,\varnothing,\square,\square),(\varnothing,\square,\varnothing,\varnothing)$	256	16
382	0 0 0 2 0 1<br>0 0 0 0 1 0<br>0 0 0 0 0 0<br>0 0 0 0 0 1<br>0 0 4 0 0 0<br>0 2 0 0 1 0	1 1 1 1 1 1	$(\varnothing,\square,\varnothing,\blacksquare,\varnothing),(\varnothing,\square,\varnothing,\blacksquare,\square),(\varnothing,\square,\varnothing,\varnothing,\blacksquare)$	246	21
383	0 0 0 4<br>0 0 0 0<br>0 0 0 0<br>0 3 4 0	1 1 1 1	$(\varnothing,\varnothing,\square\square),(\varnothing,\square,\blacksquare),(\varnothing,\square,\varnothing),(\square,\square,\blacksquare\blacksquare)$	240	16
384	0 0 0 4<br>0 0 0 0<br>0 0 0 0<br>0 3 4 0	1 1 1 1	$(\varnothing,\square,\blacksquare),(\varnothing,\square,\varnothing),(\square,\varnothing,\varnothing),(\square,\varnothing,\varnothing)$	224	13
385	0 1 5<br>0 0 0<br>0 1 0	1 2 2	$\left(\varnothing,\begin{smallmatrix}\square\\\square\end{smallmatrix}\right),\left(\varnothing,\begin{smallmatrix}\square\\\square\end{smallmatrix}\right),\left(\begin{smallmatrix}\square\\\square\end{smallmatrix},\varnothing\right),\left(\begin{smallmatrix}\square\\\square\end{smallmatrix},\varnothing\right)$	193	15

Continued on next page.

Continued from previous page.

Period ID	Adjacency matrix	Dimension vector	Generalized partitions	Degree	Euler Number
386	0 0 4 4<br>0 0 0 0<br>0 1 0 0<br>0 1 0 0	1 1 1 1	$(\varnothing,\varnothing,\square\square),(\varnothing,\square\square,\varnothing),(\square,\varnothing,\varnothing)$	252	20
387	0 0 0 3 2<br>0 0 0 0 0<br>0 0 0 0 0<br>0 0 0 0 1<br>0 2 2 0 0	1 1 1 1 1	$(\varnothing,\varnothing,\varnothing,\square\square),(\varnothing,\square,\square,\blacksquare)$	256	20
388	0 0 0 5<br>0 0 2 0<br>0 0 0 0<br>0 1 0 0	1 1 1 2	$\left(\varnothing,\varnothing,\begin{smallmatrix}\square\\\square\end{smallmatrix}\right),\left(\varnothing,\varnothing,\begin{smallmatrix}\square\\\square\end{smallmatrix}\right),\left(\varnothing,\varnothing,\begin{smallmatrix}\square\\\square\end{smallmatrix}\right),(\square,\varnothing,\varnothing)$	240	12
389	0 2 5<br>0 0 1<br>0 0 0	1 1 1	$(\varnothing,\square\square),(\varnothing,\square\square)$	192	20
390	0 0 0 0 3<br>0 0 0 0 0<br>0 0 0 0 0<br>0 0 0 0 0<br>0 2 3 4 0	1 1 1 1 1	$(\varnothing,\varnothing,\square,\blacksquare),(\varnothing,\varnothing,\square,\varnothing),(\varnothing,\square,\varnothing,\varnothing),(\varnothing,\square,\square,\blacksquare\blacksquare)$	240	16
391	0 0 1 2 2 0<br>0 0 0 0 0 0<br>0 0 0 0 0 0<br>0 0 0 0 0 0<br>0 0 1 0 0 4<br>0 2 0 0 0 0	1 1 1 1 1 1	$(\varnothing,\varnothing,\square,\blacksquare,\square),(\varnothing,\square,\varnothing,\blacksquare,\square),(\square,\varnothing,\varnothing,\blacksquare,\varnothing)$	230	22
392	0 0 0 2 2<br>0 0 0 0 0<br>0 0 0 1 0<br>0 0 0 0 0<br>0 2 3 0 0	1 1 1 1 1	$(\varnothing,\square,\square,\blacksquare),(\square,\varnothing,\square,\blacksquare)$	219	20
393	0 0 0 2 3<br>0 0 1 0 0<br>0 0 0 0 0<br>0 0 0 0 0<br>0 2 1 1 0	1 1 1 1 1	$(\varnothing,\varnothing,\square\square,\varnothing),(\varnothing,\square,\varnothing,\varnothing)$	220	22
394	0 0 0 2 3<br>0 0 1 0 0<br>0 0 0 0 0<br>0 0 0 0 0<br>0 2 1 1 0	1 1 1 1 1	$(\varnothing,\varnothing,\square,\square),(\varnothing,\square,\square,\blacksquare)$	214	19

Continued on next page.

Continued from previous page.

Period ID	Adjacency matrix	Dimension vector	Generalized partitions	Degree	Euler Number
395	0 0 0 3<br>0 0 1 0<br>0 0 0 0<br>0 2 3 0	1 1 1 1	$(\varnothing,\square,\varnothing),(\varnothing,\square\square,\blacksquare\blacksquare)$	208	14
396	0 0 0 0 3<br>0 0 0 0 0<br>0 0 0 0 0<br>0 0 0 0 0<br>0 3 3 3 0	1 1 1 1 1	$(\varnothing,\square,\varnothing,\varnothing),(\varnothing,\square,\square,\blacksquare\blacksquare),(\square,\varnothing,\varnothing,\varnothing),(\square,\varnothing,\square,\blacksquare\blacksquare)$	204	18
397	0 0 0 3<br>0 0 0 0<br>0 0 0 0<br>0 3 3 0	1 1 1 1	$(\square,\varnothing,\varnothing),(\square,\square,\blacksquare)$	192	18
398	0 3 4<br>0 0 1<br>0 0 0	1 1 2	$\left(\varnothing,\begin{smallmatrix}\square\\\square\end{smallmatrix}\right),(\varnothing,\square\square)$	164	20
399	0 0 0 0 2<br>0 0 0 1 0<br>0 0 0 1 0<br>0 0 0 0 0<br>0 2 4 0 0	1 1 1 1 1	$(\varnothing,\varnothing,\square,\varnothing),(\varnothing,\square\square,\varnothing,\blacksquare\blacksquare)$	230	22
400	0 0 0 0 3<br>0 0 0 0 0<br>0 0 0 1 0<br>0 0 0 0 0<br>0 2 3 1 0	1 1 1 1 1	$(\varnothing,\varnothing,\varnothing,\square\square),(\square,\square,\square,\blacksquare\blacksquare\blacksquare)$	224	12
401	0 0 0 0 4<br>0 0 0 1 0<br>0 0 0 0 0<br>0 0 1 0 0<br>0 2 1 1 0	1 1 1 1 1	$(\varnothing,\varnothing,\varnothing,\square\square),(\varnothing,\square,\varnothing,\varnothing)$	224	22
402	0 0 3 4<br>0 0 0 0<br>0 1 0 0<br>0 1 0 0	1 1 1 1	$(\varnothing,\varnothing,\square\square),(\square,\square,\varnothing)$	204	20
403	0 0 0 0 4<br>0 0 0 3 0<br>0 0 0 0 0<br>0 0 2 0 0<br>0 1 0 0 0	1 1 1 1 2	$(\blacksquare,\varnothing,\square,\varnothing),\left(\varnothing,\varnothing,\varnothing,\begin{smallmatrix}\square\\\square\end{smallmatrix}\right),(\varnothing,\varnothing,\square,\varnothing),\left(\varnothing,\square,\blacksquare,\begin{smallmatrix}\square\\\square\end{smallmatrix}\right)$	204	18
404	0 2 0 0<br>0 0 1 4<br>0 0 0 0<br>0 0 1 0	1 1 2 2	$\left(\blacksquare\blacksquare,\varnothing,\begin{smallmatrix}\square\\\square\end{smallmatrix}\right),\left(\blacksquare\blacksquare,\begin{smallmatrix}\square\\\square\end{smallmatrix},\varnothing\right),\left(\blacksquare,\begin{smallmatrix}\square\\\square\end{smallmatrix},\varnothing\right)$	193	17

Continued on next page.

Continued from previous page.

Period ID	Adjacency matrix	Dimension vector	Generalized partitions	Degree	Euler Number
405	$\begin{smallmatrix}0&0&0&4\\0&0&0&0\\0&0&0&0\\0&2&3&0\end{smallmatrix}$	1 1 1 1	$(\varnothing,\varnothing,\square\!\square),(\square,\square,\blacksquare)$	192	22
406	$\begin{smallmatrix}0&3&0&0\\0&0&2&3\\0&0&0&0\\0&0&1&0\end{smallmatrix}$	1 1 2 2	$\left(\blacksquare\!\blacksquare,\begin{smallmatrix}\square\\\square\end{smallmatrix},\varnothing\right),\left(\blacksquare\!\blacksquare,\begin{smallmatrix}\square\\\square\end{smallmatrix},\varnothing\right),(\varnothing,\square,\varnothing)$	183	16
407	$\begin{smallmatrix}0&6&2\\0&0&1\\0&0&0\end{smallmatrix}$	1 1 2	$(\square,\square),\left(\square,\begin{smallmatrix}\square\\\square\end{smallmatrix}\right)$	161	31
408	$\begin{smallmatrix}0&0&1&2&2&0\\0&0&0&0&0&0\\0&0&0&0&0&0\\0&0&1&0&0&0\\0&0&1&0&0&3\\0&2&0&0&0&0\end{smallmatrix}$	1 1 1 1 1 1	$(\varnothing,\varnothing,\varnothing,\blacksquare,\square),(\varnothing,\square,\varnothing,\blacksquare,\square),(\square,\square,\varnothing,\varnothing,\blacksquare)$	209	23
409	$\begin{smallmatrix}0&1&2&4\\0&0&0&0\\0&0&0&1\\0&1&0&0\end{smallmatrix}$	1 1 1 1	$(\varnothing,\varnothing,\square\!\square),(\square,\varnothing,\square)$	182	22
410	$\begin{smallmatrix}0&0&0&1&3\\0&0&0&0&0\\0&0&0&1&0\\0&0&0&0&0\\0&2&3&0&0\end{smallmatrix}$	1 1 1 1 1	$(\varnothing,\square,\varnothing,\blacksquare),(\square,\varnothing,\square,\varnothing)$	197	24
411	$\begin{smallmatrix}0&0&0&0&3\\0&0&0&1&0\\0&0&0&0&0\\0&0&1&0&0\\0&2&2&1&0\end{smallmatrix}$	1 1 1 1 1	$(\varnothing,\square,\varnothing,\varnothing),(\varnothing,\square,\varnothing,\varnothing)$	203	19
412	$\begin{smallmatrix}0&0&0&0&2\\0&0&0&1&0\\0&0&0&1&0\\0&0&0&0&0\\0&2&3&1&0\end{smallmatrix}$	1 1 1 1 1	$(\varnothing,\varnothing,\square,\varnothing),(\varnothing,\square,\square,\blacksquare\!\blacksquare)$	188	21
413	$\begin{smallmatrix}0&0&3&0\\0&0&0&0\\0&1&0&4\\0&1&0&0\end{smallmatrix}$	1 1 1 2	$\left(\varnothing,\blacksquare\!\blacksquare,\begin{smallmatrix}\square\\\square\end{smallmatrix}\right),(\varnothing,\varnothing,\square),(\square\!\square,\blacksquare\!\blacksquare,\varnothing)$	188	14
414	$\begin{smallmatrix}0&0&0&0&4\\0&0&0&0&0\\0&0&0&0&0\\0&0&0&0&0\\0&2&3&3&0\end{smallmatrix}$	1 1 1 1 1	$(\varnothing,\varnothing,\square,\varnothing),(\varnothing,\square,\varnothing,\varnothing),(\varnothing,\square,\square,\blacksquare\!\blacksquare),(\square,\varnothing,\varnothing,\varnothing)$	188	20

Continued on next page.

Continued from previous page.

Period ID	Adjacency matrix	Dimension vector	Generalized partitions	Degree	Euler Number
415	0 0 0 4<br>0 0 1 0<br>0 0 0 0<br>0 3 3 0	1 1 1 1	$(\varnothing,\varnothing,\square\square),(\varnothing,\square,\blacksquare),(\varnothing,\square\square,\blacksquare\blacksquare),(\square,\varnothing,\varnothing)$	184	24
416	0 0 0 0 3<br>0 0 0 0 0<br>0 0 0 1 0<br>0 0 0 0 0<br>0 2 3 1 0	1 1 1 1 1	$(\varnothing,\square,\square,\blacksquare),(\square,\varnothing,\varnothing,\varnothing)$	182	24
417	0 0 0 0 3<br>0 0 0 0 0<br>0 0 0 1 0<br>0 0 0 0 0<br>0 2 3 1 0	1 1 1 1 1	$(\varnothing,\square,\varnothing,\varnothing),(\square,\varnothing,\square,\blacksquare)$	182	22
418	0 0 0 4<br>0 0 0 0<br>0 0 0 0<br>0 2 3 0	1 1 1 1	$(\varnothing,\square,\square),(\square,\varnothing,\varnothing)$	176	20
419	0 1 3 3<br>0 0 0 0<br>0 1 0 0<br>0 1 0 0	1 1 1 1	$(\square,\varnothing,\square),(\square,\square,\varnothing)$	162	21
420	0 0 0 3<br>0 0 2 0<br>0 0 0 0<br>0 3 1 0	1 1 1 1	$(\varnothing,\square,\varnothing),(\varnothing,\square,\varnothing)$	161	22
421	0 0 0 5<br>0 0 2 0<br>0 0 0 0<br>0 1 0 0	1 1 1 2	$\left(\varnothing,\varnothing,\begin{smallmatrix}\square\\\square\end{smallmatrix}\right),\left(\varnothing,\varnothing,\begin{smallmatrix}\square\\\square\end{smallmatrix}\right),\left(\varnothing,\varnothing,\begin{smallmatrix}\square\\\square\end{smallmatrix}\right),(\varnothing,\square,\varnothing)$	183	15
422	0 0 4 4<br>0 0 0 0<br>0 0 0 0<br>0 1 0 0	1 1 1 2	$(\varnothing,\square,\varnothing),(\varnothing,\square,\square),\left(\square,\varnothing,\begin{smallmatrix}\square\\\square\end{smallmatrix}\right)$	172	18
423	0 0 0 5<br>0 0 2 0<br>0 0 0 0<br>0 1 0 0	1 1 1 2	$\left(\blacksquare,\square,\begin{smallmatrix}\square\\\square\end{smallmatrix}\right),\left(\varnothing,\varnothing,\begin{smallmatrix}\square\\\square\end{smallmatrix}\right),\left(\varnothing,\varnothing,\begin{smallmatrix}\square\\\square\end{smallmatrix}\right),(\square,\varnothing,\varnothing)$	167	19
424	0 0 3 3<br>0 0 0 0<br>0 3 0 1<br>0 0 0 0	1 1 1 2	$\left(\varnothing,\varnothing,\begin{smallmatrix}\square\\\square\end{smallmatrix}\right),\left(\varnothing,\varnothing,\begin{smallmatrix}\square\\\square\end{smallmatrix}\right),(\square,\blacksquare,\varnothing),\left(\square,\blacksquare,\begin{smallmatrix}\square\\\square\end{smallmatrix}\right)$	161	20

Continued on next page.

Continued from previous page.

Period ID	Adjacency matrix	Dimension vector	Generalized partitions	Degree	Euler Number
425	$\begin{smallmatrix}0&0&6\\0&0&0\\0&2&0\end{smallmatrix}$	1 1 1	$(\varnothing,\square\square),(\square,\square)$	160	30
426	$\begin{smallmatrix}0&0&5\\0&0&0\\0&1&0\end{smallmatrix}$	1 2 3	$(\square,\varnothing),\left(\square,\substack{\square\\\square}\right)$	147	19
427	$\begin{smallmatrix}0&0&0&3\\0&0&2&0\\0&0&0&0\\0&3&1&0\end{smallmatrix}$	1 1 1 1	$(\varnothing,\varnothing,\square\square),(\varnothing,\square\square,\blacksquare\blacksquare)$	192	4
428	$\begin{smallmatrix}0&0&0&4\\0&0&2&0\\0&0&0&0\\0&2&0&0\end{smallmatrix}$	1 1 1 2	$\left(\blacksquare,\square,\substack{\square\\\square}\right),(\square,\varnothing,\varnothing),(\square,\varnothing,\varnothing),(\square,\varnothing,\varnothing)$	163	17
429	$\begin{smallmatrix}0&0&0&3&2\\0&0&0&0&0\\0&0&0&0&0\\0&0&0&0&1\\0&2&2&0&0\end{smallmatrix}$	1 1 1 1 1	$(\varnothing,\square,\square,\varnothing),(\square,\varnothing,\varnothing,\varnothing)$	167	24
430	$\begin{smallmatrix}0&0&0&4\\0&0&4&0\\0&0&0&0\\0&1&0&0\end{smallmatrix}$	1 1 1 2	$(\blacksquare,\square,\varnothing),\left(\blacksquare,\square,\substack{\square\\\square}\right),\left(\varnothing,\varnothing,\substack{\square\\\square}\right),(\varnothing,\square,\varnothing)$	162	18
431	$\begin{smallmatrix}0&3&3\\0&0&1\\0&0&0\end{smallmatrix}$	1 2 2	$\left(\varnothing,\substack{\square\\\square}\right),(\varnothing,\square\square)$	148	20
432	$\begin{smallmatrix}0&0&0&5\\0&0&1&0\\0&0&0&0\\0&3&2&0\end{smallmatrix}$	1 1 1 1	$(\varnothing,\varnothing,\square\square),(\varnothing,\square,\blacksquare),(\varnothing,\square,\varnothing),(\square,\varnothing,\varnothing)$	156	20
433	$\begin{smallmatrix}0&0&3&3\\0&0&0&0\\0&1&0&0\\0&1&0&0\end{smallmatrix}$	1 1 1 1	$(\square,\square,\square)$	141	33
434	$\begin{smallmatrix}0&2&2&3\\0&0&0&1\\0&0&0&0\\0&0&1&0\end{smallmatrix}$	1 1 1 1	$(\varnothing,\square,\square),(\varnothing,\square,\square)$	161	24
435	$\begin{smallmatrix}0&0&0&4\\0&0&1&0\\0&0&0&0\\0&3&3&0\end{smallmatrix}$	1 1 1 1	$(\varnothing,\square,\blacksquare),(\varnothing,\square,\varnothing),(\varnothing,\square,\varnothing),(\square,\varnothing,\varnothing)$	151	22

Continued on next page.

Continued from previous page.

Period ID	Adjacency matrix	Dimension vector	Generalized partitions	Degree	Euler Number
436	0 0 1 5<br>0 0 0 0<br>0 0 0 0<br>0 2 1 0	1 1 1 1	(∅, □, □), (□, ∅, □)	151	25
437	0 0 0 3<br>0 0 1 0<br>0 0 0 0<br>0 3 2 0	1 1 1 1	(∅, □□, ■■), (□, ∅, □)	148	22
438	0 0 5 0<br>0 0 0 0<br>0 3 0 3<br>0 0 0 0	1 1 1 2	(∅, ∅, □), (□, ■, □)	131	25
439	0 5 6<br>0 0 0<br>0 0 0	1 1 4	(∅, □<br>□<br>□<br>□), (∅, □<br>□<br>□<br>□), (∅, □<br>□<br>□<br>□), (∅, □<br>□<br>□<br>□), (□, □)	141	19
440	0 0 0 4<br>0 0 1 0<br>0 0 0 0<br>0 3 1 0	1 1 1 1	(∅, □, ∅), (□, ∅, □)	130	26
441	0 1 5<br>0 0 0<br>0 2 0	1 1 1	(□, □), (□, □)	129	41
442	0 0 6<br>0 0 0<br>0 2 0	1 1 1	(∅, □□), (∅, □□)	256	0
443	0 5<br>0 0	1 2	(□□<br>□)	192	16
444	0 0 0 4<br>0 0 1 0<br>0 0 0 0<br>0 2 2 0	1 1 1 1	(∅, ∅, □□), (∅, □□, ■■)	288	24
445	0 0 1 5<br>0 0 0 0<br>0 0 0 0<br>0 2 1 0	1 1 1 1	(∅, ∅, □□), (∅, □, □)	240	8
446	0 0 1 5<br>0 0 1 0<br>0 0 0 0<br>0 2 0 0	1 1 1 1	(∅, ∅, □□), (∅, □, □)	218	20

Continued on next page.

Continued from previous page.

Period ID	Adjacency matrix	Dimension vector	Generalized partitions	Degree	Euler Number
447	0 0 0 1 3<br>0 0 1 0 0<br>0 0 0 0 0<br>0 0 0 0 0<br>0 2 2 1 0	1 1 1 1 1	(∅, ∅, □, □),(∅, □□, ∅, ■■)	252	30
448	0 0 0 0 2<br>0 0 0 1 0<br>0 0 0 0 0<br>0 0 0 0 0<br>0 2 4 1 0	1 1 1 1 1	(∅, □, □, ■■),(∅, □□, ∅, ■■)	240	20
449	0 0 1 6<br>0 0 0 0<br>0 0 0 0<br>0 3 1 0	1 1 1 1	(∅, ∅, □□),(∅, ∅, □□),(□, ∅, ■),(□, □, ■)	188	8
450	0 0 0 4<br>0 0 2 0<br>0 0 0 0<br>0 2 0 0	1 1 1 2	(∅, ∅, $\begin{smallmatrix}\square\\\square\end{smallmatrix}$),(□, ∅, ∅),(□, ∅, ∅),(□, ∅, ∅)	224	12
451	0 0 2 3<br>0 0 1 0<br>0 0 0 0<br>0 3 0 0	1 1 1 1	(∅, □□, ∅),(□, ∅, ∅)	180	36
452	0 0 0 0 2<br>0 0 0 1 0<br>0 0 0 0 0<br>0 0 0 0 0<br>0 2 3 2 0	1 1 1 1 1	(∅, □, □, ■■),(∅, □, □, ■■)	224	16
453	0 0 2 4<br>0 0 0 0<br>0 0 0 0<br>0 2 1 0	1 1 1 1	(∅, □, □),(∅, □, □)	208	4
454	0 0 0 0 4<br>0 0 0 0 0<br>0 0 0 0 0<br>0 0 0 0 0<br>0 2 3 3 0	1 1 1 1 1	(∅, ∅, ∅, □□),(∅, ∅, □, ∅),(∅, □, □, ■■),(□, □, ∅, ■■)	204	22
455	0 0 2 4<br>0 0 1 0<br>0 0 0 0<br>0 2 0 0	1 1 1 1	(∅, □, □),(∅, □, □)	176	24
456	0 0 0 3<br>0 0 0 0<br>0 0 0 0<br>0 2 4 0	1 1 1 1	(∅, □, ■),(□, ∅, □)	270	21

Continued on next page.

Continued from previous page.

Period ID	Adjacency matrix	Dimension vector	Generalized partitions	Degree	Euler Number
457	0 0 0 4<br>0 0 1 0<br>0 0 0 0<br>0 4 2 0	1 1 1 1	$(\varnothing,\varnothing,\square\square),(\varnothing,\square,\blacksquare),(\varnothing,\square,\varnothing),(\square\square,\varnothing,\blacksquare\blacksquare)$	208	24
458	0 0 0 3<br>0 0 1 0<br>0 0 0 0<br>0 3 2 0	1 1 1 1	$(\varnothing,\square,\blacksquare),(\varnothing,\square,\square)$	206	13
459	0 0 0 2 2<br>0 0 0 0 0<br>0 0 0 1 0<br>0 0 0 0 0<br>0 2 3 0 0	1 1 1 1 1	$(\varnothing,\varnothing,\square\square,\varnothing),(\square,\square,\varnothing,\blacksquare\blacksquare)$	202	28
460	0 3 0 0<br>0 0 2 3<br>0 0 0 0<br>0 0 1 0	1 1 2 2	$\left(\blacksquare\blacksquare,\substack{\square\\\square},\varnothing\right),\left(\blacksquare\blacksquare,\substack{\square\\\square},\varnothing\right),\left(\blacksquare\blacksquare,\substack{\square\\\square},\varnothing\right),(\square\square,\varnothing,\varnothing)$	208	12
461	0 0 1 2 2 0<br>0 0 0 0 0 0<br>0 0 0 0 0 0<br>0 0 0 0 0 0<br>0 0 1 0 0 4<br>0 2 0 0 0 0	1 1 1 1 1 1	$(\varnothing,\varnothing,\varnothing,\blacksquare,\square),(\varnothing,\varnothing,\square,\blacksquare,\square),(\square,\square,\varnothing,\blacksquare,\varnothing)$	198	24
462	0 0 0 0 4<br>0 0 0 0 0<br>0 0 0 0 0<br>0 0 0 0 0<br>0 2 3 3 0	1 1 1 1 1	$(\varnothing,\varnothing,\square,\varnothing),(\varnothing,\varnothing,\square,\varnothing),(\varnothing,\square,\varnothing,\varnothing),(\square,\square,\varnothing,\blacksquare\blacksquare)$	188	19
463	0 0 2 5<br>0 0 0 0<br>0 1 0 0<br>0 1 0 0	1 1 1 1	$(\varnothing,\varnothing,\square\square),(\square,\varnothing,\square)$	172	20
464	0 0 5<br>0 0 0<br>0 1 0	1 2 3	$\left(\varnothing,\substack{\square\\\square\\\square}\right),\left(\varnothing,\substack{\square\\\square\\\square}\right),\left(\substack{\square\\\square},\varnothing\right),\left(\substack{\square\\\square},\varnothing\right)$	162	14
465	0 0 0 2 2<br>0 0 0 0 0<br>0 0 0 0 0<br>0 0 1 0 0<br>0 4 1 0 0	1 1 1 1 1	$(\square,\square,\varnothing,\blacksquare),(\square\square,\varnothing,\varnothing,\blacksquare\blacksquare)$	188	24
466	0 0 0 2<br>0 0 1 0<br>0 0 0 0<br>0 3 3 0	1 1 1 1	$(\varnothing,\square,\blacksquare),(\varnothing,\square\square,\blacksquare)$	185	17

Continued on next page.

Continued from previous page.

Period ID	Adjacency matrix	Dimension vector	Generalized partitions	Degree	Euler Number
467	0 0 0 4<br>0 0 1 0<br>0 0 0 0<br>0 3 3 0	1 1 1 1	(∅, ∅, □□),(∅, □, ■),(∅, □, ∅),(□, □, ■■)	172	22
468	0 0 0 3<br>0 0 1 0<br>0 0 0 0<br>0 4 3 0	1 1 1 1	(∅, □, ■),(∅, □, ∅),(∅, □, ∅),(□□, ∅, ■■)	182	22
469	0 2 2 3<br>0 0 0 1<br>0 0 0 0<br>0 0 1 0	1 1 1 1	(∅, ∅, □□),(∅, □□, ∅)	172	24
470	0 0 0 0 2<br>0 0 0 1 0<br>0 0 0 1 0<br>0 0 0 0 0<br>0 2 3 1 0	1 1 1 1 1	(∅, ∅, □□, ■■),(∅, □, ∅, ∅)	182	20
471	0 0 0 2 2<br>0 0 1 0 0<br>0 0 0 0 0<br>0 0 0 0 1<br>0 2 2 0 0	1 1 1 1 1	(∅, □, ∅, ∅),(∅, □, ∅, ∅)	172	22
472	0 0 0 4<br>0 0 2 0<br>0 0 0 0<br>0 2 0 0	1 1 1 2	(∅, ∅, $\begin{smallmatrix}\square\\ \square\end{smallmatrix}$),(∅, □, ∅),(□, ∅, ∅),(□, ∅, ∅)	162	18
473	0 1 4<br>0 0 0<br>0 1 0	1 2 2	($\begin{smallmatrix}\square\\ \square\end{smallmatrix}$, □)	146	20
474	0 3 5<br>0 0 0<br>0 0 0	1 2 2	(∅, $\begin{smallmatrix}\square\\ \square\end{smallmatrix}$),(∅, $\begin{smallmatrix}\square\\ \square\end{smallmatrix}$),(□, $\begin{smallmatrix}\square\\ \square\end{smallmatrix}$)	165	6
475	0 0 0 0 3<br>0 0 0 0 0<br>0 0 0 0 0<br>0 0 0 0 0<br>0 2 3 4 0	1 1 1 1 1	(∅, ∅, □, ■),(∅, □, ∅, ∅),(∅, □, □, ■■),(□, ∅, □, ■)	162	22
476	0 1 3 3<br>0 0 0 0<br>0 1 0 0<br>0 1 0 0	1 1 1 1	(∅, □, □),(□□, ∅, ∅)	156	20

Continued on next page.

Continued from previous page.

Period ID	Adjacency matrix	Dimension vector	Generalized partitions	Degree	Euler Number
477	0 0 1 2 2<br>0 0 0 0 0<br>0 0 0 0 0<br>0 0 1 0 0<br>0 3 1 0 0	1 1 1 1 1	$(\square,\square,\varnothing,\blacksquare),(\square,\square,\varnothing,\blacksquare)$	167	22
478	0 2 0 0<br>0 0 1 4<br>0 0 0 0<br>0 0 1 0	1 1 2 3	$\left(\blacksquare\blacksquare,\varnothing,\begin{smallmatrix}\square\\\square\\\square\end{smallmatrix}\right),\left(\blacksquare\blacksquare,\begin{smallmatrix}\square\\\square\end{smallmatrix},\varnothing\right),\left(\blacksquare\blacksquare,\begin{smallmatrix}\square\\\square\end{smallmatrix},\varnothing\right),\left(\blacksquare\blacksquare,\begin{smallmatrix}\square\\\square\end{smallmatrix},\varnothing\right)$	162	16
479	0 1 2 4<br>0 0 0 0<br>0 1 0 0<br>0 1 0 0	1 1 1 1	$(\square,\varnothing,\square),(\square,\varnothing,\square)$	146	24
480	0 0 0 3<br>0 0 1 0<br>0 0 0 0<br>0 3 4 0	1 1 1 1	$(\varnothing,\square,\blacksquare),(\varnothing,\square,\varnothing),(\varnothing,\square,\varnothing),(\square,\square,\blacksquare\blacksquare)$	146	22
481	0 0 1 5<br>0 0 0 0<br>0 0 0 0<br>0 2 1 0	1 1 1 1	$(\varnothing,\varnothing,\square\square),(\square,\square,\varnothing)$	150	32
482	0 0 0 2 3<br>0 0 0 0 0<br>0 0 0 0 0<br>0 3 3 0 0<br>0 0 0 0 0	1 1 1 1 2	$\left(\varnothing,\varnothing,\square,\begin{smallmatrix}\square\\\square\end{smallmatrix}\right),(\square,\square,\blacksquare\blacksquare,\square)$	162	15
483	0 2 4<br>0 0 0<br>0 1 0	1 2 2	$\left(\varnothing,\begin{smallmatrix}\square\\\square\end{smallmatrix}\right),\left(\begin{smallmatrix}\square\\\square\end{smallmatrix},\varnothing\right),\left(\begin{smallmatrix}\square\\\square\end{smallmatrix},\varnothing\right),\left(\begin{smallmatrix}\square\\\square\end{smallmatrix},\varnothing\right)$	146	16
484	0 0 0 3<br>0 0 1 0<br>0 0 0 0<br>0 3 4 0	1 1 1 1	$(\varnothing,\square,\blacksquare),(\varnothing,\square,\varnothing),(\varnothing,\square\square,\blacksquare\blacksquare),(\square,\varnothing,\varnothing)$	146	20
485	0 0 1 3<br>0 0 1 0<br>0 0 0 0<br>0 3 1 0	1 1 1 1	$(\varnothing,\square\square,\varnothing),(\square,\varnothing,\blacksquare)$	144	34
486	0 0 0 4<br>0 0 1 0<br>0 0 0 0<br>0 3 1 0	1 1 1 1	$(\varnothing,\varnothing,\square\square),(\square,\square,\blacksquare)$	140	36

Continued on next page.

Continued from previous page.

Period ID	Adjacency matrix	Dimension vector	Generalized partitions	Degree	Euler Number
487	0 1 2 3<br>0 0 0 0<br>0 0 0 0<br>0 1 1 0	1 1 1 1	(□, □□, ∅)	133	35
488	0 0 2 4<br>0 0 0 0<br>0 0 0 0<br>0 2 1 0	1 1 1 1	(∅, □□, ∅),(□, ∅, □)	142	20
489	0 0 0 4<br>0 0 0 0<br>0 0 0 0<br>0 3 4 0	1 1 1 1	(∅, □, ∅),(∅, □, ∅),(□, ∅, ∅),(□, □, ■■)	136	22
490	0 0 2 3<br>0 0 0 0<br>0 0 0 0<br>0 2 2 0	1 1 1 1	(∅, □□, ∅),(□, □, ■)	130	26
491	0 0 2 4<br>0 0 0 0<br>0 0 0 0<br>0 2 1 0	1 1 1 1	(∅, □, □),(□, □, ∅)	130	34
492	0 0 0 3<br>0 0 1 0<br>0 0 0 0<br>0 3 2 0	1 1 1 1	(∅, □, ∅),(□, □, ■)	125	34
493	0 2 2 3<br>0 0 1 0<br>0 0 0 1<br>0 0 0 0	1 1 1 1	(∅, ∅, □□),(∅, □, □)	140	24
494	0 1 5<br>0 0 0<br>0 2 0	1 1 1	(∅, □□),(□□, ∅)	128	40
495	0 2 4<br>0 0 0<br>0 2 0	1 1 1	(□, □),(□□, ∅)	98	52
496	0 5<br>0 0	1 2	$\left(\begin{smallmatrix}\square\\\square\end{smallmatrix}\right),\left(\begin{smallmatrix}\square\square\\\square\square\end{smallmatrix}\right)$	160	28
497	0 0 0 2 2<br>0 0 1 0 0<br>0 0 0 0 0<br>0 0 0 0 1<br>0 2 2 0 0	1 1 1 1 1	(∅, ∅, ∅, □□),(∅, □□, ∅, ■■)	216	36

Continued on next page.

Continued from previous page.

Period ID	Adjacency matrix	Dimension vector	Generalized partitions	Degree	Euler Number
498	0 0 2 4<br>0 0 0 0<br>0 0 0 0<br>0 2 1 0	1 1 1 1	$(\varnothing,\varnothing,\square\square),(\varnothing,\square\square,\varnothing)$	224	16
499	0 0 0 3 3<br>0 0 0 0 0<br>0 0 0 0 0<br>0 0 0 0 0<br>0 2 2 0 0	1 1 1 1 1	$(\varnothing,\square,\varnothing,\square),(\square,\varnothing,\square,\blacksquare)$	240	28
500	0 0 0 3 2<br>0 0 0 0 0<br>0 0 0 0 0<br>0 0 0 0 0<br>0 2 2 1 0	1 1 1 1 1	$(\varnothing,\varnothing,\square\square,\varnothing),(\square,\varnothing,\square,\blacksquare)$	208	16
501	0 0 0 3<br>0 0 0 0<br>0 0 0 0<br>0 3 3 0	1 1 1 1	$(\square,\varnothing,\varnothing),(\square,\square\square,\blacksquare\blacksquare\blacksquare)$	192	12
502	0 0 6<br>0 0 0<br>0 2 0	1 1 1	$(\varnothing,\square\square\square),(\square,\varnothing)$	144	36
503	0 0 4 4<br>0 0 0 0<br>0 0 0 0<br>0 1 0 0	1 1 1 2	$\left(\varnothing,\varnothing,\begin{smallmatrix}\square\\\square\end{smallmatrix}\right),(\varnothing,\square,\varnothing),(\varnothing,\square\square,\varnothing),\left(\square,\varnothing,\begin{smallmatrix}\square\\\square\end{smallmatrix}\right)$	192	8
504	0 0 0 4<br>0 0 0 0<br>0 0 0 0<br>0 2 3 0	1 1 1 1	$(\varnothing,\square,\varnothing),(\square,\varnothing,\square)$	176	9
505	0 0 0 3 3<br>0 0 0 0 0<br>0 0 0 0 0<br>0 0 0 0 0<br>0 2 2 0 0	1 1 1 1 1	$(\varnothing,\varnothing,\square,\square),(\varnothing,\square,\square,\varnothing)$	192	12
506	0 0 0 2 3<br>0 0 1 0 0<br>0 0 0 0 0<br>0 0 0 0 1<br>0 2 1 0 0	1 1 1 1 1	$(\varnothing,\varnothing,\varnothing,\square\square),(\varnothing,\square,\varnothing,\varnothing)$	172	20
507	0 0 0 4<br>0 0 0 0<br>0 0 0 0<br>0 3 4 0	1 1 1 1	$(\varnothing,\varnothing,\square\square),(\varnothing,\square\square,\blacksquare\blacksquare),(\square,\varnothing,\varnothing),(\square,\square,\blacksquare\blacksquare)$	168	24

Continued on next page.

Continued from previous page.

Period ID	Adjacency matrix	Dimension vector	Generalized partitions	Degree	Euler Number
508	0 3 0<br>0 0 5<br>0 0 0	1 1 2	$(\blacksquare\blacksquare, \begin{smallmatrix}\square\\ \square\end{smallmatrix}),(\blacksquare\blacksquare, \begin{smallmatrix}\square\\ \square\end{smallmatrix}),(\blacksquare\blacksquare, \begin{smallmatrix}\square\\ \square\end{smallmatrix}),(\blacksquare, \begin{smallmatrix}\square\\ \square\end{smallmatrix})$	160	13
509	0 1 4<br>0 0 0<br>0 1 0	1 2 2	$(\begin{smallmatrix}\square\\ \square\end{smallmatrix}, \varnothing),(\begin{smallmatrix}\square\\ \square\end{smallmatrix}, \begin{smallmatrix}\square\\ \square\end{smallmatrix})$	130	31
510	0 0 0 0 3<br>0 0 0 0 0<br>0 0 0 1 0<br>0 0 0 0 0<br>0 2 3 1 0	1 1 1 1 1	$(\varnothing,\varnothing,\square,\square),(\square,\square,\varnothing,\blacksquare\blacksquare)$	176	20
511	0 0 3 3<br>0 0 0 0<br>0 0 0 1<br>0 2 0 0	1 1 1 1	$(\varnothing,\varnothing,\square\square),(\varnothing,\square,\square)$	176	4
512	0 0 2 0<br>0 0 0 0<br>0 2 0 4<br>0 1 0 0	1 1 1 2	$(\varnothing,\blacksquare\blacksquare, \begin{smallmatrix}\square\\ \square\end{smallmatrix}),(\varnothing,\blacksquare, \begin{smallmatrix}\square\\ \square\end{smallmatrix}),(\square,\blacksquare,\varnothing),(\square\square,\blacksquare\blacksquare,\varnothing)$	152	16
513	0 0 0 4<br>0 0 0 0<br>0 0 0 0<br>0 3 4 0	1 1 1 1	$(\varnothing,\varnothing,\square\square),(\varnothing,\square,\varnothing),(\square,\square,\blacksquare\blacksquare),(\square,\square,\blacksquare\blacksquare)$	152	22
514	0 0 0 0 3<br>0 0 0 1 0<br>0 0 0 0 0<br>0 0 0 0 0<br>0 2 3 1 0	1 1 1 1 1	$(\varnothing,\square,\varnothing,\varnothing),(\varnothing,\square,\square,\blacksquare)$	156	24
515	0 0 0 3<br>0 0 0 0<br>0 0 0 0<br>0 2 4 0	1 1 1 1	$(\varnothing,\square,\blacksquare),(\square,\square,\varnothing)$	144	24
516	0 0 2 0 0<br>0 0 0 0 0<br>0 2 0 2 3<br>0 0 0 0 0<br>0 0 0 1 0	1 1 1 2 2	$(\varnothing,\blacksquare\blacksquare, \begin{smallmatrix}\square\\ \square\end{smallmatrix},\varnothing),(\varnothing,\blacksquare\blacksquare, \begin{smallmatrix}\square\\ \square\end{smallmatrix},\varnothing),(\varnothing,\blacksquare, \begin{smallmatrix}\square\\ \square\end{smallmatrix},\varnothing),(\square,\blacksquare,\varnothing,\varnothing)$	141	20
517	0 1 5<br>0 0 0<br>0 1 0	1 1 2	$(\varnothing, \begin{smallmatrix}\square\\ \square\end{smallmatrix}),(\varnothing, \begin{smallmatrix}\square\\ \square\end{smallmatrix}),(\square,\square)$	131	18
518	0 0 1 5<br>0 0 0 0<br>0 0 0 0<br>0 2 1 0	1 1 1 1	$(\varnothing,\square,\square\square),(\square,\varnothing,\varnothing)$	134	38

Continued on next page.

Continued from previous page.

Period ID	Adjacency matrix	Dimension vector	Generalized partitions	Degree	Euler Number
519	0 0 2 0<br>0 0 0 0<br>0 2 0 4<br>0 1 0 0	1 1 1 2	$(\varnothing, \blacksquare\blacksquare, \yng(1,1)), (\square, \blacksquare\blacksquare\blacksquare, \yng(1,1)), (\square, \blacksquare, \varnothing), (\square, \varnothing, \varnothing)$	141	21
520	0 0 0 0 2<br>0 0 0 1 0<br>0 0 0 0 0<br>0 0 0 0 0<br>0 2 3 2 0	1 1 1 1 1	$(\varnothing, \square, \square, \blacksquare\blacksquare), (\varnothing, \square, \square, \blacksquare)$	146	25
521	0 0 4 4<br>0 0 0 0<br>0 0 0 0<br>0 1 0 0	1 1 1 2	$(\varnothing, \square, \varnothing), (\varnothing, \square, \varnothing), (\varnothing, \square, \yng(1,1)), (\square, \varnothing, \yng(1,1))$	136	22
522	0 0 0 5<br>0 0 0 0<br>0 0 0 0<br>0 3 3 0	1 1 1 1	$(\varnothing, \varnothing, \yng(2)), (\varnothing, \square, \varnothing), (\square, \varnothing, \varnothing), (\square, \square, \blacksquare\blacksquare)$	136	20
523	0 0 0 5<br>0 0 0 0<br>0 0 0 0<br>0 3 3 0	1 1 1 1	$(\varnothing, \square, \varnothing), (\varnothing, \square, \varnothing), (\square, \varnothing, \varnothing), (\square, \varnothing, \varnothing)$	131	22
524	0 0 4 4<br>0 0 0 0<br>0 0 0 0<br>0 1 0 0	1 1 1 2	$(\varnothing, \varnothing, \yng(2,2)), (\varnothing, \square, \varnothing), (\varnothing, \square, \varnothing), (\square, \square, \varnothing)$	136	8
525	0 0 0 4<br>0 0 1 0<br>0 0 0 0<br>0 3 1 0	1 1 1 1	$(\varnothing, \square, \square), (\square, \varnothing, \varnothing)$	124	27
526	0 4 4 3<br>0 0 0 0<br>0 0 0 0<br>0 0 0 0	1 1 1 2	$(\varnothing, \square, \varnothing), (\square, \varnothing, \yng(1,1)), (\square, \square, \square)$	116	25
527	0 3 4<br>0 0 1<br>0 0 0	1 1 2	$(\varnothing, \yng(1,1)), (\varnothing, \yng(1,1)), (\varnothing, \yng(1,1)), (\varnothing, \yng(1,1))$	114	23
528	0 0 5<br>0 0 0<br>0 3 0	1 1 1	$(\square, \blacksquare), (\square, \yng(2))$	112	68
529	0 0 5<br>0 0 0<br>0 1 0	1 1 2	$(\varnothing, \yng(2,1)), (\square, \varnothing)$	110	23

Continued on next page.

Continued from previous page.

Period ID	Adjacency matrix	Dimension vector	Generalized partitions	Degree	Euler Number
530	0 0 5<br>0 0 0<br>0 3 0	1 1 1	(∅, □□□),(□, ■)	192	-12
531	0 0 0 4<br>0 0 0 0<br>0 0 0 0<br>0 2 3 0	1 1 1 1	(∅, ∅, □□),(□, □□, ■■■)	240	28
532	0 0 0 0 3<br>0 0 0 0 0<br>0 0 0 1 0<br>0 0 0 0 0<br>0 2 3 1 0	1 1 1 1 1	(∅, □, □, ■■),(□, ∅, ∅, □)	210	35
533	0 0 2 4<br>0 0 1 0<br>0 0 0 0<br>0 2 0 0	1 1 1 1	(∅, ∅, □□),(∅, □□, ∅)	180	40
534	0 0 1 5<br>0 0 0 0<br>0 0 0 0<br>0 2 1 0	1 1 1 1	(∅, ∅, □□□),(□, □, ■)	141	-3
535	0 0 1 4<br>0 0 0 0<br>0 0 0 0<br>0 3 1 0	1 1 1 1	(∅, □, □□),(□, ∅, ■)	176	-4
536	0 0 1 4<br>0 0 1 0<br>0 0 0 0<br>0 3 0 0	1 1 1 1	(∅, □, □□),(□, ∅, ■)	154	30
537	0 0 0 4<br>0 0 0 0<br>0 0 0 0<br>0 2 5 0	1 1 1 1	(∅, ∅, □□),(∅, □, ■),(∅, □, ∅),(∅, □□, ■■)	192	16
538	0 0 0 3 3<br>0 0 0 0 0<br>0 0 0 0 0<br>0 0 0 0 0<br>0 2 2 0 0	1 1 1 1 1	(∅, ∅, □, □□),(□, ∅, □, ■)	192	16
539	0 0 0 0 3<br>0 0 0 1 0<br>0 0 0 0 0<br>0 0 0 0 0<br>0 2 3 1 0	1 1 1 1 1	(∅, ∅, □, ∅),(∅, □□, ∅, ■)	162	19

Continued on next page.

Continued from previous page.

Period ID	Adjacency matrix	Dimension vector	Generalized partitions	Degree	Euler Number
540	$\begin{matrix}0&1&2&4\\0&0&0&0\\0&1&0&0\\0&1&0&0\end{matrix}$	1 1 1 1	$(\varnothing,\varnothing,\square\square),(\square\square,\varnothing,\varnothing)$	156	24
541	$\begin{matrix}0&0&0&0&2\\0&0&0&1&0\\0&0&0&0&0\\0&0&0&0&0\\0&2&4&1&0\end{matrix}$	1 1 1 1 1	$(\varnothing,\square,\square,\blacksquare),(\varnothing,\square\square,\varnothing,\blacksquare\blacksquare)$	156	28
542	$\begin{matrix}0&0&2&2&2\\0&0&0&0&0\\0&0&0&0&1\\0&0&0&0&1\\0&2&0&0&0\end{matrix}$	1 1 1 1 1	$(\varnothing,\varnothing,\varnothing,\square\square),(\square,\varnothing,\varnothing,\varnothing)$	146	24
543	$\begin{matrix}0&0&2&4\\0&0&0&0\\0&0&0&0\\0&2&1&0\end{matrix}$	1 1 1 1	$(\varnothing,\square,\square\square),(\square,\square,\blacksquare)$	125	29
544	$\begin{matrix}0&0&3&3\\0&0&1&0\\0&0&0&0\\0&2&0&0\end{matrix}$	1 1 1 1	$(\varnothing,\square,\square),(\varnothing,\square\square,\varnothing)$	134	34
545	$\begin{matrix}0&0&1&4\\0&0&0&0\\0&0&0&0\\0&3&1&0\end{matrix}$	1 1 1 1	$(\varnothing,\varnothing,\square\square),(\square\square,\square,\blacksquare\blacksquare)$	160	24
546	$\begin{matrix}0&0&0&3&2\\0&0&0&0&0\\0&0&0&0&0\\0&0&0&0&1\\0&2&2&0&0\end{matrix}$	1 1 1 1 1	$(\varnothing,\square,\varnothing,\square),(\square,\varnothing,\square,\blacksquare)$	155	24
547	$\begin{matrix}0&3&4\\0&0&0\\0&1&0\end{matrix}$	1 1 2	$(\square,\square),(\square,\square)$	116	21
548	$\begin{matrix}0&0&0&0&2\\0&0&0&1&0\\0&0&0&0&0\\0&0&0&0&0\\0&2&3&2&0\end{matrix}$	1 1 1 1 1	$(\varnothing,\varnothing,\square,\varnothing),(\varnothing,\square\square,\square,\blacksquare\blacksquare\blacksquare)$	146	23
549	$\begin{matrix}0&1&5\\0&0&0\\0&1&0\end{matrix}$	1 1 2	$\left(\varnothing,\begin{smallmatrix}\square\\\square\end{smallmatrix}\right),\left(\varnothing,\begin{smallmatrix}\square\\\square\end{smallmatrix}\right),\left(\varnothing,\begin{smallmatrix}\square\\\square\end{smallmatrix}\right),(\square\square,\varnothing)$	126	16

Continued on next page.

Continued from previous page.

Period ID	Adjacency matrix	Dimension vector	Generalized partitions	Degree	Euler Number
550	0 0 0 3<br>0 0 1 0<br>0 0 0 0<br>0 3 2 0	1 1 1 1	(∅, □, □), (□, □, ■■)	130	23
551	0 0 0 3 3<br>0 0 0 0 0<br>0 0 0 0 0<br>0 0 0 0 0<br>0 2 2 0 0	1 1 1 1 1	(∅, □, □, ∅), (□, ∅, □, ∅)	126	27
552	0 5 5<br>0 0 0<br>0 0 0	1 1 2	(∅, ⊟), (∅, ⊟), (□, □), (□, □)	116	19
553	0 2 2 3<br>0 0 1 0<br>0 0 0 0<br>0 0 1 0	1 1 1 1	(∅, □, □), (∅, □□, ∅)	120	26
554	0 4 4<br>0 0 0<br>0 0 0	1 1 2	(□, □□)	90	18
555	0 0 0 4<br>0 0 2 0<br>0 0 0 0<br>0 1 0 0	1 1 1 2	(∅, ∅, ⊟), (∅, □, ⊟)	120	32
556	0 0 0 3<br>0 0 1 0<br>0 0 0 0<br>0 3 2 0	1 1 1 1	(∅, □□, ■), (□, ∅, ∅)	119	34
557	0 0 0 2<br>0 0 2 0<br>0 0 0 0<br>0 3 2 0	1 1 1 1	(∅, □, ■), (∅, □□, ■)	113	45
558	0 1 4<br>0 0 0<br>0 1 0	1 2 2	(∅, ⊟), (⊞, ∅)	114	32
559	0 0 5<br>0 0 0<br>0 1 0	1 1 2	(∅, ⊟), (∅, ⊟), (□, ⊟)	110	30

Continued on next page.

Continued from previous page.

Period ID	Adjacency matrix	Dimension vector	Generalized partitions	Degree	Euler Number
560	0 0 0 4<br>0 0 0 0<br>0 0 0 0<br>0 3 4 0	1 1 1 1	(∅, □, ■), (∅, □, ∅), (□, ∅, ∅), (□, □, ■)	110	34
561	0 0 3 3<br>0 0 0 0<br>0 0 0 1<br>0 2 0 0	1 1 1 1	(∅, ∅, □□), (□, □, ∅)	110	34
562	0 3 3<br>0 0 1<br>0 0 0	1 1 2	(∅, □□/□)	99	33
563	0 0 2 4<br>0 0 0 0<br>0 0 0 0<br>0 2 1 0	1 1 1 1	(∅, □□, □), (□, ∅, ∅)	113	40
564	0 0 1 4<br>0 0 0 0<br>0 0 0 0<br>0 3 1 0	1 1 1 1	(□, ∅, ■), (□, □, □)	103	63
565	0 0 0 3<br>0 0 1 0<br>0 0 0 0<br>0 3 2 0	1 1 1 1	(∅, □, ■), (□, □, ∅)	98	55
566	0 0 5<br>0 0 0<br>0 1 0	1 1 2	(∅, □/□), (∅, □□/□□), (□, ∅)	94	36
567	0 1 4<br>0 0 0<br>0 2 0	1 1 1	(□□, □)	81	101
568	0 8<br>0 0	1 1	(□□), (□□), (□□)	128	48
569	0 1 7<br>0 0 0<br>0 1 0	1 1 1	(∅, □□), (∅, □□), (□, □)	124	36
570	0 0 0 0 2<br>0 0 0 1 0<br>0 0 0 0 0<br>0 0 0 0 0<br>0 2 3 2 0	1 1 1 1 1	(∅, ∅, □□, ■■), (∅, □□, ∅, ■)	180	42
571	0 2 6<br>0 0 0<br>0 1 0	1 1 1	(∅, □□), (□, □), (□, □)	114	42

Continued on next page.

Continued from previous page.

Period ID	Adjacency matrix	Dimension vector	Generalized partitions	Degree	Euler Number
572	0 0 0 4 0<br>0 0 0 0 0<br>0 0 0 0 0<br>0 0 0 0 2<br>0 2 2 0 0	1 1 1 1 1	(∅, □, □□, ■), (□, ∅, □, ■)	176	12
573	0 0 0 4<br>0 0 0 0<br>0 0 0 0<br>0 2 5 0	1 1 1 1	(∅, □, ■), (∅, □, ∅), (∅, □, ∅), (∅, □, ∅)	160	0
574	0 0 0 0 2<br>0 0 0 1 0<br>0 0 0 0 0<br>0 0 0 0 0<br>0 2 4 1 0	1 1 1 1 1	(∅, □, □, ■■), (∅, □□, ∅, ■)	146	16
575	0 0 0 5<br>0 0 0 0<br>0 0 0 0<br>0 2 4 0	1 1 1 1	(∅, ∅, □□), (∅, □, ■), (∅, □, ∅), (∅, □, ∅)	160	4
576	0 0 0 5<br>0 0 0 0<br>0 0 0 0<br>0 2 4 0	1 1 1 1	(∅, ∅, □□), (∅, □, ∅), (∅, □□, ■■), (□, ∅, ∅)	136	24
577	0 0 0 0 2<br>0 0 0 1 0<br>0 0 0 0 0<br>0 0 0 0 0<br>0 2 3 2 0	1 1 1 1 1	(∅, □, ∅, ∅), (∅, □, □□, ■■■)	140	26
578	0 0 0 4<br>0 0 0 0<br>0 0 0 0<br>0 3 4 0	1 1 1 1	(∅, ∅, □□), (∅, □, ■), (∅, □, ∅), (□□, □, ■■■)	132	22
579	0 0 4<br>0 0 0<br>0 2 0	1 1 2	(□, ∅), (□, ∅), $\left(\square, \begin{smallmatrix}\square\\\square\end{smallmatrix}\right)$	101	31
580	0 0 0 3<br>0 0 1 0<br>0 0 0 0<br>0 3 2 0	1 1 1 1	(∅, ∅, □□), (□, □□, ■■■)	144	-8
581	0 0 0 4<br>0 0 0 0<br>0 0 0 0<br>0 3 4 0	1 1 1 1	(∅, ∅, □□), (∅, □, ■), (□, □, ■■), (□, □, ■)	120	36

Continued on next page.

Continued from previous page.

Period ID	Adjacency matrix	Dimension vector	Generalized partitions	Degree	Euler Number
582	0 0 5<br>0 0 0<br>0 1 0	1 1 3	$(\varnothing,\begin{smallmatrix}\square\\\square\\\square\end{smallmatrix}),(\varnothing,\begin{smallmatrix}\square\\\square\\\square\end{smallmatrix}),(\varnothing,\begin{smallmatrix}\square\\\square\\\square\end{smallmatrix}),(\square\square,\varnothing)$	116	16
583	0 4 4<br>0 0 0<br>0 0 0	1 2 2	$(\square,\begin{smallmatrix}\square\\\square\end{smallmatrix}),(\begin{smallmatrix}\square\\\square\end{smallmatrix},\square)$	106	21
584	0 0 0 5<br>0 0 0 0<br>0 0 0 0<br>0 2 4 0	1 1 1 1	$(\varnothing,\square,\varnothing),(\varnothing,\square,\varnothing),(\varnothing,\square,\varnothing),(\square,\varnothing,\varnothing)$	115	27
585	0 0 0 4<br>0 0 0 0<br>0 0 0 0<br>0 3 4 0	1 1 1 1	$(\varnothing,\square,\blacksquare),(\varnothing,\square\square,\blacksquare),(\square,\varnothing,\varnothing),(\square,\varnothing,\varnothing)$	116	22
586	0 3 5<br>0 0 0<br>0 1 0	1 1 1	$(\square,\square),(\square,\square),(\square,\square)$	99	51
587	0 0 0 5<br>0 0 0 0<br>0 0 0 0<br>0 2 4 0	1 1 1 1	$(\varnothing,\varnothing,\square\square),(\varnothing,\square,\varnothing),(\varnothing,\square,\varnothing),(\square,\square,\blacksquare\blacksquare)$	120	24
588	0 0 0 4<br>0 0 0 0<br>0 0 0 0<br>0 3 4 0	1 1 1 1	$(\varnothing,\square,\blacksquare),(\varnothing,\square,\square),(\square,\varnothing,\varnothing),(\square,\square,\blacksquare\blacksquare)$	110	26
589	0 3 5<br>0 0 0<br>0 0 0	1 2 2	$(\square,\begin{smallmatrix}\square\\\square\end{smallmatrix}),(\square,\begin{smallmatrix}\square\\\square\end{smallmatrix})$	85	28
590	0 2 6<br>0 0 0<br>0 1 0	1 1 1	$(\varnothing,\square\square),(\varnothing,\square\square),(\square\square,\varnothing)$	120	24
591	0 0 2 0<br>0 0 0 0<br>0 2 0 5<br>0 0 0 0	1 1 1 2	$(\varnothing,\blacksquare\blacksquare,\begin{smallmatrix}\square\\\square\end{smallmatrix}),(\varnothing,\blacksquare\blacksquare,\begin{smallmatrix}\square\\\square\end{smallmatrix}),(\varnothing,\blacksquare\blacksquare,\begin{smallmatrix}\square\\\square\end{smallmatrix}),(\varnothing,\blacksquare,\begin{smallmatrix}\square\\\square\end{smallmatrix})$	160	8
592	0 0 2 4<br>0 0 0 0<br>0 0 0 0<br>0 2 1 0	1 1 1 1	$(\varnothing,\varnothing,\square\square),(\square,\square\square,\blacksquare)$	134	36
593	0 1 2 0<br>0 0 0 0<br>0 1 0 5<br>0 0 0 0	1 1 1 2	$(\varnothing,\blacksquare\blacksquare,\begin{smallmatrix}\square\\\square\end{smallmatrix}),(\varnothing,\blacksquare\blacksquare,\begin{smallmatrix}\square\\\square\end{smallmatrix}),(\varnothing,\blacksquare\blacksquare,\begin{smallmatrix}\square\\\square\end{smallmatrix}),(\square,\blacksquare\blacksquare,\begin{smallmatrix}\square\\\square\end{smallmatrix})$	130	20

Continued on next page.

Continued from previous page.

Period ID	Adjacency matrix	Dimension vector	Generalized partitions	Degree	Euler Number
594	0 0 0 3 2<br>0 0 0 0 0<br>0 0 0 0 0<br>0 0 0 0 0<br>0 2 2 1 0	1 1 1 1 1	$(\varnothing,\square,\square,\blacksquare),(\square,\varnothing,\square\square,\blacksquare)$	125	28
595	0 0 2 3<br>0 0 1 0<br>0 0 0 0<br>0 3 0 0	1 1 1 1	$(\varnothing,\square\square,\square),(\square,\varnothing,\blacksquare)$	112	56
596	0 3 5<br>0 0 0<br>0 1 0	1 1 1	$(\varnothing,\square\square),(\square,\square),(\square\square,\varnothing)$	104	40
597	0 2 4<br>0 0 0<br>0 1 0	1 1 2	$(\square,\square),\left(\square,\begin{smallmatrix}\square\\\square\end{smallmatrix}\right)$	100	32
598	0 0 0 6<br>0 0 0 0<br>0 0 0 0<br>0 2 3 0	1 1 1 1	$(\varnothing,\varnothing,\square\square),(\varnothing,\square,\varnothing),(\varnothing,\square,\varnothing),(\square,\varnothing,\varnothing)$	110	26
599	0 0 3 3<br>0 0 0 0<br>0 0 0 1<br>0 2 0 0	1 1 1 1	$(\varnothing,\square,\square),(\square,\varnothing,\square)$	104	37
600	0 3 4<br>0 0 0<br>0 1 0	1 1 2	$\left(\varnothing,\begin{smallmatrix}\square\\\square\end{smallmatrix}\right),(\square,\square),(\square\square,\varnothing)$	100	24
601	0 0 0 4<br>0 0 0 0<br>0 0 0 0<br>0 2 5 0	1 1 1 1	$(\varnothing,\square,\blacksquare),(\varnothing,\square,\varnothing),(\varnothing,\square,\varnothing),(\square,\square,\blacksquare)$	100	36
602	0 0 3 3<br>0 0 0 0<br>0 0 0 1<br>0 2 0 0	1 1 1 1	$(\varnothing,\square,\square\square),(\square,\varnothing,\varnothing)$	99	40
603	0 3 3<br>0 0 1<br>0 0 0	1 2 2	$\left(\varnothing,\begin{smallmatrix}\square\\\square\end{smallmatrix}\right),\left(\varnothing,\begin{smallmatrix}\square\\\square\end{smallmatrix}\right),\left(\varnothing,\begin{smallmatrix}\square\\\square\end{smallmatrix}\right),\left(\varnothing,\begin{smallmatrix}\square\\\square\end{smallmatrix}\right)$	100	22
604	0 0 5<br>0 0 0<br>0 1 0	1 1 3	$\left(\varnothing,\begin{smallmatrix}\square\\\square\\\square\end{smallmatrix}\right),\left(\varnothing,\begin{smallmatrix}\square\\\square\\\square\end{smallmatrix}\right),(\square,\varnothing),\left(\square,\begin{smallmatrix}\square\\\square\\\square\end{smallmatrix}\right)$	95	31

Continued on next page.

Continued from previous page.

Period ID	Adjacency matrix	Dimension vector	Generalized partitions	Degree	Euler Number
605	0 0 0 3<br>0 0 0 0<br>0 0 0 0<br>0 3 3 0	1 1 1 1	(□, □, ■), (□, □, ■)	90	45
606	0 4 4<br>0 0 1<br>0 0 0	1 1 1	(∅, □□), (□, □), (□, □)	84	52
607	0 0 0 3 3<br>0 0 0 0 0<br>0 0 0 0 0<br>0 0 0 0 0<br>0 2 2 0 0	1 1 1 1 1	(∅, □, ∅, □), (□, ∅, □□, ■)	150	49
608	0 0 5<br>0 0 0<br>0 3 0	1 1 1	(∅, □□), (∅, □□)	216	24
609	0 3 4<br>0 0 1<br>0 0 0	1 1 1	(∅, □□), (∅, □□)	132	12
610	0 0 0 4<br>0 0 0 0<br>0 0 0 0<br>0 2 5 0	1 1 1 1	(∅, ∅, □□), (∅, □, ■), (∅, □□, ■■), (□, □, ■)	120	40
611	0 0 0 4<br>0 0 0 0<br>0 0 0 0<br>0 2 5 0	1 1 1 1	(∅, □, ■), (∅, □, ∅), (∅, □□, ■■), (□, ∅, □)	116	20
612	0 6 3<br>0 0 0<br>0 0 0	1 1 2	(□, ∅), (□□, □)	113	-11
613	0 0 0 3<br>0 0 0 0<br>0 0 0 0<br>0 3 3 0	1 1 1 1	(∅, □, □), (□□, □, ■■■)	102	21
614	0 1 5<br>0 0 0<br>0 1 0	1 1 4	(□, □/□/□)	86	34
615	0 0 0 5<br>0 0 0 0<br>0 0 0 0<br>0 2 4 0	1 1 1 1	(∅, ∅, □□), (∅, □, ■), (∅, □, ∅), (□, □, ■)	100	34
616	0 5 4<br>0 0 0<br>0 0 0	1 1 2	(∅, ⊞), (□, □), (□□, ∅)	96	8

Continued on next page.

Continued from previous page.

Period ID	Adjacency matrix	Dimension vector	Generalized partitions	Degree	Euler Number
617	0 3 5<br>0 0 0<br>0 0 0	1 1 2	$(\varnothing, \ydiagram{2,1})$,(□,□)	90	25
618	0 0 0 5<br>0 0 1 0<br>0 0 0 0<br>0 2 1 0	1 1 1 1	(∅,∅,□□),(∅,∅,□□)	192	32
619	0 0 3<br>0 0 0<br>0 7 0	1 1 1	(□,■),(□,∅),(□□,■■),(□□,■■)	128	4
620	0 0 0 4<br>0 0 0 0<br>0 0 0 0<br>0 2 3 0	1 1 1 1	(∅,∅,□□),(∅,□□,■)	144	4
621	0 0 5<br>0 0 0<br>0 3 0	1 1 1	(∅,□□),(□,□)	112	24
622	0 0 0 3 3<br>0 0 0 0 0<br>0 0 0 0 0<br>0 0 0 0 0<br>0 2 2 0 0	1 1 1 1 1	(∅,□,□□,∅),(□,∅,∅,∅)	114	31
623	0 0 2 0<br>0 0 0 0<br>0 2 0 5<br>0 0 0 0	1 1 1 2	$(\varnothing,■■,\ydiagram{1,1})$,$(\varnothing,■■,\ydiagram{1,1})$,$(\varnothing,■,\ydiagram{1,1})$,$(□,■■■,\ydiagram{1,1})$	105	25
624	0 0 0 5<br>0 0 0 0<br>0 0 0 0<br>0 2 4 0	1 1 1 1	(∅,□,■),(∅,□,∅),(∅,□,∅),(□,∅,□)	95	29
625	0 5 4<br>0 0 0<br>0 0 0	1 1 2	(□,□),$(□,\ydiagram{1,1})$,$(□,\ydiagram{1,1})$	85	42
626	0 2 4<br>0 0 0<br>0 1 0	1 1 2	$(\varnothing,\ydiagram{1,1})$,$(□,\ydiagram{1,1})$,(□□,∅)	90	32
627	0 0 0 2<br>0 0 1 0<br>0 0 0 0<br>0 3 3 0	1 1 1 1	(∅,□,■),(□,□□,■■)	83	72
628	0 3 4<br>0 0 0<br>0 1 0	1 1 3	$(□,\ydiagram{1,1})$,(□□,∅)	80	36

Continued on next page.

Continued from previous page.

Period ID	Adjacency matrix	Dimension vector	Generalized partitions	Degree	Euler Number
629	0 3 5<br>0 0 0<br>0 0 0	1 2 2	$\left(\varnothing,\begin{smallmatrix}\square\\\square\end{smallmatrix}\right),\left(\square,\begin{smallmatrix}\square\\\square\end{smallmatrix}\right),\left(\begin{smallmatrix}\square\\\square\end{smallmatrix},\begin{smallmatrix}\square\\\square\end{smallmatrix}\right)$	80	35
630	0 7<br>0 0	1 1	$(\square\square),(\square\square\square)$	96	90
631	0 1 6<br>0 0 0<br>0 1 0	1 1 1	$(\varnothing,\square\square\square),(\square,\square)$	93	51
632	0 1 6<br>0 0 0<br>0 1 0	1 1 1	$(\varnothing,\square\square),(\square,\square\square)$	92	78
633	0 0 4<br>0 0 0<br>0 6 0	1 1 1	$(\varnothing,\square\square),(\square,\blacksquare),(\square\square,\blacksquare\blacksquare),(\square\square,\blacksquare\blacksquare)$	192	32
634	0 0 0 5<br>0 0 1 0<br>0 0 0 0<br>0 3 2 0	1 1 1 1	$(\varnothing,\varnothing,\square\square),(\varnothing,\varnothing,\square\square),(\varnothing,\square,\blacksquare),(\square,\square,\blacksquare\blacksquare)$	168	40
635	0 2 5<br>0 0 0<br>0 1 0	1 1 1	$(\square,\square),(\square,\square\square)$	83	77
636	0 0 0 4<br>0 0 0 0<br>0 0 0 0<br>0 2 3 0	1 1 1 1	$(\varnothing,\square,\varnothing),(\varnothing,\square,\square)$	128	-8
637	0 0 2 0<br>0 0 0 0<br>0 2 0 5<br>0 0 0 0	1 1 1 2	$\left(\varnothing,\blacksquare\blacksquare,\begin{smallmatrix}\square\\\square\end{smallmatrix}\right),\left(\varnothing,\blacksquare\blacksquare,\begin{smallmatrix}\square\\\square\end{smallmatrix}\right),\left(\varnothing,\blacksquare\blacksquare,\begin{smallmatrix}\square\\\square\end{smallmatrix}\right),\left(\square,\blacksquare\blacksquare,\begin{smallmatrix}\square\\\square\end{smallmatrix}\right)$	100	32
638	0 0 0 4<br>0 0 1 0<br>0 0 0 0<br>0 2 2 0	1 1 1 1	$(\varnothing,\varnothing,\square\square),(\varnothing,\square\square,\blacksquare)$	104	40
639	0 0 0 3<br>0 0 1 0<br>0 0 0 0<br>0 2 3 0	1 1 1 1	$(\varnothing,\square,\square),(\varnothing,\square\square,\blacksquare\blacksquare)$	100	26

Continued on next page.

Continued from previous page.

Period ID	Adjacency matrix	Dimension vector	Generalized partitions	Degree	Euler Number
640	0 0 0 4<br>0 0 1 0<br>0 0 0 0<br>0 2 2 0	1 1 1 1	(∅, □, ∅), (∅, □, □)	94	32
641	0 0 0 3<br>0 0 0 0<br>0 0 0 0<br>0 3 3 0	1 1 1 1	(□, □, ■■), (□, □, ∅)	84	54
642	0 3 3<br>0 0 1<br>0 0 0	1 1 2	$\left(\varnothing, \begin{smallmatrix}\square\\\square\end{smallmatrix}\right), \left(\varnothing, \begin{smallmatrix}\square\square\\\square\square\end{smallmatrix}\right)$	82	54
643	0 2 5<br>0 0 0<br>0 1 0	1 1 1	(∅, □□□), (□□, ∅)	90	12
644	0 0 0 5<br>0 0 0 0<br>0 0 0 0<br>0 2 4 0	1 1 1 1	(∅, ∅, □□), (∅, ∅, □□), (∅, □□, ■■), (□, □, ■■)	144	48
645	0 2 5<br>0 0 0<br>0 1 0	1 1 1	(∅, □□), (□□, □)	82	84
646	0 0 0 3<br>0 0 1 0<br>0 0 0 0<br>0 2 3 0	1 1 1 1	(∅, □, ∅), (∅, □□, ■)	89	40
647	0 3 4<br>0 0 0<br>0 1 0	1 1 1	(□, □□), (□□, ∅)	74	68
648	0 3 4<br>0 0 0<br>0 1 0	1 1 1	(□, □), (□□, □)	68	93
649	0 1 4 2<br>0 0 1 0<br>0 0 0 0<br>0 1 0 0	1 1 1 1	(∅, □□, ∅), (∅, □□, ∅)	108	20
650	0 0 0 4<br>0 0 0 0<br>0 0 0 0<br>0 2 3 0	1 1 1 1	(∅, □□, ■), (□, ∅, □)	86	21
651	0 0 5<br>0 0 0<br>0 5 0	1 1 1	(∅, □□), (□, ∅), (□, ∅), (□□, ■■)	80	40

Continued on next page.

Continued from previous page.

Period ID	Adjacency matrix	Dimension vector	Generalized partitions	Degree	Euler Number
652	0 0 0 4<br>0 0 0 0<br>0 0 0 0<br>0 2 3 0	1 1 1 1	(∅, □, □),(□, □, ■)	80	40
653	0 3 0<br>0 0 5<br>0 0 0	1 1 2	(■■, $\begin{smallmatrix}\square\\\square\end{smallmatrix}$),(■■, $\begin{smallmatrix}\square\\\square\end{smallmatrix}$),(■, $\begin{smallmatrix}\square\\\square\end{smallmatrix}$),(■, $\begin{smallmatrix}\square\\\square\end{smallmatrix}$)	75	42
654	0 3 3<br>0 0 2<br>0 0 0	1 1 1	(∅, □□),(∅, □□)	68	64
655	0 0 0 5<br>0 0 0 0<br>0 0 0 0<br>0 2 4 0	1 1 1 1	(∅, ∅, □□),(∅, ∅, □□),(∅, □, ■),(□, □□, ■■■)	120	56
656	0 0 0 2<br>0 0 1 0<br>0 0 0 0<br>0 2 4 0	1 1 1 1	(∅, □□, ■■),(∅, □□, ■■)	128	0
657	0 0 0 3<br>0 0 0 0<br>0 0 0 0<br>0 2 6 0	1 1 1 1	(∅, □, ∅),(∅, □□, ■■),(∅, □□, ■■),(□, ∅, ∅)	104	12
658	0 0 5<br>0 0 0<br>0 5 0	1 1 1	(□, ∅),(□, ∅),(□, ∅),(□, ∅)	70	60
659	0 0 4<br>0 0 0<br>0 6 0	1 1 1	(□, ∅),(□, ∅),(□, ∅),(□□, ■■)	70	52
660	0 0 0 4<br>0 0 0 0<br>0 0 0 0<br>0 2 3 0	1 1 1 1	(∅, ∅, □□),(□, □□, ■■)	84	58
661	0 0 0 4<br>0 0 0 0<br>0 0 0 0<br>0 2 3 0	1 1 1 1	(∅, □, ∅),(□, □, ∅)	74	64
662	0 3 0<br>0 0 5<br>0 0 0	1 1 2	(■■■■, $\begin{smallmatrix}\square&\square\\\square&\square\end{smallmatrix}$),(■■, $\begin{smallmatrix}\square\\\square\end{smallmatrix}$),(∅, □)	74	38
663	0 3 5<br>0 0 0<br>0 0 0	1 1 3	(∅, $\begin{smallmatrix}\square&\square\\\square&\\\square&\end{smallmatrix}$),(□□, ∅)	112	-12

Continued on next page.

Continued from previous page.

Period ID	Adjacency matrix	Dimension vector	Generalized partitions	Degree	Euler Number
664	0 1 4<br>0 0 0<br>0 1 0	1 1 2	$\left(\varnothing,\begin{smallmatrix}\square\\\square\end{smallmatrix}\right),\left(\square\square,\begin{smallmatrix}\square\\\square\end{smallmatrix}\right)$	69	68
665	0 0 4<br>0 0 0<br>0 1 0	1 1 2	$\left(\square,\begin{smallmatrix}\square\square\\\square\square\end{smallmatrix}\right)$	64	77
666	0 4 4<br>0 0 1<br>0 0 0	1 1 1	$(\varnothing,\square\square),(\varnothing,\square\square),(\square\square,\varnothing)$	88	40
667	0 2 2 3<br>0 0 0 1<br>0 0 0 1<br>0 0 0 0	1 1 1 1	$(\varnothing,\varnothing,\square\square),(\varnothing,\varnothing,\square\square)$	84	32
668	0 0 0 3<br>0 0 6 0<br>0 0 0 0<br>0 1 0 0	1 1 1 2	$(\blacksquare\blacksquare,\square\square,\square),(\blacksquare,\square,\varnothing),(\square,\varnothing,\varnothing)$	89	-3
669	0 3 0<br>0 0 5<br>0 0 0	1 1 2	$\left(\blacksquare\blacksquare,\begin{smallmatrix}\square\\\square\end{smallmatrix}\right),\left(\blacksquare\blacksquare,\begin{smallmatrix}\square\\\square\end{smallmatrix}\right),\left(\blacksquare\blacksquare,\begin{smallmatrix}\square\\\square\end{smallmatrix}\right),\left(\varnothing,\begin{smallmatrix}\square\\\square\end{smallmatrix}\right)$	70	49
670	0 3 5<br>0 0 1<br>0 0 0	1 1 1	$(\varnothing,\square\square),(\varnothing,\square\square),(\square,\square)$	64	56
671	0 7 0<br>0 0 3<br>0 0 0	1 1 2	$(\varnothing,\square),(\square,\square)$	55	59
672	0 0 0 5<br>0 0 0 0<br>0 0 0 0<br>0 2 2 0	1 1 1 1	$(\varnothing,\varnothing,\square\square),(\square,\varnothing,\square)$	112	-8
673	0 0 4<br>0 0 0<br>0 6 0	1 1 1	$(\varnothing,\square\square),(\square,\varnothing),(\square\square,\blacksquare\blacksquare),(\square\square,\blacksquare\blacksquare)$	80	24
674	0 0 0 5<br>0 0 1 0<br>0 0 0 0<br>0 2 1 0	1 1 1 1	$(\varnothing,\varnothing,\square\square),(\varnothing,\square,\square)$	88	32
675	0 0 5<br>0 0 0<br>0 5 0	1 1 1	$(\varnothing,\square\square),(\varnothing,\square\square),(\square\square,\blacksquare\blacksquare),(\square\square,\blacksquare\blacksquare)$	96	64

Continued on next page.

Continued from previous page.

Period ID	Adjacency matrix	Dimension vector	Generalized partitions	Degree	Euler Number
676	0 0 0 2<br>0 0 1 0<br>0 0 0 0<br>0 2 4 0	1 1 1 1	(∅, □□, ■■),(∅, □□, ■)	74	42
677	0 0 5<br>0 0 0<br>0 5 0	1 1 1	(∅, □□),(□, ■),(□, ∅),(□□, ■)	60	68
678	0 0 3<br>0 0 0<br>0 7 0	1 1 1	(□, ∅),(□, ∅),(□□, ■■),(□□, ■■)	60	48
679	0 3 0<br>0 0 4<br>0 0 0	1 1 2	(■■, □□/□)	56	61
680	0 3 4<br>0 0 1<br>0 0 0	1 1 1	(∅, □□),(□, □□)	54	94
681	0 0 0 3<br>0 0 0 0<br>0 0 0 0<br>0 2 4 0	1 1 1 1	(∅, □, ■),(∅, □□, ∅)	96	-24
682	0 0 4<br>0 0 0<br>0 6 0	1 1 1	(□, ■),(□, ∅),(□, ∅),(□□, ■)	55	85
683	0 6<br>0 0	1 1	(□□□□)	64	188
684	0 1 5<br>0 0 0<br>0 1 0	1 1 1	(□, □□□)	61	149
685	0 0 0 4<br>0 0 0 0<br>0 0 0 0<br>0 2 3 0	1 1 1 1	(∅, □□, ∅),(□, ∅, ∅)	68	62
686	0 3 5<br>0 0 0<br>0 0 0	1 1 2	(∅, □/□),(∅, □□/□),(□□, ∅)	96	-20
687	0 2 4<br>0 0 0<br>0 1 0	1 1 1	(□□, □□)	52	168
688	0 0 0 5<br>0 0 0 0<br>0 0 0 0<br>0 2 2 0	1 1 1 1	(∅, □, □),(□, ∅, □)	65	37

Continued on next page.

Continued from previous page.

Period ID	Adjacency matrix	Dimension vector	Generalized partitions	Degree	Euler Number
689	0 3 3<br>0 0 0<br>0 1 0	1 1 2	(□□, □)	51	51
690	0 0 4<br>0 0 0<br>0 4 0	1 1 1	(∅, □□□), (□, ■)	162	27
691	0 3 3<br>0 0 1<br>0 0 0	1 1 1	(∅, □□□)	99	27
692	0 0 5<br>0 0 0<br>0 5 0	1 1 1	(∅, □□), (□, ■), (□, □), (□□, ■■)	64	72
693	0 0 3<br>0 0 0<br>0 7 0	1 1 1	(□, ■), (□, □), (□□, ■■), (□□, ■■)	56	40
694	0 0 0 4<br>0 0 0 0<br>0 0 0 0<br>0 2 3 0	1 1 1 1	(∅, ∅, □□□), (□, □, ■■)	144	36
695	0 0 5<br>0 0 0<br>0 3 0	1 1 1	(∅, □□□), (□, ∅)	96	-9
696	0 0 4<br>0 0 0<br>0 4 0	1 1 1	(□, ■), (□, □□)	80	35
697	0 0 4<br>0 0 0<br>0 4 0	1 1 1	(∅, □□), (□□□, ■■■)	144	36
698	0 0 0 4<br>0 0 1 0<br>0 0 0 0<br>0 3 1 0	1 1 1 1	(∅, ∅, □□□), (□, □, ■■)	126	45
699	0 2 5<br>0 0 0<br>0 0 0	1 1 2	(∅, □□/□), (□, □/□)	60	40

Continued on next page.

Continued from previous page.

Period ID	Adjacency matrix	Dimension vector	Generalized partitions	Degree	Euler Number
700	0 0 0 4<br>0 0 1 0<br>0 0 0 0<br>0 2 2 0	1 1 1 1	(∅, ∅, □□□),(∅, □□, ■■)	108	54
701	0 0 0 3<br>0 0 0 0<br>0 0 0 0<br>0 2 4 0	1 1 1 1	(∅, □, ■),(□, □□, ■)	54	105
702	0 0 3<br>0 0 0<br>0 7 0	1 1 1	(□, ■),(□, ∅),(□□, ■■),(□□, ■)	50	86
703	0 0 0 4<br>0 0 0 0<br>0 0 0 0<br>0 2 3 0	1 1 1 1	(∅, ∅, □□□),(□, □□, ■■■)	90	63
704	0 0 3 3<br>0 0 0 0<br>0 0 0 1<br>0 2 0 0	1 1 1 1	(∅, ∅, □□□),(□, □, ■)	81	36
705	0 2 3<br>0 0 0<br>0 2 0	1 1 1	(□□□, ∅)	51	135
706	0 0 0 5<br>0 0 0 0<br>0 0 0 0<br>0 2 2 0	1 1 1 1	(∅, ∅, □□□),(∅, □, ∅)	96	-12
707	0 0 0 5<br>0 0 1 0<br>0 0 0 0<br>0 2 1 0	1 1 1 1	(∅, ∅, □□□),(∅, □, ∅)	78	0
708	0 0 4<br>0 0 0<br>0 4 0	1 1 1	(□, □),(□□, ■)	46	93
709	0 0 5<br>0 0 0<br>0 5 0	1 1 1	(∅, □□),(∅, □□),(□, ■),(□□□, ■■■)	72	72
710	0 3 4<br>0 0 0<br>0 1 0	1 1 1	(∅, □□),(□□□, ∅)	66	84
711	0 0 0 2<br>0 0 1 0<br>0 0 0 0<br>0 2 4 0	1 1 1 1	(∅, □, ∅),(∅, □□□, ■■■)	63	48

Continued on next page.

Continued from previous page.

Period ID	Adjacency matrix	Dimension vector	Generalized partitions	Degree	Euler Number
712	0 3 4<br>0 0 1<br>0 0 0	1 1 1	(∅, □□□),(□, □)	48	99
713	0 0 4<br>0 0 0<br>0 6 0	1 1 1	(∅, □□),(□, ■),(□, ∅),(□□□, ■■■)	60	12
714	0 3 0<br>0 0 5<br>0 0 0	1 1 2	(■■■■, □□/□□),(■■, □/□),(■■, □/□),(□□, ∅)	80	-32
715	0 6<br>0 0	1 4	(□□/□/□/□)	42	73
716	0 0 5<br>0 0 0<br>0 3 0	1 1 1	(□, □),(□, □)	41	109
717	0 3 5<br>0 0 0<br>0 0 0	1 1 2	(∅, □/□),(∅, □□/□□),(□, ∅),(□, □/□)	50	52
718	0 0 3<br>0 0 0<br>0 7 0	1 1 1	(□, ■),(□, ∅),(□, ∅),(□□□, ■■■)	45	63
719	0 0 0 4<br>0 0 0 0<br>0 0 0 0<br>0 2 3 0	1 1 1 1	(∅, □, ■),(∅, □, □□)	80	-28
720	0 0 0 4<br>0 0 0 0<br>0 0 0 0<br>0 2 3 0	1 1 1 1	(∅, □, □□),(□, □, ■■)	62	44
721	0 0 4<br>0 0 0<br>0 4 0	1 1 1	(□, ∅),(□□, ∅)	40	152
722	0 0 0 5<br>0 0 0 0<br>0 0 0 0<br>0 2 2 0	1 1 1 1	(∅, □, ∅),(□, ∅, □□)	53	80
723	0 3 5<br>0 0 0<br>0 0 0	1 1 2	(∅, □/□),(∅, □/□),(□, ∅),(□, □□/□□)	45	94
724	0 0 5<br>0 0 0<br>0 3 0	1 1 1	(∅, □□),(□□, ∅)	40	144

Continued on next page.

Continued from previous page.

Period ID	Adjacency matrix	Dimension vector	Generalized partitions	Degree	Euler Number
725	0 3 3<br>0 0 1<br>0 0 0	1 1 1	$(\square, \square\square\square)$	38	191
726	0 0 5<br>0 0 0<br>0 3 0	1 1 1	$(\varnothing, \square\square\square), (\square\square, \blacksquare)$	42	27
727	0 0 4<br>0 0 0<br>0 4 0	1 1 1	$(\varnothing, \square\square\square), (\square\square\square, \blacksquare\blacksquare\blacksquare)$	54	81
728	0 0 4<br>0 0 0<br>0 4 0	1 1 1	$(\varnothing, \square\square), (\square\square\square, \blacksquare\blacksquare)$	44	116
729	0 0 5<br>0 0 0<br>0 3 0	1 1 1	$(\square, \varnothing), (\square, \square\square)$	35	155
730	0 6<br>0 0	1 2	$\left(\substack{\square\\\square}\right), \left(\substack{\square\\\square}\right), \left(\substack{\square\square\\\square}\right)$	33	90
731	0 0 3<br>0 0 0<br>0 7 0	1 1 1	$(\varnothing, \square\square), (\square\square, \blacksquare\blacksquare), (\square\square, \blacksquare\blacksquare), (\square\square, \blacksquare\blacksquare)$	64	-48
732	0 0 2<br>0 0 0<br>0 8 0	1 1 1	$(\square, \varnothing), (\square\square, \blacksquare\blacksquare), (\square\square, \blacksquare\blacksquare), (\square\square, \blacksquare\blacksquare)$	40	72
733	0 0 2<br>0 0 0<br>0 8 0	1 1 1	$(\square, \blacksquare), (\square\square, \blacksquare\blacksquare), (\square\square, \blacksquare\blacksquare), (\square\square, \blacksquare)$	36	92
734	0 6<br>0 0	1 2	$\left(\substack{\square\\\square}\right), \left(\substack{\square\\\square}\right), \left(\substack{\square\\\square}\right), \left(\substack{\square\square\\\square\square}\right)$	28	140
735	0 0 4<br>0 0 0<br>0 4 0	1 1 1	$(\square, \blacksquare), (\square\square, \square)$	26	251
736	0 0 6<br>0 0 0<br>0 2 0	1 1 1	$(\varnothing, \square\square), (\varnothing, \square\square\square)$	48	-72
737	0 2 5<br>0 0 1<br>0 0 0	1 1 1	$(\varnothing, \square\square), (\varnothing, \square\square\square)$	30	114
738	0 0 6<br>0 0 0<br>0 2 0	1 1 1	$(\varnothing, \square\square\square), (\square, \square)$	27	99

Continued on next page.

Continued from previous page.

Period ID	Adjacency matrix	Dimension vector	Generalized partitions	Degree	Euler Number
739	0 0 6<br>0 0 0<br>0 2 0	1 1 1	(∅, □□),(□, □□)	26	186
740	0 5<br>0 0	1 2	(□□/□□),(□□/□□)	20	176
741	0 9<br>0 0	1 1	(□□),(□□),(□□),(□□)	16	224
742	0 5<br>0 0	1 2	(□/□),(□□□/□□□)	15	318
743	0 0 5<br>0 0 0<br>0 3 0	1 1 1	(∅, □□□□),(□□, ■■)	32	-112
744	0 0 6<br>0 0 0<br>0 2 0	1 1 1	(∅, □□□□),(□, ∅)	20	212
745	0 0 5<br>0 0 0<br>0 3 0	1 1 1	(□, ■),(□, □□□)	17	293
746	0 8<br>0 0	1 1	(□□),(□□),(□□□)	12	324
747	0 7<br>0 0	1 1	(□□□),(□□□)	9	369
748	0 7<br>0 0	1 1	(□□),(□□□□)	8	552
749	0 6<br>0 0	1 1	(□□□□□)	5	825

Table 4: Some regularized period sequences obtained from 4-dimensional Fano manifolds that arise as quiver flag zero loci.

Period ID	Name	α_0	α_1	α_2	α_3	α_4	α_5	α_6	α_7
1	BØS^4_{124}, $\mathbb{P}^4$, CKP_1	1	0	0	0	0	120	0	0
2	BØS^4_{115}, CKP_2	1	0	0	0	24	120	0	0
3	CKP_3, Q^4	1	0	0	0	48	0	0	0
4	CKP_4	1	0	0	0	48	120	0	0
5	BØS^4_{118}, CKP_8	1	0	0	6	0	120	90	0
6	BØS^4_{47}, CKP_{10}	1	0	0	6	24	0	90	2520
7	BØS^4_{94}, CKP_{11}	1	0	0	6	24	120	90	1260
8	BØS^4_{37}, CKP_{12}	1	0	0	6	24	120	90	2520
9	CKP_{13}, BØS^4_{74}	1	0	0	6	48	0	90	2520
10	CKP_{14}	1	0	0	6	48	0	90	3780
11	CKP_{15}, BØS^4_{86}	1	0	0	6	48	120	90	2520
12	CKP_{16}	1	0	0	6	48	120	90	3780
13	CKP_{18}	1	0	0	6	72	120	90	5040
14	$\mathbb{P}^2 \times \mathbb{P}^2$, CKP_{20}, FI^4_6, BØS^4_{123}	1	0	0	12	0	0	900	0
15		1	0	0	12	0	120	540	0
16	BØS^4_{114}, CKP_{21}	1	0	0	12	0	120	900	0
17	CKP_{23}, BØS^4_{46}	1	0	0	12	24	0	900	3780
18	CKP_{25}, BØS^4_{32}	1	0	0	12	24	240	900	5040
19	CKP_{26}	1	0	0	12	48	0	540	7560
20		1	0	0	12	48	0	900	7560
21		1	0	0	12	48	120	540	7560
22	CKP_{29}	1	0	0	12	72	120	540	10080
23	CKP_{30}	1	0	0	12	96	120	540	15120
24	FI^4_5	1	0	0	18	0	0	1710	0
25		1	0	0	18	48	0	1710	11340
26		1	0	0	18	48	120	2430	11340
27	CKP_{33}, FI^4_4	1	0	0	24	0	0	3240	0
28	CKP_{34}	1	0	0	24	48	0	3240	15120
29		1	0	0	24	48	120	3600	15120
30	CKP_{35}	1	0	0	24	96	120	3240	30240
31	CKP_{36}	1	0	0	24	120	120	3240	40320
32	Str_1	1	0	0	30	120	240	5850	50400
33	FI^4_3, CKP_{37}	1	0	0	36	0	0	8100	0
34	CKP_{39}	1	0	0	36	144	120	8100	75600

Continued on next page.

Continued from previous page.

Period ID	Name	α_0	α_1	α_2	α_3	α_4	α_5	α_6	α_7
35	CKP_{47}, $BØS^4_{121}$	1	0	2	0	6	120	20	2520
36	$\mathbb{P}^1 \times \mathbb{P}^3$, CKP_{51}, $BØS^4_{122}$, MW^4_{14}	1	0	2	0	30	0	740	0
37	$BØS^4_{109}$, CKP_{54}	1	0	2	0	30	120	380	2520
38	CKP_{55}, $BØS^4_{104}$	1	0	2	0	30	120	740	2520
39	CKP_{60}, MW^4_{13}	1	0	2	0	54	0	740	0
40	CKP_{61}, MW^4_{12}	1	0	2	0	54	0	1100	0
41	CKP_{64}	1	0	2	0	54	120	740	2520
42	CKP_{65}	1	0	2	0	54	120	1100	2520
43	CKP_{67}	1	0	2	0	54	240	1460	5040
44	$BØS^4_{111}$, CKP_{76}	1	0	2	6	6	180	110	2940
45	CKP_{78}	1	0	2	6	6	240	110	3780
46	$BØS^4_{106}$, CKP_{79}	1	0	2	6	30	60	470	2940
47	CKP_{80}, $BØS^4_{45}$	1	0	2	6	30	60	830	2940
48	CKP_{81}, $BØS^4_{41}$	1	0	2	6	30	120	470	3780
49	$\mathbb{P}^1 \times MM^3_{2-33}$, $BØS^4_{110}$, CKP_{83}	1	0	2	6	30	120	830	2520
50	$BØS^4_{82}$, CKP_{84}	1	0	2	6	30	180	470	4200
51	CKP_{85}, $BØS^4_{113}$	1	0	2	6	30	180	470	5460
52	$BØS^4_{92}$, CKP_{86}	1	0	2	6	30	180	830	5460
53	CKP_{89}	1	0	2	6	30	240	830	5040
54	$BØS^4_{52}$, CKP_{91}	1	0	2	6	54	60	830	2940
55	CKP_{92}	1	0	2	6	54	60	830	4200
56	CKP_{93}	1	0	2	6	54	60	1190	4200
57	CKP_{96}, $BØS^4_{91}$	1	0	2	6	54	180	830	5460
58	CKP_{98}	1	0	2	6	54	180	1190	6720
59	CKP_{99}	1	0	2	6	54	180	1190	7980
60	$BØS^4_{81}$, CKP_{100}	1	0	2	6	54	240	1190	6300
61	CKP_{101}	1	0	2	6	54	240	1190	7560
62	CKP_{102}	1	0	2	6	54	360	1550	8820
63	CKP_{103}	1	0	2	6	78	180	1190	7980
64	CKP_{104}	1	0	2	6	78	360	1910	11340
65	CKP_{107}	1	0	2	6	102	600	2990	17640
66	CKP_{109}	1	0	2	12	6	120	920	840
67	$BØS^4_{112}$, CKP_{110}, $\mathbb{P}^2 \times S^2_8$	1	0	2	12	6	180	920	1680

Continued on next page.

Continued from previous page.

Period ID	Name	α_0	α_1	α_2	α_3	α_4	α_5	α_6	α_7
68	CKP_{111}, $\mathbb{P}^1 \times Q^3$	1	0	2	12	6	240	560	2520
69	CKP_{113}	1	0	2	12	6	300	920	4200
70	CKP_{114}	1	0	2	12	6	360	560	5040
71	$\mathrm{BØS}^4_{60}$, CKP_{116}	1	0	2	12	30	120	920	4620
72	$\mathrm{BØS}^4_{88}$, CKP_{117}	1	0	2	12	30	180	1280	5460
73	$\mathrm{BØS}^4_{35}$, CKP_{118}	1	0	2	12	30	180	1280	5460
74	CKP_{119}	1	0	2	12	30	180	1640	5460
75	$\mathbb{P}^1 \times \mathrm{MM}^3_{2\text{–}30}$, CKP_{120}	1	0	2	12	30	240	1280	5040
76		1	0	2	12	30	300	920	9240
77	$\mathrm{BØS}^4_{93}$, CKP_{121}	1	0	2	12	30	300	1280	7980
78		1	0	2	12	30	300	1640	7980
79	CKP_{122}	1	0	2	12	30	360	1280	7560
80	CKP_{123}	1	0	2	12	30	420	1280	11760
81	CKP_{124}	1	0	2	12	54	120	1280	8400
82	CKP_{125}	1	0	2	12	54	120	1640	8400
83	CKP_{126}	1	0	2	12	54	180	1640	9240
84	$\mathrm{BØS}^4_{85}$, CKP_{127}	1	0	2	12	54	240	1280	9660
85	CKP_{128}	1	0	2	12	54	240	1280	10080
86	CKP_{130}	1	0	2	12	54	300	2000	11760
87	CKP_{131}	1	0	2	12	54	360	1640	12600
88	CKP_{132}	1	0	2	12	54	420	2000	15540
89	CKP_{134}	1	0	2	12	78	240	2000	14700
90	CKP_{135}	1	0	2	12	78	300	2000	14280
91	CKP_{136}	1	0	2	12	78	300	2720	16800
92	CKP_{137}	1	0	2	12	78	360	2000	15120
93	CKP_{138}	1	0	2	12	78	480	2360	17640
94	CKP_{139}	1	0	2	12	102	240	2000	18480
95	CKP_{141}	1	0	2	12	102	480	2720	20160
96	CKP_{142}	1	0	2	12	102	480	2720	22680
97	CKP_{144}	1	0	2	12	126	720	3800	30240
98	CKP_{145}	1	0	2	12	198	1200	6320	52920
99	$\mathrm{BØS}^4_{51}$, CKP_{146}	1	0	2	18	6	180	1370	1260
100	CKP_{147}	1	0	2	18	6	240	1730	2100
101		1	0	2	18	6	300	1730	2940

Continued on next page.

Continued from previous page.

Period ID	Name	α_0	α_1	α_2	α_3	α_4	α_5	α_6	α_7
102		1	0	2	18	6	420	1730	5460
103	CKP_{148}	1	0	2	18	30	240	2090	7140
104		1	0	2	18	30	360	2450	9660
105	CKP_{151}, $\mathrm{BØS}^4_{73}$	1	0	2	18	54	180	2090	11340
106	CKP_{152}	1	0	2	18	54	240	2810	13440
107	CKP_{153}	1	0	2	18	78	300	2450	18900
108	CKP_{154}	1	0	2	18	78	360	3170	21000
109		1	0	2	18	102	300	3170	26460
110	CKP_{155}	1	0	2	18	102	360	3890	28560
111	CKP_{158}	1	0	2	24	6	240	3260	1680
112	CKP_{159}	1	0	2	24	6	360	3260	3360
113	CKP_{160}	1	0	2	24	6	540	3260	6720
114	CKP_{161}	1	0	2	24	54	360	3980	18480
115		1	0	2	24	54	360	4340	18480
116		1	0	2	24	54	480	4700	21000
117	CKP_{162}	1	0	2	24	54	540	4340	21840
118	CKP_{163}	1	0	2	24	102	420	4700	35280
119	CKP_{164}	1	0	2	24	102	480	4700	35280
120	CKP_{165}	1	0	2	24	126	660	5780	49560
121	CKP_{166}	1	0	2	24	150	720	6140	55440
122	CKP_{167}	1	0	2	24	174	960	7220	70560
123	CKP_{168}	1	0	2	24	246	1440	9740	105840
124	Str_2	1	0	2	30	54	600	6590	26040
125		1	0	2	30	78	960	7670	46200
126		1	0	2	30	126	540	7670	56700
127	CKP_{169}	1	0	2	36	6	360	8120	2520
128	CKP_{170}	1	0	2	36	6	720	8120	8400
129	CKP_{171}	1	0	2	36	150	840	11000	86520
130	CKP_{172}	1	0	2	36	294	1680	15320	178920
131	CKP_{174}	1	0	2	36	438	2640	20360	287280
132	CKP_{175}	1	0	2	42	150	900	14690	99540
133	$\mathrm{BØS}^4_{43}$, CKP_{181}	1	0	4	0	36	120	400	5040
134	$\mathrm{BØS}^4_{117}$, CKP_{183}, MW^4_{17}, $\mathbb{P}^1 \times \mathrm{MM}^3_{2\text{–}35}$	1	0	4	0	60	0	1480	0

Continued on next page.

Continued from previous page.

Period ID	Name	α_0	α_1	α_2	α_3	α_4	α_5	α_6	α_7
135	CKP_{185}, $\mathrm{BØS}^4_{36}$	1	0	4	0	60	120	1480	5040
136	MW^4_{11}	1	0	4	0	84	0	2200	0
137	MW^4_{10}, CKP_{186}	1	0	4	0	84	0	2560	0
138	CKP_{187}	1	0	4	0	84	240	2560	10080
139	CKP_{189}, MW^4_7	1	0	4	0	108	0	3280	0
140	$\mathrm{BØS}^4_{120}$, $\mathbb{P}^1\times\mathbb{P}^1\times\mathbb{P}^2$, $\mathbb{P}^2\times\mathbb{P}^1\times\mathbb{P}^1$, $\mathbb{P}^1\times\mathrm{MM}^3_{2\text{–}34}$, CKP_{195}	1	0	4	6	36	240	490	7560
141	CKP_{197}	1	0	4	6	36	300	490	9240
142		1	0	4	6	36	360	490	12600
143	$\mathbb{P}^1\times\mathrm{MM}^3_{3\text{–}30}$, CKP_{200}, $\mathrm{BØS}^4_{89}$	1	0	4	6	60	180	1570	5460
144	CKP_{201}, $\mathrm{BØS}^4_{34}$	1	0	4	6	60	180	1570	6720
145	CKP_{203}	1	0	4	6	60	240	1210	10080
146	$\mathbb{P}^1\times\mathrm{MM}^3_{3\text{–}26}$, CKP_{204}, $\mathrm{BØS}^4_{103}$	1	0	4	6	60	240	1570	8820
147	CKP_{205}, $\mathrm{BØS}^4_{102}$	1	0	4	6	60	240	1570	9660
148	CKP_{206}, $\mathrm{BØS}^4_{44}$	1	0	4	6	60	240	1930	9660
149	CKP_{207}	1	0	4	6	60	300	1210	11760
150	CKP_{208}	1	0	4	6	60	300	1570	10500
151	CKP_{209}	1	0	4	6	60	360	1570	13860
152	CKP_{214}	1	0	4	6	84	240	2650	10080
153	CKP_{215}	1	0	4	6	84	240	2650	12180
154		1	0	4	6	84	300	2290	13020
155	$\mathrm{BØS}^4_{29}$, CKP_{217}	1	0	4	6	84	360	2650	15120
156	CKP_{218}	1	0	4	6	84	360	3010	17220
157	CKP_{219}	1	0	4	6	84	420	2650	16800
158	CKP_{220}	1	0	4	6	108	240	3370	13860
159	CKP_{222}	1	0	4	6	108	300	3370	15540
160	CKP_{224}	1	0	4	6	132	660	4810	30660
161	$\mathbb{P}^1\times\mathrm{MM}^3_{3\text{–}31}$, CKP_{225}, $\mathrm{BØS}^4_{72}$	1	0	4	12	36	360	940	8400
162	$S^2_8\times S^2_8$, $\mathrm{BØS}^4_{83}$, CKP_{226}	1	0	4	12	36	360	1300	8400
163	CKP_{227}, $\mathrm{BØS}^4_{101}$, $\mathbb{P}^2\times S^2_7$	1	0	4	12	36	360	1300	9660
164	CKP_{228}, $\mathbb{P}^1\times\mathrm{MM}^3_{2\text{–}31}$	1	0	4	12	36	420	940	11760
165	CKP_{230}	1	0	4	12	36	480	1300	13440
166	CKP_{231}	1	0	4	12	36	480	1300	14700

Continued on next page.

Continued from previous page.

Period ID	Name	α_0	α_1	α_2	α_3	α_4	α_5	α_6	α_7
167	CKP_{233}	1	0	4	12	36	720	940	25200
168	$\mathbb{P}^1 \times \mathrm{MM}^3_{3\text{–}25}$, CKP_{236}, $\mathrm{BØS}^4_{108}$	1	0	4	12	60	360	2020	10920
169	CKP_{239}	1	0	4	12	60	360	2380	13440
170	CKP_{240}, $\mathbb{P}^1 \times \mathrm{MM}^3_{3\text{–}23}$	1	0	4	12	60	420	2020	14280
171	CKP_{243}	1	0	4	12	60	480	2020	17220
172	CKP_{244}	1	0	4	12	60	480	2380	18480
173		1	0	4	12	60	480	2740	18480
174	CKP_{246}	1	0	4	12	60	540	2020	19320
175	CKP_{247}	1	0	4	12	60	600	2020	23520
176	CKP_{248}	1	0	4	12	84	360	3100	15960
177	$\mathrm{BØS}^4_{80}$, CKP_{251}	1	0	4	12	84	420	2740	17640
178		1	0	4	12	84	420	2740	19320
179	CKP_{252}	1	0	4	12	84	420	3100	18900
180	CKP_{255}	1	0	4	12	84	480	2740	19740
181	$\mathbb{P}^1 \times \mathrm{MM}^3_{3\text{–}19}$, CKP_{256}	1	0	4	12	84	480	3100	20160
182		1	0	4	12	84	480	3100	23520
183	CKP_{258}	1	0	4	12	84	480	3460	22260
184	CKP_{259}	1	0	4	12	84	600	3460	28560
185	CKP_{260}	1	0	4	12	84	600	3820	27300
186	CKP_{262}	1	0	4	12	84	720	3100	32760
187	CKP_{265}	1	0	4	12	108	540	4180	27720
188		1	0	4	12	108	600	3820	28560
189	CKP_{266}	1	0	4	12	108	600	4180	31080
190		1	0	4	12	108	600	4900	32340
191	CKP_{267}	1	0	4	12	108	720	4180	38640
192	CKP_{268}	1	0	4	12	108	720	4900	37380
193	CKP_{269}	1	0	4	12	132	600	4900	33600
194	CKP_{270}	1	0	4	12	132	720	5260	37800
195		1	0	4	18	36	720	2110	21000
196	CKP_{277}, $\mathbb{P}^1 \times \mathrm{MM}^3_{2\text{–}27}$	1	0	4	18	60	600	2830	19740
197	CKP_{279}	1	0	4	18	60	840	3910	32340
198	CKP_{280}, $\mathrm{BØS}^4_{53}$	1	0	4	18	84	480	3190	20580
199	CKP_{282}	1	0	4	18	84	540	3910	25200

Continued on next page.

Continued from previous page.

Period ID	Name	α_0	α_1	α_2	α_3	α_4	α_5	α_6	α_7
200	CKP_{283}, $\mathrm{BØS}^{4}_{84}$	1	0	4	18	84	600	3550	25620
201		1	0	4	18	84	600	4270	26880
202		1	0	4	18	84	720	3910	32340
203	CKP_{284}	1	0	4	18	84	720	4630	32340
204	CKP_{285}	1	0	4	18	84	780	4270	34020
205	CKP_{286}	1	0	4	18	108	600	4270	30660
206	CKP_{287}	1	0	4	18	108	600	4630	31920
207	CKP_{288}	1	0	4	18	108	660	4990	34020
208	CKP_{289}	1	0	4	18	108	720	4990	38220
209	CKP_{290}	1	0	4	18	108	780	4990	39060
210	CKP_{291}	1	0	4	18	108	780	5350	40320
211		1	0	4	18	108	840	4990	44940
212		1	0	4	18	108	960	6070	49980
213	CKP_{292}	1	0	4	18	132	780	5350	42840
214	CKP_{293}	1	0	4	18	132	840	5710	48720
215	CKP_{294}	1	0	4	18	132	960	7150	55020
216	CKP_{295}	1	0	4	18	132	960	7510	57540
217	CKP_{296}	1	0	4	18	156	840	7150	56280
218	CKP_{297}	1	0	4	18	156	1020	7870	63000
219		1	0	4	18	180	1020	7870	66780
220	CKP_{298}	1	0	4	18	180	1080	9310	77700
221	CKP_{299}	1	0	4	24	36	720	3640	16800
222	CKP_{300}	1	0	4	24	36	1080	3640	33600
223	CKP_{301}	1	0	4	24	84	720	5800	31920
224		1	0	4	24	84	840	5800	38220
225	CKP_{302}	1	0	4	24	84	1080	5800	48720
226		1	0	4	24	84	1140	5800	51660
227		1	0	4	24	108	960	6880	49560
228	CKP_{303}	1	0	4	24	108	1080	6520	58800
229	CKP_{304}	1	0	4	24	132	840	6880	53340
230		1	0	4	24	132	840	7240	53760
231		1	0	4	24	132	840	7960	54600
232		1	0	4	24	132	1020	7600	60480
233	CKP_{305}	1	0	4	24	156	960	7960	63420

Continued on next page.

Continued from previous page.

Period ID	Name	α_0	α_1	α_2	α_3	α_4	α_5	α_6	α_7
234	CKP_{306}	1	0	4	24	156	1080	9040	72240
235		1	0	4	24	180	1440	11560	99120
236	CKP_{307}	1	0	4	24	204	1260	10480	95760
237	CKP_{308}	1	0	4	24	228	1440	12280	110880
238		1	0	4	24	276	1680	13720	137760
239	CKP_{309}	1	0	4	30	84	840	6610	36540
240	CKP_{310}	1	0	4	30	84	1200	8050	54600
241	CKP_{311}	1	0	4	30	132	960	8770	61740
242		1	0	4	30	132	1140	9490	70560
243	CKP_{312}	1	0	4	30	156	1320	11650	92400
244		1	0	4	30	204	1440	12730	113820
245	CKP_{313}	1	0	4	30	228	1440	12370	116340
246	CKP_{314}	1	0	4	36	36	1800	8500	58800
247	CKP_{315}	1	0	4	36	84	1440	10660	64680
248	CKP_{316}	1	0	4	36	156	1200	12820	90720
249	CKP_{318}	1	0	4	36	324	2160	20740	223440
250	CKP_{319}	1	0	4	42	156	1680	16510	119700
251	CKP_{320}	1	0	4	42	180	2040	19390	155400
252	CKP_{321}	1	0	4	42	252	2040	21190	196980
253		1	0	4	48	180	1920	22000	156240
254	CKP_{322}	1	0	4	60	204	2640	33340	231840
255	CKP_{323}	1	0	4	60	564	4140	49900	648480
256	CKP_{324}, $\mathrm{BØS}^4_{38}$	1	0	6	0	90	120	1860	7560
257	$\mathbb{P}^1 \times \mathrm{MM}^3_{2\text{–}32}$, MW^4_{16}, CKP_{325}	1	0	6	0	114	0	3300	0
258	CKP_{326}, MW^4_8	1	0	6	0	138	0	4740	0
259	MW^4_5, CKP_{327}	1	0	6	0	186	0	7980	0
260	$\mathbb{P}^1 \times \mathbb{P}^1 \times S^2_8$, CKP_{328}, $\mathbb{P}^1 \times \mathrm{MM}^3_{3\text{–}28}$, $\mathrm{BØS}^4_{107}$	1	0	6	6	90	300	1950	13020
261	CKP_{330}, $\mathbb{P}^1 \times \mathrm{MM}^3_{3\text{–}24}$	1	0	6	6	114	300	3390	14280
262	CKP_{332}	1	0	6	6	114	360	3390	18480
263	CKP_{334}	1	0	6	6	138	300	4830	15540
264	CKP_{335}	1	0	6	6	138	360	4830	21000
265		1	0	6	6	138	420	4830	24360
266	CKP_{336}	1	0	6	6	186	360	8070	24780

Continued on next page.

Continued from previous page.

Period ID	Name	α_0	α_1	α_2	α_3	α_4	α_5	α_6	α_7
267	$\mathrm{BØS}^4_{79}$, $S^2_8 \times S^2_7$, CKP_{340}	1	0	6	12	90	540	2760	21420
268	CKP_{341}, $\mathbb{P}^1 \times \mathrm{MM}^3_{2\text{–}29}$	1	0	6	12	90	600	2400	26040
269	$\mathbb{P}^1 \times \mathrm{MM}^3_{4\text{–}10}$, $\mathrm{BØS}^4_{90}$, CKP_{345}	1	0	6	12	114	540	3840	23940
270	CKP_{346}, $\mathbb{P}^1 \times \mathrm{MM}^3_{3\text{–}20}$	1	0	6	12	114	600	3840	28560
271	CKP_{347}	1	0	6	12	114	660	3840	32760
272	CKP_{348}	1	0	6	12	114	660	4200	32760
273	CKP_{349}	1	0	6	12	114	720	4200	36960
274	$\mathbb{P}^1 \times \mathrm{MM}^3_{3\text{–}17}$, CKP_{351}	1	0	6	12	138	600	5280	31080
275	CKP_{352}	1	0	6	12	138	600	5280	33600
276	CKP_{354}	1	0	6	12	138	600	5640	35280
277	CKP_{355}	1	0	6	12	138	660	5280	35280
278	CKP_{356}	1	0	6	12	138	660	5640	36540
279	CKP_{357}	1	0	6	12	138	780	5640	45360
280	CKP_{359}	1	0	6	12	162	600	7080	38640
281	CKP_{360}	1	0	6	12	162	720	7080	44520
282	CKP_{361}	1	0	6	12	186	720	8520	51240
283	CKP_{363}	1	0	6	12	186	900	8880	63000
284	$\mathbb{P}^2 \times S^2_6$, $\mathrm{BØS}^4_{99}$, CKP_{365}	1	0	6	18	90	720	3570	28980
285	CKP_{367}	1	0	6	18	114	780	5010	34860
286	CKP_{368}, $\mathbb{P}^1 \times \mathrm{MM}^3_{3\text{–}18}$	1	0	6	18	114	840	4650	38220
287	CKP_{369}	1	0	6	18	114	960	5010	47040
288		1	0	6	18	114	1140	4650	61740
289	CKP_{372}	1	0	6	18	138	780	6090	39900
290	CKP_{373}, $\mathbb{P}^1 \times \mathrm{MM}^3_{3\text{–}16}$	1	0	6	18	138	900	6090	46620
291	CKP_{374}	1	0	6	18	138	900	6090	47460
292	CKP_{376}	1	0	6	18	138	960	5730	52080
293	CKP_{377}	1	0	6	18	138	960	7170	56700
294		1	0	6	18	138	1020	6450	57960
295		1	0	6	18	138	1080	7890	66780
296	CKP_{378}	1	0	6	18	162	960	7530	58380
297		1	0	6	18	162	960	7890	58380
298	CKP_{380}	1	0	6	18	162	1080	8250	65940
299	CKP_{381}	1	0	6	18	162	1080	8970	71820

Continued on next page.

Continued from previous page.

Period ID	Name	α_0	α_1	α_2	α_3	α_4	α_5	α_6	α_7
300	CKP_{382}	1	0	6	18	186	1080	8970	69720
301	CKP_{383}	1	0	6	18	186	1140	8970	74340
302	CKP_{384}	1	0	6	18	186	1140	9690	76440
303		1	0	6	18	210	1320	11850	96180
304	CKP_{388}, $\mathbb{P}^1 \times \mathrm{MM}^3_{2\text{–}25}$	1	0	6	24	114	1200	5820	57120
305	CKP_{392}	1	0	6	24	138	1080	7980	57960
306	CKP_{393}	1	0	6	24	138	1260	7980	67620
307	CKP_{394}	1	0	6	24	138	1320	9060	78120
308	CKP_{395}	1	0	6	24	138	1440	7980	82320
309	CKP_{397}	1	0	6	24	162	1140	8700	67200
310	CKP_{399}	1	0	6	24	162	1320	9780	80640
311	CKP_{400}	1	0	6	24	186	1200	9780	76440
312	CKP_{401}	1	0	6	24	186	1200	10860	82320
313	$\mathbb{P}^1 \times \mathrm{MM}^3_{2\text{–}24}$, CKP_{402}	1	0	6	24	186	1260	10140	78120
314	CKP_{403}	1	0	6	24	186	1320	10500	85680
315	CKP_{404}	1	0	6	24	186	1560	12660	110880
316	CKP_{405}	1	0	6	24	210	1440	11940	107520
317	CKP_{406}	1	0	6	24	210	1500	12660	107940
318		1	0	6	24	210	1500	12660	110460
319	CKP_{407}	1	0	6	24	210	1620	13020	115500
320		1	0	6	24	210	1800	13380	133980
321		1	0	6	24	210	1800	15180	138600
322		1	0	6	24	234	1800	16620	146160
323	CKP_{408}	1	0	6	24	234	1920	16980	153720
324	CKP_{409}	1	0	6	24	282	1920	19140	169260
325	CKP_{410}	1	0	6	24	282	2280	21300	199080
326		1	0	6	30	162	1680	11670	103320
327		1	0	6	30	186	1800	13470	122220
328	CKP_{411}	1	0	6	30	210	1620	13470	116340
329	CKP_{412}	1	0	6	30	210	1740	14550	126420
330	CKP_{413}	1	0	6	30	234	1680	15270	133560
331	CKP_{414}	1	0	6	30	234	1980	16710	159600
332	CKP_{415}	1	0	6	30	282	1980	18150	168420
333	CKP_{416}	1	0	6	30	282	2160	19950	186480

Continued on next page.

Continued from previous page.

Period ID	Name	α_0	α_1	α_2	α_3	α_4	α_5	α_6	α_7
334	CKP_{417}	1	0	6	36	186	1560	12480	97440
335	CKP_{418}	1	0	6	36	186	1920	15360	131880
336	CKP_{419}	1	0	6	36	186	2040	15720	138600
337	CKP_{420}	1	0	6	36	186	2520	16080	180600
338	CKP_{421}	1	0	6	36	210	1800	16440	136920
339		1	0	6	36	210	2100	16800	154980
340	CKP_{422}	1	0	6	36	234	1800	16080	137760
341		1	0	6	36	234	2520	19680	201600
342		1	0	6	36	258	2280	20400	191520
343		1	0	6	36	258	2340	21120	196980
344		1	0	6	36	282	2520	22920	224280
345	CKP_{423}	1	0	6	36	306	2280	23280	221760
346		1	0	6	36	330	2640	27600	274680
347	CKP_{424}	1	0	6	36	330	2880	27600	278040
348	CKP_{425}	1	0	6	36	330	3240	30480	312480
349	CKP_{426}	1	0	6	36	378	3480	34080	352800
350		1	0	6	42	162	2760	17610	178920
351	CKP_{427}	1	0	6	42	306	2460	24090	229320
352	CKP_{428}	1	0	6	42	306	2820	26970	270060
353	CKP_{429}	1	0	6	48	282	2760	27420	253680
354	CKP_{430}	1	0	6	48	282	2760	28500	257040
355	CKP_{432}	1	0	6	48	426	3360	37860	406560
356	CKP_{433}	1	0	6	48	522	4800	51180	595560
357	CKP_{435}	1	0	6	54	378	3480	38670	392700
358	CKP_{436}	1	0	6	60	354	4080	44520	441840
359	CKP_{437}	1	0	6	60	474	3960	45600	503160
360	CKP_{438}	1	0	6	66	474	4860	57930	637560
361		1	0	6	84	714	6840	96360	1211280
362	CKP_{439}	1	0	6	120	1146	11280	192300	2817360
363	MW^4_{18}, $\mathrm{BØS}^4_{119}$, CKP_{440}, $\mathbb{P}^1\times\mathbb{P}^1\times\mathbb{P}^1\times\mathbb{P}^1$, $\mathbb{P}^1\times\mathrm{MM}^3_{3\text{–}27}$	1	0	8	0	168	0	5120	0
364	MW^4_9, $\mathbb{P}^1 \times B^3_5$	1	0	8	0	192	0	6920	0
365	V^4_{14}	1	0	8	0	288	0	15200	0

Continued on next page.

Continued from previous page.

Period ID	Name	α_0	α_1	α_2	α_3	α_4	α_5	α_6	α_7
366	$\mathbb{P}^1 \times \mathrm{MM}^3_{4\text{–}11}$, $\mathrm{BØS}^4_{97}$, CKP_{442}, $\mathbb{P}^1 \times \mathbb{P}^1 \times S^2_7$	1	0	8	6	168	360	5210	19740
367	$\mathbb{P}^1 \times \mathrm{MM}^3_{3\text{–}21}$, CKP_{443}	1	0	8	6	192	360	7010	21000
368	CKP_{444}	1	0	8	6	216	360	8810	22260
369	$\mathbb{P}^1 \times \mathrm{MM}^3_{4\text{–}9}$, CKP_{445}	1	0	8	12	168	720	5660	39480
370	CKP_{446}, $S^2_7 \times S^2_7$, $\mathrm{BØS}^4_{75}$	1	0	8	12	168	720	6020	39480
371	CKP_{447}, $\mathbb{P}^1 \times \mathrm{MM}^3_{4\text{–}8}$	1	0	8	12	192	720	7460	42000
372	$\mathbb{P}^1 \times \mathrm{MM}^3_{2\text{–}26}$	1	0	8	12	192	780	7460	47880
373	CKP_{448}	1	0	8	12	216	840	9620	57960
374		1	0	8	12	216	1440	8540	126000
375	CKP_{449}	1	0	8	12	288	1080	16100	92400
376	CKP_{450}	1	0	8	12	360	1200	23300	117600
377	$S^2_8 \times S^2_6$, $\mathrm{BØS}^4_{78}$, CKP_{451}	1	0	8	18	168	1020	6830	54600
378	CKP_{452}	1	0	8	18	168	1080	6470	59220
379	CKP_{454}, $\mathbb{P}^1 \times \mathrm{MM}^3_{4\text{–}7}$	1	0	8	18	192	1080	8270	63000
380	CKP_{455}	1	0	8	18	216	1080	10070	69300
381	CKP_{456}, $\mathbb{P}^1 \times \mathrm{MM}^3_{3\text{–}15}$	1	0	8	18	216	1140	10070	72660
382	CKP_{457}	1	0	8	18	216	1200	10430	79380
383	CKP_{458}	1	0	8	18	216	1260	11150	87360
384	CKP_{459}	1	0	8	18	240	1380	13310	105000
385		1	0	8	18	288	1560	16910	136500
386	CKP_{460}	1	0	8	24	168	1440	8360	78960
387	$\mathbb{P}^1 \times \mathrm{MM}^3_{4\text{–}5}$, CKP_{461}	1	0	8	24	216	1440	10880	89040
388	$\mathbb{P}^1 \times \mathrm{MM}^3_{2\text{–}22}$	1	0	8	24	216	1560	11240	100800
389	CKP_{462}	1	0	8	24	216	2160	11240	168000
390	$\mathbb{P}^1 \times \mathrm{MM}^3_{3\text{–}13}$, CKP_{463}	1	0	8	24	240	1560	13040	105840
391	CKP_{464}	1	0	8	24	240	1560	13400	110460
392	CKP_{465}	1	0	8	24	240	1740	13760	126420
393	CKP_{466}	1	0	8	24	264	1680	15200	126840
394	CKP_{467}	1	0	8	24	264	1740	15920	133980
395	CKP_{468}	1	0	8	24	264	1920	17360	154560
396	CKP_{469}	1	0	8	24	288	1920	18440	159600
397	CKP_{470}	1	0	8	24	312	2160	22040	194880
398		1	0	8	24	360	2160	25640	218400

Continued on next page.

Continued from previous page.

Period ID	Name	α_0	α_1	α_2	α_3	α_4	α_5	α_6	α_7
399	CKP_{471}	1	0	8	30	216	1800	13490	116340
400	$\mathbb{P}^1 \times MM^3_{3\text{–}11}$, CKP_{472}	1	0	8	30	264	1980	16370	142800
401	CKP_{473}	1	0	8	30	264	1980	16730	147000
402	CKP_{474}	1	0	8	30	264	2160	17090	165900
403		1	0	8	30	288	2100	19250	171360
404		1	0	8	30	288	2220	20330	185640
405	CKP_{475}	1	0	8	30	312	2580	23930	229320
406		1	0	8	30	336	2520	25010	233940
407		1	0	8	36	216	3600	16100	294000
408	CKP_{476}	1	0	8	36	264	2280	19340	173880
409	CKP_{477}	1	0	8	36	264	2880	20420	232680
410	CKP_{478}	1	0	8	36	288	2700	21500	216720
411	CKP_{479}	1	0	8	36	312	2520	22940	210420
412	CKP_{480}	1	0	8	36	312	2760	24380	239820
413		1	0	8	36	312	2760	25100	243600
414	CKP_{481}	1	0	8	36	336	2760	26180	251160
415	CKP_{482}	1	0	8	36	360	2760	27260	261240
416	CKP_{483}	1	0	8	36	360	2940	28340	277200
417	CKP_{484}	1	0	8	36	360	3060	29780	295680
418	CKP_{485}	1	0	8	36	360	3300	31220	320040
419	CKP_{486}	1	0	8	36	408	3360	35180	358680
420	CKP_{487}	1	0	8	36	432	3780	39500	413280
421		1	0	8	42	312	3000	27350	263340
422		1	0	8	42	360	3540	32750	346080
423		1	0	8	42	408	3480	36350	368340
424		1	0	8	42	456	4320	44270	479220
425	CKP_{488}	1	0	8	48	264	4320	27440	366240
426		1	0	8	48	336	4680	35000	467040
427	CKP_{489}, $\mathbb{P}^1 \times MM^3_{2\text{–}18}$	1	0	8	48	360	3360	31040	295680
428		1	0	8	48	384	3960	37160	400680
429	CKP_{490}	1	0	8	48	408	3960	38960	410760
430		1	0	8	48	432	4140	42560	454440
431		1	0	8	48	432	4320	44720	487200
432	CKP_{491}	1	0	8	48	504	4800	51560	572040

Continued on next page.

Continued from previous page.

Period ID	Name	α_0	α_1	α_2	α_3	α_4	α_5	α_6	α_7
433	CKP_{492}	1	0	8	48	504	4920	53000	613200
434	CKP_{493}	1	0	8	54	360	4200	39770	406980
435	CKP_{494}	1	0	8	54	480	5160	53810	608580
436	CKP_{495}	1	0	8	60	360	5160	45260	514080
437	CKP_{496}	1	0	8	60	552	5280	60740	685440
438		1	0	8	60	672	7200	83060	1032360
439		1	0	8	66	456	6000	61550	699300
440	CKP_{497}	1	0	8	72	792	8460	104120	1339800
441	CKP_{498}	1	0	8	84	408	8040	78740	887040
442	$\mathbb{P}^1 \times B^3_4$, CKP_{500}, MW^4_6	1	0	10	0	318	0	15220	0
443	V^4_{12}	1	0	10	0	438	0	28900	0
444	$\mathbb{P}^1 \times \mathrm{MM}^3_{5\text{–}3}$, $\mathrm{BØS}^4_{98}$, CKP_{501}, $\mathbb{P}^1 \times \mathbb{P}^1 \times S^2_6$	1	0	10	12	270	840	11080	55440
445	$\mathbb{P}^1 \times \mathrm{MM}^3_{2\text{–}23}$, CKP_{502}	1	0	10	12	318	960	15760	74760
446	CKP_{503}	1	0	10	12	366	960	20800	82320
447	CKP_{504}, $S^2_7 \times S^2_6$, $\mathrm{BØS}^4_{76}$	1	0	10	18	270	1320	12610	91560
448	CKP_{505}, $\mathbb{P}^1 \times \mathrm{MM}^3_{4\text{–}4}$	1	0	10	24	318	1800	17380	135240
449	CKP_{506}	1	0	10	24	318	2400	18460	215040
450	$\mathbb{P}^1 \times \mathrm{MM}^3_{2\text{–}21}$	1	0	10	24	342	1920	19900	154560
451	CKP_{507}	1	0	10	24	462	2640	35740	287280
452	$\mathbb{P}^1 \times \mathrm{MM}^3_{3\text{–}12}$, CKP_{508}	1	0	10	30	342	2340	21070	186060
453	CKP_{509}, $\mathbb{P}^1 \times \mathrm{MM}^3_{2\text{–}19}$	1	0	10	30	342	2520	21430	208740
454	CKP_{510}	1	0	10	30	366	2520	24670	221760
455	CKP_{511}	1	0	10	30	462	2760	35110	290640
456	CKP_{512}, $\mathbb{P}^2 \times S^2_5$	1	0	10	36	270	2160	15040	134400
457	CKP_{513}	1	0	10	36	366	2760	25840	235200
458	CKP_{514}	1	0	10	36	366	2880	28000	271740
459	CKP_{515}	1	0	10	36	366	3000	26200	260400
460	$\mathbb{P}^1 \times \mathrm{MM}^3_{2\text{–}20}$	1	0	10	36	390	2940	27640	255360
461	CKP_{516}	1	0	10	36	390	3000	28720	273000
462	CKP_{517}	1	0	10	36	414	3180	31960	306600
463	CKP_{518}	1	0	10	36	414	3480	33400	351960
464		1	0	10	36	486	3720	42400	420000
465	CKP_{520}	1	0	10	42	414	3480	33850	334320

Continued on next page.

Continued from previous page.

Period ID	Name	α_0	α_1	α_2	α_3	α_4	α_5	α_6	α_7
466	CKP_{521}	1	0	10	42	414	3840	38530	407820
467	CKP_{522}	1	0	10	42	462	4080	41770	436800
468	CKP_{523}	1	0	10	48	414	4080	36460	387240
469	CKP_{524}	1	0	10	48	414	4320	38260	425040
470	CKP_{525}	1	0	10	48	462	4200	41140	425880
471	CKP_{526}	1	0	10	48	486	4320	44740	465360
472		1	0	10	48	486	4680	47260	519960
473		1	0	10	48	486	5400	49420	631680
474		1	0	10	48	510	5160	56260	632100
475	CKP_{527}	1	0	10	48	534	4920	53020	578760
476	CKP_{528}	1	0	10	48	558	5280	57700	646800
477	CKP_{529}	1	0	10	54	486	4920	49150	534240
478		1	0	10	54	534	5100	54550	594300
479	CKP_{530}	1	0	10	54	582	5580	63910	711060
480	CKP_{531}	1	0	10	54	606	6180	68230	805140
481	CKP_{532}	1	0	10	60	510	6120	59680	714000
482		1	0	10	60	582	5760	61480	683760
483		1	0	10	60	582	6360	68680	807240
484	CKP_{533}	1	0	10	60	654	6840	77680	924840
485	CKP_{534}	1	0	10	60	654	7080	77320	945840
486	CKP_{535}	1	0	10	66	750	7920	93970	1156680
487	CKP_{536}	1	0	10	66	846	10080	125290	1619940
488	CKP_{538}	1	0	10	72	558	7200	74620	890400
489	CKP_{539}	1	0	10	72	726	8280	97660	1212120
490	CKP_{540}	1	0	10	72	846	9360	113860	1475880
491	CKP_{541}	1	0	10	78	750	8700	107110	1328880
492	CKP_{542}	1	0	10	78	846	10140	124030	1643880
493	CKP_{543}	1	0	10	84	750	8520	102880	1244040
494	CKP_{544}	1	0	10	96	702	10560	124660	1538880
495	CKP_{545}	1	0	10	168	1566	23040	402940	6002640
496	V^4_{10}	1	0	12	0	684	0	58800	0
497	$S^2_6 \times S^2_6$, CKP_{546}, $BØS^4_{77}$	1	0	12	24	396	2160	23160	186480
498	$\mathbb{P}^1 \times MM^3_{4-3}$, CKP_{547}	1	0	12	24	444	2160	26760	191520
499	$S^2_8 \times S^2_5$, CKP_{548}	1	0	12	36	396	2820	24060	219240

Continued on next page.

Continued from previous page.

Period ID	Name	α_0	α_1	α_2	α_3	α_4	α_5	α_6	α_7
500	$\mathbb{P}^1 \times \mathrm{MM}^3_{3\text{–}10}$, CKP_{549}	1	0	12	36	492	3360	35220	319200
501	CKP_{550}	1	0	12	36	492	3540	38460	371700
502	CKP_{551}	1	0	12	36	540	5400	41700	705600
503	$\mathbb{P}^1 \times \mathrm{MM}^3_{2\text{–}17}$	1	0	12	42	540	4140	43230	423360
504	CKP_{552}	1	0	12	42	540	4560	49710	528360
505	CKP_{553}, $\mathbb{P}^1 \times \mathrm{MM}^3_{3\text{–}7}$	1	0	12	48	564	4680	48000	486360
506	CKP_{554}	1	0	12	48	588	5040	54480	577920
507	CKP_{555}	1	0	12	48	588	5040	55200	588000
508		1	0	12	48	636	5940	68880	780780
509		1	0	12	54	732	7680	84810	1136520
510	CKP_{556}	1	0	12	60	636	6000	64020	698460
511	CKP_{557}, $\mathbb{P}^1 \times \mathrm{MM}^3_{2\text{–}16}$	1	0	12	60	636	6120	63300	693000
512		1	0	12	60	684	6840	76620	893760
513	CKP_{558}	1	0	12	60	684	6840	77340	898800
514	CKP_{559}	1	0	12	60	708	6840	77700	893760
515	CKP_{560}	1	0	12	60	780	8400	101460	1254960
516		1	0	12	66	804	8400	100830	1237740
517		1	0	12	66	828	8880	108030	1369620
518	CKP_{561}	1	0	12	72	708	9120	93000	1254960
519		1	0	12	72	756	8580	97320	1209180
520	CKP_{562}	1	0	12	72	780	8340	97320	1178520
521		1	0	12	72	828	9000	108480	1354920
522	CKP_{563}	1	0	12	72	876	9600	118200	1501920
523	CKP_{564}	1	0	12	78	876	10440	125490	1649340
524		1	0	12	84	876	9960	122700	1540560
525	CKP_{565}	1	0	12	90	1116	13860	184350	2553600
526		1	0	12	96	1140	14400	193080	2721600
527		1	0	12	96	1356	17640	245640	3609480
528	CKP_{566}	1	0	12	108	756	16320	155100	2494800
529		1	0	12	120	1284	17700	253200	3671640
530	$\mathbb{P}^1 \times B^3_3$, MW^4_3, CKP_{567}	1	0	14	0	690	0	50900	0
531	$\mathbb{P}^1 \times \mathbb{P}^1 \times S^2_5$, $\mathbb{P}^1 \times \mathrm{MM}^3_{6\text{–}1}$, CKP_{568}	1	0	14	30	546	2760	33350	246540
532	$S^2_7 \times S^2_5$, CKP_{569}	1	0	14	36	546	3480	37040	330540

Continued on next page.

Continued from previous page.

Period ID	Name	α_0	α_1	α_2	α_3	α_4	α_5	α_6	α_7
533	CKP_{570}	1	0	14	36	690	3960	57200	468720
534	CKP_{571}	1	0	14	36	690	5760	59000	821520
535	CKP_{572}, $\mathbb{P}^1 \times MM^3_{2-15}$	1	0	14	36	714	4320	59720	519120
536	CKP_{573}	1	0	14	36	858	4560	83840	637560
537	CKP_{574}, $\mathbb{P}^1 \times MM^3_{4-1}$	1	0	14	48	690	5280	59540	594720
538	$\mathbb{P}^1 \times MM^3_{3-8}$, CKP_{575}	1	0	14	54	690	5700	61070	631260
539	CKP_{576}	1	0	14	60	786	7140	82760	933240
540	CKP_{577}	1	0	14	60	786	7320	84920	981120
541	CKP_{578}	1	0	14	66	834	8160	95450	1126440
542	CKP_{579}	1	0	14	72	882	9240	109940	1355760
543	CKP_{580}	1	0	14	72	1002	10800	138020	1807680
544	CKP_{581}	1	0	14	72	1026	10560	136220	1733760
545	CKP_{582}	1	0	14	78	834	8880	98870	1177260
546	CKP_{583}	1	0	14	78	906	9600	112190	1363320
547		1	0	14	78	1146	12780	174830	2377620
548	CKP_{584}	1	0	14	84	930	10320	122720	1529640
549		1	0	14	84	1074	12600	161600	2187360
550	CKP_{585}	1	0	14	84	1074	12720	163040	2202060
551	CKP_{586}	1	0	14	96	1170	14040	184820	2526720
552		1	0	14	96	1194	15360	201740	2897160
553	CKP_{587}	1	0	14	96	1266	15240	207860	2918160
554		1	0	14	96	1434	18600	276620	4253760
555		1	0	14	102	1242	15720	211910	2994600
556	CKP_{588}	1	0	14	102	1338	17280	237830	3452400
557	CKP_{589}	1	0	14	102	1530	19800	284990	4270980
558		1	0	14	108	1218	17400	224600	3334800
559		1	0	14	108	1314	18240	245120	3690960
560	CKP_{590}	1	0	14	120	1506	20640	296420	4484760
561	CKP_{591}	1	0	14	120	1554	20520	298940	4515000
562		1	0	14	138	2106	30120	474530	7913220
563	CKP_{592}	1	0	14	144	1506	21480	311900	4544400
564	CKP_{593}	1	0	14	144	1506	24480	349700	5456640
565	CKP_{594}	1	0	14	156	2226	33000	534200	9067800
566		1	0	14	180	2082	33480	560480	9276960

Continued on next page.

Continued from previous page.

Period ID	Name	α_0	α_1	α_2	α_3	α_4	α_5	α_6	α_7
567	CKP_{595}	1	0	14	288	2994	58440	1220900	21414960
568	CKP_{596}, V_8^4	1	0	16	0	1296	0	160000	0
569	CKP_{597}	1	0	16	24	1296	4320	163240	840000
570	$S_6^2 \times S_5^2$, CKP_{598}	1	0	16	42	720	4920	58390	567840
571	CKP_{599}	1	0	16	60	1344	11520	192940	2347800
572	CKP_{600}, $\mathbb{P}^1 \times \mathrm{MM}^3_{3\text{–}6}$	1	0	16	66	936	8280	97630	1086540
573	$\mathbb{P}^1 \times \mathrm{MM}^3_{2\text{–}12}$, CKP_{601}	1	0	16	72	1056	9840	122920	1428000
574	CKP_{602}	1	0	16	78	1080	11040	138490	1725780
575	CKP_{603}, $\mathbb{P}^1 \times \mathrm{MM}^3_{2\text{–}13}$	1	0	16	84	1104	11400	137860	1685040
576	CKP_{604}	1	0	16	84	1152	12600	162700	2132760
577	CKP_{605}	1	0	16	90	1176	12900	164590	2139060
578	CKP_{606}	1	0	16	90	1200	13440	175750	2332680
579		1	0	16	96	1632	19320	302200	4447800
580	$\mathbb{P}^1 \times \mathrm{MM}^3_{2\text{–}11}$, CKP_{607}	1	0	16	108	1248	15600	188260	2538480
581	CKP_{608}	1	0	16	108	1488	18600	261700	3797640
582		1	0	16	108	1488	18960	267460	3922800
583		1	0	16	108	1632	20640	309220	4640160
584	CKP_{609}	1	0	16	114	1488	19440	268990	3973620
585	CKP_{610}	1	0	16	114	1488	19740	275470	4077780
586	CKP_{611}	1	0	16	114	1512	23640	303190	5285700
587	CKP_{612}	1	0	16	120	1488	19440	268360	3894240
588	CKP_{613}	1	0	16	126	1752	23940	355570	5509980
589		1	0	16	204	3264	52680	952180	18086880
590	CKP_{614}	1	0	18	48	1494	9120	206820	1864800
591	$\mathbb{P}^1 \times \mathrm{MM}^3_{2\text{–}14}$	1	0	18	90	1302	13260	168570	2089080
592	CKP_{615}	1	0	18	102	1398	16200	212670	2919420
593		1	0	18	108	1542	18180	249480	3486420
594	CKP_{616}	1	0	18	114	1542	19200	262890	3780420
595	CKP_{617}	1	0	18	120	1878	23400	351180	5323080
596	CKP_{618}	1	0	18	120	1878	25200	379980	6032880
597		1	0	18	120	2022	26160	421020	6607440
598	CKP_{619}	1	0	18	132	1926	25800	388800	6041280
599	CKP_{620}	1	0	18	138	2166	30240	478170	7777560

Continued on next page.

Continued from previous page.

Period ID	Name	α_0	α_1	α_2	α_3	α_4	α_5	α_6	α_7
600		1	0	18	144	2118	30960	481860	7971600
601	CKP_{621}	1	0	18	156	2190	32760	513720	8536080
602	CKP_{622}	1	0	18	156	2310	33240	537120	8919960
603		1	0	18	156	2358	34920	564120	9502920
604		1	0	18	174	2454	38880	636030	11007780
605	CKP_{623}	1	0	18	192	2862	46440	802980	14515200
606	CKP_{624}	1	0	18	228	2934	55320	969840	18061680
607	CKP_{625}, $S^2_5 \times S^2_5$	1	0	20	60	1140	9120	121700	1377600
608	CKP_{626}, $\mathbb{P}^2 \times S^2_4$	1	0	20	102	1188	11760	123050	1391880
609	CKP_{627}	1	0	20	120	1668	21120	303320	4519200
610	CKP_{628}	1	0	20	120	1860	23280	342200	5115600
611	CKP_{629}	1	0	20	126	1908	24480	361010	5470920
612		1	0	20	144	2148	31800	505280	8329440
613	CKP_{630}	1	0	20	156	2340	34080	540740	8942640
614	Str_3	1	0	20	156	2700	41040	697700	12503400
615	CKP_{631}	1	0	20	168	2580	38400	629120	10709160
616		1	0	20	168	2580	39600	648920	11239200
617		1	0	20	198	3228	52260	925130	17075100
618	CKP_{633}, $S^2_8 \times S^2_4$	1	0	22	102	1434	13740	160510	1881180
619	CKP_{634}	1	0	22	120	1914	23280	347980	5206320
620	$\mathbb{P}^1 \times \mathrm{MM}^3_{3\text{–}3}$, CKP_{635}	1	0	22	132	2058	24360	345280	4867800
621	CKP_{636}	1	0	22	144	2394	34200	557140	9241680
622	CKP_{637}	1	0	22	162	2490	34260	531490	8504160
623		1	0	22	168	2634	38040	613660	10263120
624	CKP_{638}	1	0	22	186	3090	47880	824530	14728980
625		1	0	22	186	3354	52980	960970	17852100
626		1	0	22	192	3258	51720	914620	16742880
627	CKP_{639}	1	0	22	246	4290	74280	1433830	28650720
628		1	0	22	264	4122	77880	1476220	29789760
629		1	0	22	264	4554	82200	1613740	33027120
630	CKP_{640}, V^4_6	1	0	24	0	3240	0	672000	0
631	CKP_{641}	1	0	24	36	3240	10800	680100	3528000
632	CKP_{642}	1	0	24	72	3288	21600	720600	7101360

Continued on next page.

Continued from previous page.

Period ID	Name	α_0	α_1	α_2	α_3	α_4	α_5	α_6	α_7
633	$\mathbb{P}^1 \times MM^3_{7-1}$, $\mathbb{P}^1 \times \mathbb{P}^1 \times S^2_4$, CKP_{643}	1	0	24	96	1704	14400	193920	2150400
634	$S^2_7 \times S^2_4$, CKP_{644}	1	0	24	102	1704	15720	205530	2452380
635	CKP_{645}	1	0	24	144	3480	46920	909600	16450560
636	CKP_{646}, $\mathbb{P}^1 \times MM^3_{2-9}$	1	0	24	174	2784	37680	578490	9059820
637		1	0	24	186	3144	47280	804390	14118720
638	CKP_{647}	1	0	24	192	3048	45840	757680	13077120
639	CKP_{648}	1	0	24	192	3192	48120	816000	14306040
640	CKP_{649}	1	0	24	234	3648	60780	1060350	19603500
641	CKP_{650}	1	0	24	264	4632	83040	1611960	32664240
642		1	0	24	264	5352	101040	2040360	43219680
643	CKP_{651}	1	0	26	72	3534	22320	787580	7514640
644	CKP_{652}, $S^2_6 \times S^2_4$	1	0	26	108	1998	19080	270440	3435600
645	CKP_{653}	1	0	26	216	4302	72480	1371500	27676320
646	CKP_{654}	1	0	26	246	4302	72120	1339550	25814460
647	CKP_{655}	1	0	26	288	5166	102960	2038580	44530080
648	CKP_{656}	1	0	26	396	6222	151080	3168440	74446680
649	CKP_{657}	1	0	28	240	3996	62400	1067680	19007520
650	CKP_{658}	1	0	28	258	4764	82200	1573390	31316460
651	CKP_{659}	1	0	28	288	5484	100800	2038960	42887040
652	CKP_{660}	1	0	28	306	5580	104100	2099350	44273880
653		1	0	28	342	6540	129540	2770570	61901700
654	CKP_{661}	1	0	28	432	9660	210240	5004640	126134400
655	$S^2_5 \times S^2_4$, CKP_{662}	1	0	30	126	2658	27720	439590	6247500
656	$\mathbb{P}^1 \times MM^3_{2-10}$, CKP_{663}	1	0	30	216	3858	54000	891660	14726880
657	CKP_{664}	1	0	30	240	4338	66960	1182900	21408240
658	CKP_{665}	1	0	30	300	6690	124920	2778600	61790400
659	CKP_{666}	1	0	30	372	7314	153720	3385200	79195200
660	CKP_{667}	1	0	32	318	6144	113280	2304770	48799800
661	CKP_{668}	1	0	32	384	7728	157800	3492320	80806320
662		1	0	32	384	8112	167520	3766640	88438560
663	$\mathbb{P}^1 \times V^3_{14}$	1	0	34	312	5910	97920	1820140	34520640
664		1	0	34	390	8694	179520	4180750	100127580
665		1	0	34	498	10278	245040	5923330	153543600

Continued on next page.

Continued from previous page.

Period ID	Name	α_0	α_1	α_2	α_3	α_4	α_5	α_6	α_7
666	CKP_{669}	1	0	36	336	6708	119520	2419200	50507520
667	CKP_{670}	1	0	36	360	7188	134400	2795400	60459840
668		1	0	36	396	7572	143160	2921580	62324640
669		1	0	36	456	9876	214680	5072760	125137740
670	CKP_{671}	1	0	36	552	12852	304080	7828200	210966000
671		1	0	36	768	18996	500640	14713200	450203040
672	CKP_{672}, $\mathbb{P}^1 \times \mathrm{MM}^3_{2\text{–}7}$	1	0	38	348	6954	117840	2268560	44336040
673	CKP_{673}	1	0	38	384	8106	156480	3390500	76130880
674	CKP_{674}	1	0	38	396	8010	150600	3136160	67735080
675	CKP_{675}, $S^2_4 \times S^2_4$	1	0	40	192	4776	59520	1120000	19138560
676	CKP_{676}	1	0	44	516	11580	248880	5903540	145945800
677	CKP_{677}	1	0	44	636	15804	393480	10666340	301939680
678	CKP_{678}	1	0	44	696	17388	445680	12371480	359059680
679		1	0	44	744	18396	492360	14028200	419215440
680	CKP_{679}	1	0	44	888	23052	649200	19904120	635293680
681	$\mathbb{P}^1 \times \mathrm{MM}^3_{2\text{–}6}$, CKP_{680}	1	0	46	528	11826	238560	5341780	122340960
682	CKP_{681}	1	0	46	714	18618	496560	14203810	428469300
683	V^4_4, CKP_{682}	1	0	48	0	15120	0	7392000	0
684	CKP_{683}	1	0	48	216	15408	151320	7959000	117482400
685	CKP_{684}	1	0	48	660	15552	367320	9396300	251895000
686	$\mathbb{P}^1 \times V^3_{12}$	1	0	50	600	13758	288480	6659420	157802400
687	CKP_{685}	1	0	50	792	21078	635760	18069260	600739440
688	CKP_{686}	1	0	52	696	17412	424440	11365000	317604000
689		1	0	52	1044	29124	874080	28285540	956113200
690	$\mathbb{P}^2 \times S^2_3$, CKP_{687}	1	0	54	498	9882	162000	2938770	54057780
691	CKP_{688}	1	0	54	528	11178	207720	4427820	98491680
692	CKP_{689}	1	0	54	744	19194	481680	13279500	381906000
693	CKP_{690}	1	0	54	888	24378	677520	20447820	644873040
694	CKP_{692}, $S^2_8 \times S^2_3$	1	0	56	498	10536	171900	3240110	60897480
695	CKP_{693}	1	0	56	528	11832	217920	4748600	106293600
696	CKP_{694}	1	0	56	600	14424	317100	7961600	207233040
697	$\mathbb{P}^1 \times \mathbb{P}^1 \times S^2_3$, CKP_{695}, $\mathbb{P}^1 \times \mathrm{MM}^3_{8\text{–}1}$	1	0	58	492	11214	178440	3502120	65938320
698	CKP_{696}, $S^2_7 \times S^2_3$	1	0	58	498	11214	181800	3561250	68151720

Continued on next page.

Continued from previous page.

Period ID	Name	α_0	α_1	α_2	α_3	α_4	α_5	α_6	α_7
699		1	0	58	888	23694	632400	18393340	559525680
700	$S^2_6 \times S^2_3$, CKP_{697}	1	0	60	504	11916	195120	3962040	78104880
701	CKP_{698}	1	0	60	1068	30156	893280	28423860	948659040
702	CKP_{699}	1	0	60	1212	35916	1134480	38512860	1368087000
703	CKP_{700}, $S^2_5 \times S^2_3$	1	0	64	522	13392	225720	4887190	102194400
704	CKP_{701}	1	0	66	852	21510	504000	13009080	347891040
705	CKP_{702}	1	0	66	1356	47574	1614240	58420920	2223985680
706	$\mathbb{P}^1 \times \mathrm{MM}^3_{2-5}$, CKP_{703}	1	0	68	816	21012	465960	11662880	297392760
707	CKP_{704}	1	0	68	852	22308	520680	13640900	368091360
708	CKP_{705}	1	0	68	1320	43236	1421040	51100520	1914785040
709	$S^2_4 \times S^2_3$, CKP_{706}	1	0	74	588	17550	319560	7862600	185440080
710	CKP_{707}	1	0	78	1140	32706	877320	26208960	814453920
711	CKP_{708}	1	0	78	1176	34002	937080	28577940	909170640
712	CKP_{709}	1	0	78	1680	60066	2142720	82424580	3324124440
713	CKP_{710}	1	0	80	1212	36240	1020360	31974020	1043489160
714	$\mathbb{P}^1 \times V^3_{10}$	1	0	80	1320	38688	1078320	32604200	1016215200
715		1	0	84	1932	69636	2622480	106446900	4526098920
716	CKP_{711}	1	0	84	2148	77316	3051480	128188740	5649930720
717		1	0	90	1788	59886	2032920	74950920	2894154480
718	CKP_{712}	1	0	90	2040	76014	2873160	117404820	5023514160
719	$\mathbb{P}^1 \times \mathrm{MM}^3_{2-4}$, CKP_{713}	1	0	92	1518	47172	1357680	42774050	1385508600
720	CKP_{714}	1	0	92	1626	51492	1574580	52448150	1816414320
721	CKP_{715}	1	0	92	2112	83820	3281280	141863600	6368328960
722	CKP_{716}	1	0	102	1950	67002	2266320	83881470	3245543280
723		1	0	102	2274	84330	3207480	132223890	5710371660
724	CKP_{717}	1	0	102	2688	106410	4495680	203447460	9658434240
725	CKP_{718}	1	0	102	3408	146250	6695280	334814340	17506424880
726	CKP_{719}	1	0	104	2472	97944	3940320	171825080	7840793520
727	CKP_{720}, $S^2_3 \times S^2_3$	1	0	108	984	37260	848880	26609400	804368880
728	CKP_{721}	1	0	128	2976	120960	4959840	221633120	10369947840
729	CKP_{722}	1	0	138	4650	222918	11448480	632940330	36647730000
730		1	0	150	4866	241002	12623040	711272850	42024975300
731	CKP_{723}, $\mathbb{P}^1 \times V^3_8$	1	0	154	3840	159486	6504960	284808340	12889551360
732	CKP_{724}	1	0	168	4752	219624	10383840	531501360	28511659680

Continued on next page.

Continued from previous page.

Period ID	Name	α_0	α_1	α_2	α_3	α_4	α_5	α_6	α_7
733	CKP_{725}	1	0	184	5688	286008	14876160	837897160	49505030400
734		1	0	224	9312	580704	38555520	2752140320	206084027520
735	CKP_{726}	1	0	272	13560	952176	73148160	5996559080	516454715280
736	CKP_{727}, $\mathbb{P}^1 \times V_6^3$	1	0	398	17616	1221810	85572960	6386359700	493612489440
737	CKP_{728}	1	0	420	19992	1488708	114603120	9497959800	824518956240
738	CKP_{729}	1	0	444	22404	1771596	146305440	13047797460	1221757064640
739	CKP_{730}	1	0	468	24852	2065764	180367920	17014559940	1685867765400
740		1	0	540	37632	3836268	420664320	49565795760	6131551910400
741	CKP_{731}	1	0	1040	105984	15564048	2472668160	422070022400	75673543680000
742		1	0	1386	166284	28575342	5322513240	1065056580360	223880895211680
743	$\mathbb{P}^1 \times V_4^3$, CKP_{732}	1	0	1946	215808	35318526	5981882880	1074550170260	200205416839680
744	CKP_{733}	1	0	1992	227472	38459880	6796332000	1282447706160	252711084477600
745	CKP_{734}	1	0	2136	262896	48275736	9412519800	1975803279600	435882277192320
746	CKP_{735}	1	0	2664	466368	115475112	31137505920	9021039724800	2746619333498880
747	CKP_{736}	1	0	6804	2040912	852143652	389608626240	191430924575040	98894833331535360
748	CKP_{737}	1	0	12816	5435904	3188239632	2051802731520	1419118168838400	1032164932439531520
749	CKP_{738}	1	0	99000	130800000	233995275000	462392774925120	982577026659240000	2197113382189414080000

References

[1] Code repository. bitbucket.org/fanosearch/magma-core, 2018.

[2] Code and data repository. bitbucket.org/fanosearch/db, 2018.

[3] W. Bosma, J. Cannon, and C. Playoust. The Magma algebra system. I. The user language. *J. Symbolic Comput.*, 24(3-4):235–265, 1997. Computational algebra and number theory (London, 1993).

[4] T. Coates, A. Corti, S. Galkin, and A. Kasprzyk. Quantum periods for 3-dimensional Fano manifolds. *Geom. Topol.*, 20(1):103–256, 2016.

[5] T. Coates, S. Galkin, A. Kasprzyk, and A. Strangeway. Quantum periods for certain four-dimensional Fano manifolds. *Experimental Mathematics*, 0(0):1–39, 2018.

[6] T. Coates, A. Kasprzyk, and T. Prince. Four-dimensional Fano toric complete intersections. *Proc. A.*, 471(2175):20140704, 14, 2015.

[7] S. Martin. Symplectic quotients by a nonabelian group and by its maximal torus. arXiv:math/0001002 [math.SG], 2000.

[8] S. Mori and S. Mukai. Classification of Fano 3-folds with $B_2 \geq 2$. *Manuscripta Math.*, 36(2):147–162, 1981/82.

[9] S. Mori and S. Mukai. On Fano 3-folds with $B_2 \geq 2$. In *Algebraic varieties and analytic varieties (Tokyo, 1981)*, volume 1 of *Adv. Stud. Pure Math.*, pages 101–129. North-Holland, Amsterdam, 1983.

[10] S. Mori and S. Mukai. Classification of Fano 3-folds with $B_2 \geq 2$. I. In *Algebraic and topological theories (Kinosaki, 1984)*, pages 496–545. Kinokuniya, Tokyo, 1986.

[11] S. Mori and S. Mukai. Erratum: "Classification of Fano 3-folds with $B_2 \geq 2$". *Manuscripta Math.*, 110(3):407, 2003.

[12] S. Mori and S. Mukai. Extremal rays and Fano 3-folds. In *The Fano Conference*, pages 37–50. Univ. Torino, Turin, 2004.

[13] The Sage Developers. *SageMath, the Sage Mathematics Software System (Version 8.4.0)*, 2018. `http://www.sagemath.org`.
